# The L-DOPA/Dopamine Pathway Transgenerationally Regulates Cuticular Melanization in the Pea Aphid *Acyrthosiphon pisum*

**DOI:** 10.3389/fcell.2020.00311

**Published:** 2020-05-05

**Authors:** Yi Zhang, Xing-Xing Wang, Hong-Gang Tian, Zhan-Feng Zhang, Zhu-Jun Feng, Zhan-Sheng Chen, Tong-Xian Liu

**Affiliations:** Key Laboratory of Integrated Pest Management on Crops in Northwestern Loess Plateau, Ministry of Agriculture, Northwest A&F University, Xianyang, China

**Keywords:** *Acyrthosiphon pisum*, L-DOPA, RNA interference, maternal effect, tyrosine hydroxylase, phenotypic plasticity

## Abstract

Maternal phenotypic regulations between different generations of aphid species help aphids to adapt to environmental challenges. The pea aphid *Acyrthosiphon pisum* has been used as a biological model for studies on phenotypic regulation for adaptation, and its alternative phenotypes are typically and physiologically based on maternal effects. We have observed an artificially induced and host-related maternal effect that may be a new aspect to consider in maternal regulation studies using *A. pisum*. Marked phenotypic changes in the cuticular melanization of daughter *A. pisum* were detected via *tyrosine hydroxylase* knockdown in the mothers during their period of host plants alternations. This phenotypic change was found to be both remarkable and repeatable. We performed several studies to understand its regulation and concluded that it may be controlled via the dopamine pathway. The downregulation and phenotypes observed were verified and described in detail. Additionally, based on histological and immunofluorescence analyses, the phenotypic changes caused by cuticular dysplasia were physiologically detected. Furthermore, we found that this abnormal development could not be reversed after birth. Transcriptome sequencing confirmed that this abnormal development represents a systemic developmental failure with numerous transcriptional changes, and chemical interventions suggested that transgenerational signals were not transferred through the nervous system. Our data show that transgenerational regulation (maternal effect) was responsible for the melanization failure. The developmental signals were received by the embryos from the mother aphids and were retained after birth. *APTH* RNAi disrupted the phenotypic determination process. We demonstrate that non-neuronal dopamine regulation plays a crucial role in the transgenerational phenotypic regulation of *A. pisum*. These results enhance our understanding of phenotyping via maternal regulation in aphids.

## Introduction

Environment-induced phenotypic changes are observed in many animal species and are an important physiological strategy that enables them to cope with environmental threats. Aphid species are normally serious agricultural pests that are highly adaptable (Van Emden and Harrington, 2017). The pea aphid *Acyrthosiphon pisum* (Harris) is utilized as a biological model of insect–plant interactions, phenotypic plasticity, and in symbiosis studies (Braendle et al., 2006). *A. pisum* has a transgenerational regulation system, and the biological features for their daughters can be modified between generations as different phenotypic development pathways, such as winged (alatae)–wingless (apterae) and sexual–asexual morphs, are determined by the mothers (Braendle et al., 2006; International Aphid Genomics Consortium, 2010). Winged and wingless *A. pisum* show diversity in their morphological, physiological, and behavioral features (Wratten, 1977; Sack and Stern, 2007). Furthermore, sexual and asexual individuals within this species differ mainly regarding their reproductive patterns and morphology (Miura et al., 2003). However, the mechanisms underlying the phenotypic regulations between different aphid generations are not yet fully understood.

The phenotypic controls and regulations of aphids are determined by the mother’s generation (Van Emden and Harrington, 2017). Asexual aphids parthenogenetically produce embryos that develop directly within them. They can regulate organ and tissue formation patterns that are related to the different phenotypes of their daughters. For example, in *A. pisum*, wing and sex determination occur prenatally (MacKay and Wellington, 1977; Brisson, 2010); therefore, the phenotype of the aphids does not tend to change within a single generation in response to environmental changes (Brisson, 2010). The phenotypic regulation system of the mother aphids is primarily based on environmental conditions, such as temperature, photoperiod, physical contact (aphids density), and host nutrition status (Braendle et al., 2006). Maternal effects are observed in many aphid species and affect more than one generation (Zehnder and Hunter, 2007; Jeffs and Leather, 2014; Hu et al., 2016). Besides phenotypic regulations, biological and ecological features such as population dynamics, fecundity, and interspecies relationships (with ant and parasitoids) may be maternally regulated (Zehnder and Hunter, 2007; Tegelaar et al., 2013; Slater et al., 2019). Previous studies of transgenerational signal transmissions showed that there was little or no yolk in the viviparous oocytes and embryos, and no chorion. This was most likely because in addition to being dispensable, an eggshell could interfere with the maternal provisioning of the developing embryos (Bermingham and Wilkinson, 2009; Ogawa and Miura, 2014). In studies of the physiological basis, however, juvenile hormone (JH) and insulin pathways were found to possibly contribute to this regulation, but studies in which JH titers were manipulated have shown inconsistent results, and further research is required (Braendle et al., 2006; Huybrechts et al., 2010; Ishikawa et al., 2012). In brief, maternal effects play important roles for aphid environmental adaptations, but their mechanisms are still unclear.

The environment-induced morphs of aphids exhibit biological and physiological differences in many respects, including their cuticular sclerotization and melanization levels (Ishikawa and Miura, 2007). Insect cuticles are primarily composed of regular arrangements of catecholamines (for melanization), lipids, cuticle proteins, and chitin; they are present in various cuticular layers, and interact with each other (Filshie, 1982; Vukusic and Sambles, 2003; Moussian et al., 2006; Arakane et al., 2009; Gorman and Arakane, 2010; Noh et al., 2016). Physicochemical interactions among them constitute the foundation of cuticle formation, body shape, and surface appearance (Filshie, 1982; Wigglesworth, 1990; Anderson, 1966, 2011; Van de Kamp and Greven, 2010). The production of catecholamines eventually leads to pigment depositions in the exocuticle or epicuticle layers, and tyrosine hydroxylase (TH; the rate-limiting enzyme of dopamine biosynthesis) plays an important role in melanization regulation (Arakane et al., 2009; Noh et al., 2016).

Previous studies have described the melanization process for cuticular sclerotization and melanization of insects (catecholamine metabolize), which can be summarized as follows. In the cuticle, TH converts tyrosine into L-3,4-dihydroxyphenylalanine (L-DOPA) in the epidermal cells. Thereafter, L-DOPA decarboxylase (DDC) converts L-DOPA into dopamine. Acyldopamines, *N*-acetyldopamine (NADA) and *N*-β-alanyldopamine (NBAD), are synthesized from dopamine via the actions of the arylalkylamine-*N*-acetyltransferase (aaNAT) and NBAD synthase (ebony), respectively, and are transported to the extracellular tissues. Laccases (laccase 2, lac2) catalyze melanization (pigmentation) and sclerotization in the cuticles and other tissues (Suderman et al., 2006; Noh et al., 2016). Numerous insect species that were subjected to *TH* RNA interference (RNAi) treatments were shown to exhibit a pale body color (Gorman and Arakane, 2010; Ma et al., 2011; Lee et al., 2015). L-DOPA and dopamine are key chemicals upstream of catecholamine regulatory system.

The L-DOPA, which is functional upstream in animal melanization regulatory and nervous systems, is also found in plants, including the broad bean *Vicia faba*, the hosts of *A. pisum*. Pea aphids attack a variety of legume crops, including *V. faba*, white clover (*Trifolium repens*), and alfalfa (*Medicago sativa*) (Kanvil et al., 2014). *V. faba* are known to contain high levels of L-DOPA (Longo et al., 1974; Ingle, 2003; Zhang et al., 2016), which is a non-protein amino acid that participates in numerous plant and animal metabolic processes (Wise, 1978; Smeets and González, 2000) and also functions as an important secondary metabolite in plant chemical defenses against herbivores (Huang et al., 2011; Zhang et al., 2016). However, adapted pea aphids can sequester L-DOPA and use it for wound healing and UV-A resistance, which are processes related to melanization (Zhang et al., 2016). The L-DOPA environment could, thus, be important and aid the pea aphid in its adaptations for survival. This could occur either by balancing the L-DOPA self-synthesis and assimilation for stabilizing metabolic processes or by modifying the L-DOPA/dopamine biometabolic pathway. We have aimed to study in detail the transcriptomic profiles of the candidate genes in this pathway.

In the present study, we have detected remarkable and repeatable RNAi-related phenotypic changes in *A. pisum*, under hosts alternations. The phenomenon has been observed in response to *A. pisum TH* (*APTH*) knockdowns in the nymphs of RNAi-treated mothers during the period of transition between host plants (with high L-DOPA to low L-DOPA content). In response to this treatment, perturbations of the cuticle melanization and nymph development were observed. RNAi is widely used in gene function studies (Hannon, 2002), but its repression efficiency varies considerably among different insect species (Singh et al., 2017). Studies have shown that dsRNAs in the body fluid of *A. pisum* can be degraded, resulting in variable genetic downregulations (Huvenne and Smagghe, 2010; Christiaens et al., 2014; Singh et al., 2017).

To elucidate the mechanisms underlying the unexpected phenotypic changes in *A. pisum*, we investigated the transcriptional changes in the target genes, in response to the *APTH*-RNAi, and subsequently analyzed the L-DOPA and dopamine levels using liquid chromatography–mass spectrometry (LC/MS). We also performed detailed analyses of the phenotypic and biological changes in the nymphs, to compare the induced phenotypic changes and normal maternal phenotypic regulations. Histological examinations, immunofluorescence detection, transcriptomic analyses, and chemical interventions were also performed to understand the regulatory mechanisms underlying these transgenerational phenotypic changes.

## Materials and Methods

### Insects and Plants

Red *A. pisum* were collected from *M. sativa* plants in Lanzhou, Gansu Province, China (N36°07′7″, E103°42′20″; Aug 2015) and reared on *V. faba* in Shaanxi, China, for approximately 5 years. Prior to the experiments, the aphids were cultured in low densities on *V. faba* and *T. repens* under long-day conditions (16:8 h L:D; 20 ± 1°C) for more than 30 generations at the Key Laboratory of Applied Entomology, Northwest A&F University, Yangling, Shaanxi, China.

Only wingless aphids were used in the experiments. They were replenished by rearing all the insects at densities in excess of 30 aphids/plant, for more than three generations. The aphids during the host transition period were used for the RNAi experiments. The newborn aphids (approximately 100 individuals for each experiment) on the *V. faba* (high L-DOPA content; Supplementary Figure S10) were immediately transferred and reared on *T. repens* (low L-DOPA content; Supplementary Figure S10) until they reached the adult stage. Phenotypic changes could also be observed in the *A. pisum* reared on *M. sativa* (also low L-DOPA content) during host plant alternations and RNAi from *V. faba*. However, given the impracticality of working with the much smaller *M. sativa* leaves, we selected *T. repens* (fit-size leaves for cells of 24-well plate) for the further experiments.

### RNAi of *APTH* in *A. pisum*

The dsRNA of *APTH* was prepared for downregulation investigations, to modify the biosynthesis of L-DOPA. The dsRNA of the *lymphotoxin-alpha* gene (*lta*; Gene ID: 16992) of *Mus musculus* was used as a control (Chen et al., 2016). The synthesis and delivery methods (Barron et al., 2007; Zhang et al., 2016) of the dsRNAs are detailed in S1.1 (Supplementary Material), and the primers used to synthesize the *APTH* and *mus-lta* dsRNAs (designated as ds-TH1, ds-TH2, and ds-lta, respectively) are shown in Supplementary Table S1 and Supplementary Figure S1.

### L-DOPA and Dopamine Extractions and Assays

The injected aphids were reared on *T. repens* for 72 h after treatment and subsequently collected for LC/MS analysis. Newborn nymphs (within 30 min after birth) were collected from mothers between 72 and 96 h after the dsRNA treatments and prepared for LC/MS analysis. This methodology is detailed in S1.2 (Supplementary Material).

### Observations of Phenotypic and Biological Changes

#### Phenotype Determination

Treated mothers and newborn nymphs were reared separately. Images of the mothers were captured 72 h after injection, whereas those of the nymphs were captured at each developmental stage. Individuals were prepared for imaging. Digital images were acquired using a Panasonic DMC-GH4 digital camera (Panasonic, Osaka, Japan) and a SDPTOP-SZN71 microscope system (Sunny, Hangzhou, Zhejiang, China).

#### Biological Parameters

The following biological parameters of the treated mothers (ds-TH and ds-lta) and their daughters were analyzed: proportion of abnormal producers (mothers); life span, survival rate, and duration of development for each stage (nymphs). This is further detailed in S1.3 (Supplementary Material).

#### Video Recording

Newborn (first instar) and new molted (second instar) nymphs were prepared for video recording (see S3 for more information, Supplementary Material).

### Analysis of the Cuticle Morphology

Histological sectioning, staining (HE) and immunofluorescence were performed for abnormal (including low-tanning and over-tanning nymphs; Supplementary Figure S11) and control nymphs obtained from the ds-TH and ds-lta treatments, respectively. Five samples of the second thoracic (T2) segments of the third-instar of *A. pisum* from each treatment were selected for hematoxylin–eosin (HE) staining, and five samples of the cross-sections of the T2 segments of the third-instar nymphs from each treatment were selected for immunofluorescence assays. Transverse longitudinal sections, from the head to the tail, of the treated mother aphids were also collected 48 h after the dsRNA injection for immunofluorescence experiments. The method is detailed in S1.3 (Supplementary Material). Digital images were acquired using a Nikon DS-Ri1 camera (Nikon, Tokyo, Japan), a Nikon 80i microscope system (Nikon, Tokyo, Japan), and Nis-Elements v. 3.22.14 (Build 736, Nikon, Tokyo, Japan). Cuticle thickness was determined from the digital images. All images showing Cy^TM^3 fluorescence were captured and filtered using the default settings (brightness, contrast, and saturation) of software for comparison.

### Transcriptome Sequencing

To analyze the systemic transcriptional changes between the two phenotypes, abnormal (including low-tanning and over-tanning nymphs; Supplementary Figure S11) and control nymphs obtained from the ds-TH and ds-lta treatments, respectively, were prepared for transcriptome sequencing. Since the embryos (inside the nymphs) could affect the transcriptomic data, the individuals were dissected and only the head with the antennae and legs were collected (Supplementary Figure S12). Approximately 400 samples from three developmental stages per treatment were frozen in liquid nitrogen for RNA extraction. As the RNA content in each sample tissue was relatively low, only one RNA sample per treatment (ds-TH and ds-lta) was successfully prepared. Extracted RNA was sent to the Wuhan Bioacme Corporation (Wuhan, China;^1^) for transcriptome sequencing and preliminary analysis (Supplementary Table S3). We also selected candidate genes (demonstrating relatively highly changed expression patterns) and analyzed the transcriptional changes in the newborn daughters of the abnormal nymph producers on the 3rd day after the dsRNA treatment. The sample collection protocol is detailed in S1.1 (Supplementary Material).

### Testing the Dopamine Transition Signals of the Nervous System

The dopamine/L-DOPA biosynthesis inhibitor (metirosine) and dopamine receptor antagonists (SCH23390, Sulpiride and Pimozide) were injected into *A. pisum* to investigate the transition signals of the nervous system underlying this transgenerational phenomenon. The method is detailed in S1.4 (Supplementary Material), and the daughters of the treated aphids were reared for phenotypic detection (as described in section “Observations of phenotypic and biological changes”).

### Statistical Analyses

*APTH* transcriptomic data from the different treatments and aphids were subjected to Mann–Whitney *U*-tests (non-parametric). Values of the chemical amounts, body lengths, and developmental times were subjected to one-way analysis of variance. Differences among means were calculated using Duncan’s test at a significance level of *P* < 0.05. Quantitative analyses of the L-DOPA and dopamine and the proportions of the abnormal nymph producers were subjected to a Student’s *t*-test where *P* < 0.05 indicated significance. The survival data were analyzed using an χ^2^ test where *P* < 0.05 also indicated significance. All data were processed using SPSS v. 22 (IBM Corp., Armonk, NY, United States). Immunofluorescence images were obtained and analyzed using CaseViewer (V 2.0, 3DHISTECH Ltd., H-1141 Budapest, Hungary). Charts and diagrams were constructed with Microsoft^®^ Excel 2016 MSO (16.0.4266.1003; Redmond, WA, United States) and GraphPad Prism (V 8.0.2; San Diego, CA, United States).

## Results

### *APTH* Knockdown by RNAi in the Mother Aphids

#### Differences in *APTH* Repression in Different Parts of *A. pisum*

**FIGURE 1:**
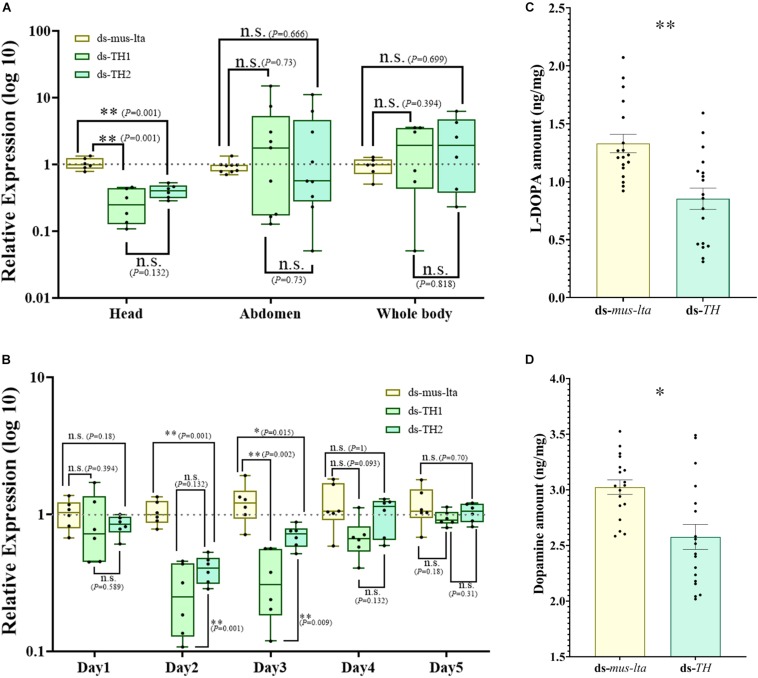
*Tyrosine hydroxylase* RNAi in *Acyrthosiphon pisum*. **(A)** *APTH* transcriptional response to *APTH* RNAi in the head, abdomen, and whole body. n.s. indicates no significant difference according to Mann-Whitney *U*-test (non-parametric; *P < 0.05, **P < 0.001). Irregular *APTH* transcriptional response to *APTH* RNAi in the abdomen. n.s. indicates no significant difference. **(B)** Effect of RNAi suppression on *APTH* expression. n.s. indicates no significant difference according to Mann-Whitney *U*-test (non-parametric; *P < 0.05, **P < 0.001). Differences in L-DOPA **(C)** and dopamine **(D)** levels between aphids treated with *APTH* and those treated with *lta* RNAi. Each value represents the mean ± SEM; n.s. indicates no significant difference according to Student’s *t*-test (**P < 0.001).

After the injection of dsRNAs (ds-TH1, ds-TH2, and ds-lta) into *A. pisum*, a significant reduction in the expression of *APTH*-RNAi was observed in the head on the second day (Figure 1A; *P* values are marked between the bars). In contrast, no significant transcriptional differences were detected in the abdomen (Figure 1A; *P* values are marked between the bars) or the whole body (Figure 1A; *P* values are marked between bars). Additional experiments with more replicates revealed that the abdominal *APTH* expression was unstable with the ds-TH1 and ds-TH2 treatments (Figure 1A; *P* values are marked between bars).

For more information and physiological background, the transcriptional differences in the expression of *APTH* between the different developmental stages are shown in Supplementary Figures S2,S3 (head and abdomen, respectively) and the expression changes of *DDC* under *APTH*-RNAi are shown in Supplementary Figures S5,S6.

#### Duration of *APTH* Repression in the Heads of *A. pisum*

The downregulation of *APTH* in the head was relatively stable, and therefore, we analyzed the duration of *APTH* repression in the head. No significant reduction in *APTH* expression was detected in the heads of the dsRNA-treated aphids on the first day (Figure 1B; *P* values are marked between the bars). The dsRNA treatment reduced *APTH* expression on days 2 and 3 (Figure 1B; *P* values are marked between the bars). Thereafter, *APTH* expression was indistinguishable from that of the control individuals (Figure 1B; *P* values are marked between the bars). Additionally, the ds-TH1 treatment was slightly more effective than the ds-TH2 treatment.

#### L-DOPA and Dopamine Content of Mother Aphids With *APTH* Downregulation

LC/MS results showed that the L-DOPA content significantly decreased in aphids with *APTH* downregulation (*t* = 3.908, df = 34, *P* < 0.001; Figure 1C). A significant decrease in dopamine was also observed under the same conditions (*t* = 3.438, df = 34, *P* = 0.002; Figure 1D).

#### TH Protein Analysis Using RNAi in Mother Aphids

**FIGURE 2:**
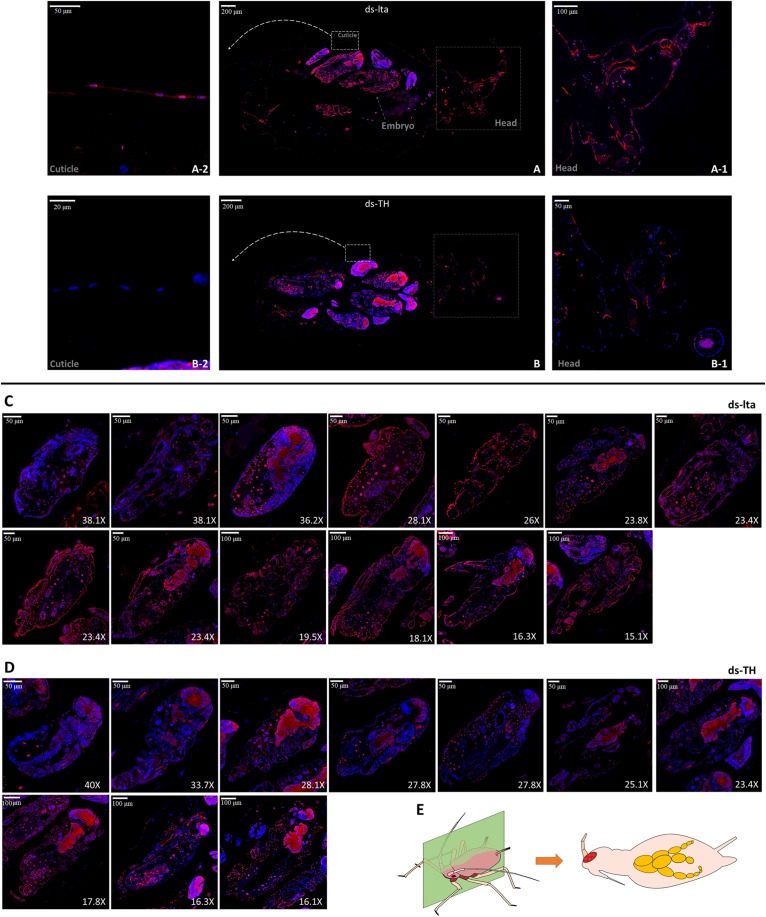
TH immunofluorescence of treated mothers. The distribution of TH protein in *lta* dsRNA treated **(A)** and *APTH* dsRNA treated **(B)** adult aphids was determined by immunofluorescence. Anti-TH antibodies were detected using Cy3-conjugated rabbit IgG antibodies (red); Nuclei were stained with DAPI (blue). Head and cuticle were magnified and showed (ds-lta: head, A-1, cuticle, A-2; ds-TH: head, B-1, cuticle, B-2). All longitudinal slitting embryos inside [13 embryos/5 mothers from ds-lta treatments, **(C)**; and 10 embryos/5 mothers from ds-TH treatments, **(D)**] were selected and ordered by sizes. Transverse longitudinal sections from head to tail of *Acyrthosiphon pisum* samples **(E)**.

The presence of the TH protein in the mother aphids and embryos treated with ds-TH and ds-lta was determined by immunofluorescence. Proteins were observed to be immune-positive in the mothers’ cuticles and mostly in the embryos (Figure 2). Compared with the control treatments, in TH-RNAi–treated aphids, the TH fluorescence was detected only sparsely in the cuticles (Figure 2A). The fluorescence outline (red) of the head was difficult to identify in individuals with *APTH* downregulation.

Differences in immunofluorescence could also be observed in the embryos (Figures 2C,D). All embryos that could be identified in the longitudinal cuttings were collected and arranged by size for further comparison. Differences in the TH protein distributions could be detected in the embryo cuticles during the late developmental stages (from 15× to 30×; 400–1000 μm) between the two dsRNAs treatments, especially in the red fluorescence outlines (cuticle) of the embryo bodies. However, based on the detection of a strong fluorescence inside the embryos after the two treatments, we assume that the TH protein levels were not affected in the nervous system (Figures 2C,D). Original images and more replicates are provided in the supplemental information (*original images.rar*).

### Phenotype Observations

**FIGURE 3:**
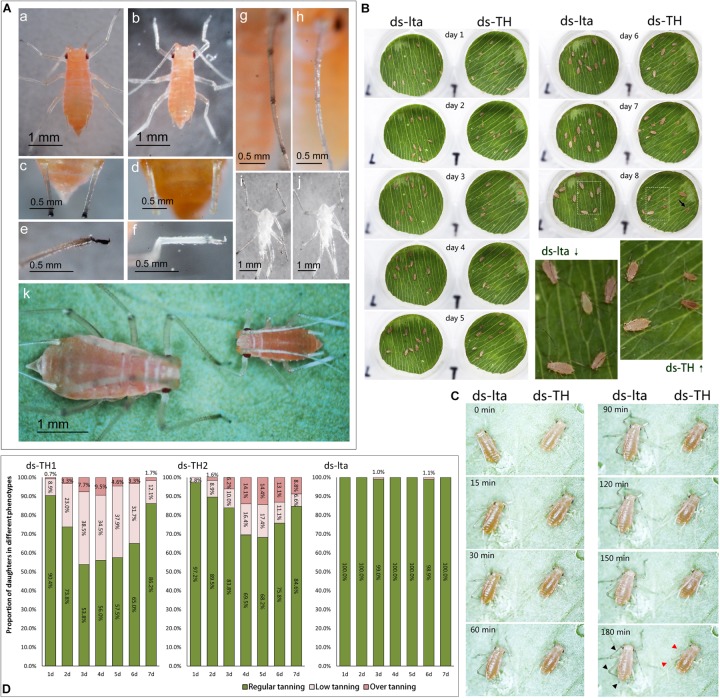
Low or abnormal melanization in nymphs of *Acyrthosiphon pisum*. The second-instar nymphs selected for comparison were 12 h old **(A)**. Appearances of control (WT, wild type) and abnormally melanized aphids (a,b); relative differences in melanization of the cornicles of WT and abnormally melanized aphids (c.d); relative differences in melanization of the legs of WT and abnormally melanized aphids (e,f); relative differences in melanization in the antennae of WT and abnormally melanized aphids (g,h); relative differences in melanization of the exuviae of WT and abnormally melanized aphids (i,j); relative differences in the appearances of WT and abnormally melanized aphids of the same age (8 d post-hatching; k). Comparison of wild type (WT) and abnormally melanized aphids after 8 d of monitoring **(B)**; comparison of early melanization in WT and abnormally melanized aphids after 3 h of monitoring **(C)**; the arrow indicates an abnormal nymph at day 8; the red arrowhead indicates an abnormal melanin deposit and the black arrowhead indicates a melanin deposit in the control. Proportions of abnormal melanization in all nymphs after 7 d of monitoring mothers treated with different dsRNAs (ds-TH1, ds-TH2, and ds-lta, **D**); abnormal nymph producers selected from mothers treated with ds-TH1 and ds-TH2.

Compared with the normal control mothers, the fourth-instar mothers with *APTH* knockdown did not differ in appearance. However, phenotypic alterations were observed in their daughters. The melanization levels in the heads, antennae, corniculi, and legs of the nymphs were substantially lower than those of their mothers (Figure 3A). Their legs were markedly curved and could not support their bodies (Figures 3Ae, Af). Low melanization levels were also evident in their exuviae (Figures 3Ai, Aj). After 8 d, most of the abnormal nymphs that reached the third instar failed to mature to the next developmental stage, whereas the control nymphs continued to develop into adults (Figure 3Ak). Excessive tanning could also be detected in the legs of the nymphs derived from the TH-RNAi mothers. Abnormal tanning (low- and over- tanning) may lead to a failure to molt (Supplementary Figure S11). The unabsorbed and unshed exuviae succumbed to fungal infections while still attached to the body (Supplementary Figure S11).

In the development monitoring experiments, the nymphs from the mothers subjected to the two treatments displayed differences in their body sizes and survival rates, during the 10-day monitoring period (Figure 3B; the original images are provided in supplemental information, *original images.rar*). A reduction in melanization was also detected by monitoring the early tanning after nymph molting. Nymphs subjected to different treatments and molting simultaneously exhibited very different tanning rates. Control aphids tanned faster than those treated with ds-TH (Figure 3C; original video 1: *1 instar 50X.mkv*; original video 2: *2 instar 20X.mkv*; Supplemental information). In addition, abnormal nymphs developed by day 7 after the TH dsRNA injections (ds-TH1 and ds-TH2) showed that most of the abnormal aphids were low-tanning individuals. However, two control nymphs with abnormal tanning were also observed on days 3 and 6 (Figure 3D).

### Transcriptional and Biological Analyses of the Daughter Aphids

#### *APTH* Expression Analysis

**FIGURE 4:**
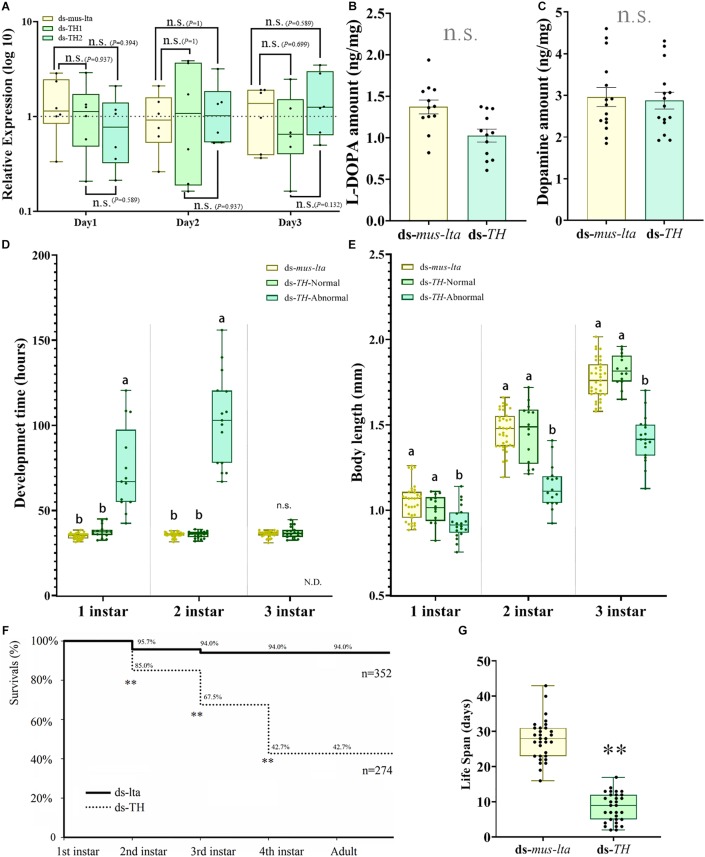
Transcriptional, biochemical, and biological analyses of abnormal *Acyrthosiphon pisum* nymphs. **(A)** *APTH* transcription levels in 3-d-old nymphs derived from mothers treated with different dsRNAs. n.s. indicates no significant difference according to Mann-Whitney *U*-test (non-parametric; *P < 0.05, **P < 0.001). Relative differences in L-DOPA **(B)** and dopamine **(C)** levels between healthy and abnormal nymphs. n.s. indicates no significant difference according to Student’s *t*-test. Comparative differences in development time **(D)** and body length **(E)** among healthy and abnormal nymphs derived from mothers treated with ds-TH and ds-lta. Different letters within the same fig indicate significant differences in the values (ANOVA; Duncan’s test; *P* < 0.05), n.s. indicates no significant difference. N.D. (in D) denotes no data. Differences in survival rate **(F)** between healthy and abnormal nymphs derived from mothers administered ds-TH and ds-lta; differences in lifespan **(G)** between healthy and abnormal nymphs derived from mothers treated with ds-TH and ds-lta. ** in F indicates significant differences in the values (χ^2^ test; *P* < 0.001). ** in G indicates significant differences in the values by Student’s *t*-test (*P* < 0.001).

*APTH* transcription levels in the daughter aphids were not affected by the RNAi treatments in the mothers. *APTH* expression levels in the newborn daughter nymphs (within 30 min after laying and no feeding) did not significantly change over 3 days after the dsRNAs treatments (Figure 4A; *P* values are marked between the bars).

#### L-DOPA and Dopamine Levels

There were no significant differences in the L-DOPA and dopamine levels between the nymphs laid by the mothers from the ds-TH and the ds-lta treatments (L-DOPA: *t* = −0.479, df = 28, *P* = 0.636; Figure 4B; dopamine: *t* = 0.284, df = 28, *P* = 0.778; Figure 4C).

#### Development Time and Body Size at Each Stage

The development times of the abnormal nymphs derived from the *APTH*-RNAi–treated mothers were significantly longer (without antenna) than those of the control nymphs in the first and second instars (1st instar: *F* = 52.139, df = 2, 56, *P* < 0.001; 2nd instar: *F* = 158.137, df = 2, 62, *P* < 0.001; Figure 4D). In contrast, the development times of the healthy nymphs (no obvious phenotypic changes) derived from the ds-TH–treated mothers did not significantly differ from those of the control. The survival rates of the abnormal third-instar nymphs were extremely low, and very few of these nymphs reached the fourth instar. The surviving nymphs from both the treatments had similar development times in the third instar (*t* = −1.418, df = 47, *P* = 0.163; Figure 4D).

The body sizes of the abnormal nymphs derived from the ds-TH–treated mothers were significantly smaller than those of the healthy individuals derived from these mothers (ds-TH) and of the control nymphs (ds-lta). This phenomenon was observed at all three developmental stages examined (1st instar: *F* = 8.978, df = 2, 66, *P* < 0.001; 2nd instar: *F* = 37.569, df = 2, 62, *P* < 0.001; and 3rd instar: *F* = 63.932, df = 2, 64, *P* < 0.001; Figure 4E and Figure 3B).

#### Lifespan and Survival Rates at Each Developmental Stage

The survival rates of the nymphs derived from the ds-TH–treated mothers were significantly lower than those of the control aphids in the first three developmental stages (1st instar: χ^2^ = 21.664, df = 1, *P* < 0.001; 2nd instar: χ^2^ = 56.895, df = 1, *P* < 0.001; and 3rd instar: χ^2^ = 140.132, df = 1, *P* < 0.001; Figure 4F). No abnormal nymphs (individuals showed low-tanning and over-tanning) reached the fourth instar. However, all healthy nymphs (ds-TH) survived through the last two stages. The lifespan of the abnormal nymphs was only half that of the control nymphs, and the difference was significant (*t* = −15.135, df = 60, *P* < 0.001; Figure 4G).

### Cuticle Morphology in the Daughter Aphids of Treated Mothers

**FIGURE 5:**
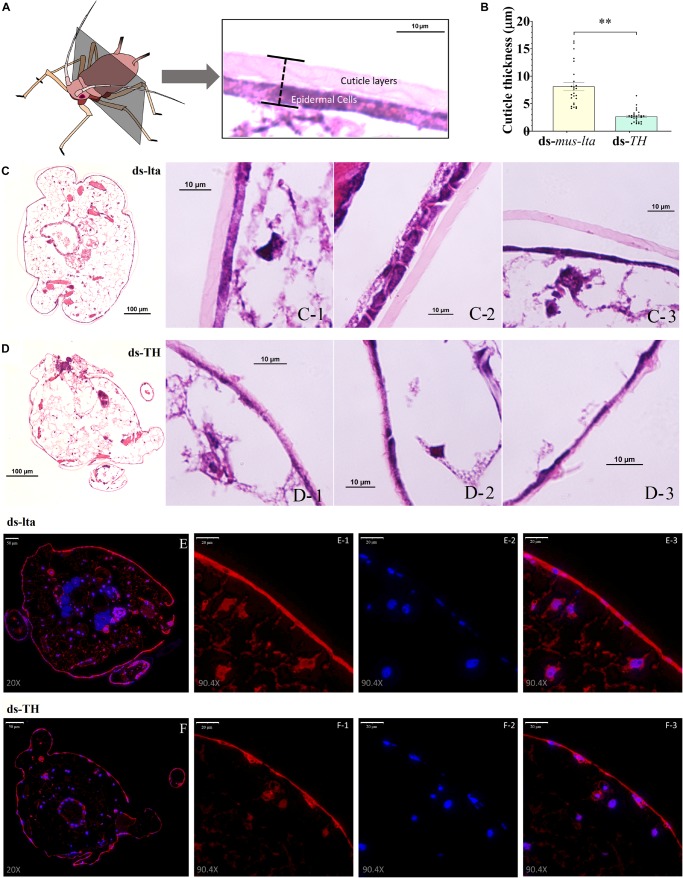
Histology and TH immunofluorescence of daughters. Hematoxylin and eosin (H&E)-stained transverse sections of the cuticle at segment T2 **(A)** from third-instar pea aphid (*Acyrthosiphon pisum*) at × 10 and × 100 magnification. **(B)** Relative differences in cuticle thickness between healthy and abnormal aphids. Each value represents the mean ± SEM; Student’s *t-*test (**P < 0.001). **(C)** H&E-stained histological sections of healthy nymphs (control). **(D)** H&E-stained histological sections of nymphs with abnormal cuticles. The distribution of TH protein in the cuticle of healthy **(E)** and abnormal **(F)** aphids was determined by immunofluorescence (× 20 and × 90.4 magnification). Anti-TH antibodies were detected using Cy3-conjugated rabbit IgG antibodies (red, E-1 and F-1, × 90.4 magnification); Nuclei were stained with DAPI (blue, E-2 and F-2, × 90.4); and merged images are shown in E-3 and F-3 (×90.4 magnification).

Histological analysis of the cuticle sections revealed that the cuticles of the daughter aphids (ds-TH) consisted of thinner layers (cuticle and epidermis, *t* = 7.104, df = 50, *P* < 0.001; Figure 5B) than those of the controls (ds-lta). Tissue abnormalities were also observed in the cuticle layers (Figure 5). The distinct cuticle layers were detected in the control nymphs (Figure 5C) but were indistinguishable from those derived from the ds-TH–treated mothers (Figure 5D). The microstructures of the epidermal cell layers in the latter (ds-TH) were also poorly defined (Figure 5).

The distribution of the TH proteins in the cuticles of the healthy and abnormal daughters was determined by immunofluorescence. Although the TH staining could be observed in the cuticle epidermal cells of the nymphs in both treatments (Figures 5E,F), the distribution of the TH protein was irregular in the cuticles of the abnormal nymphs (Figure 5F).

### Relative Differences in Gene Expression Based on Transcriptome Sequencing

**FIGURE 6:**
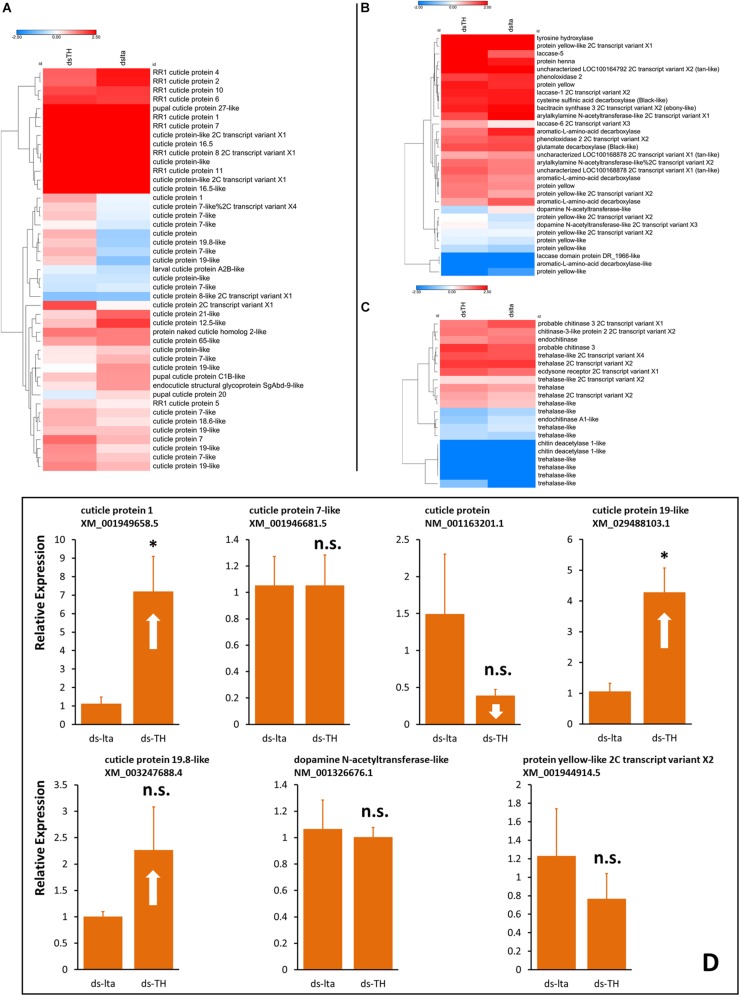
Transcription analysis of daughters. Heatmaps of transcriptional differences in the candidate genes associated with cuticle proteins **(A)**, melanization **(B)**, and chitin biosynthesis **(C)**. Data were obtained from transcriptome sequencing of healthy and abnormal *Acyrthosiphon pisum* nymphs. Each sample prepared for RNA extraction consisted of more than 400 aphids without abdomens. Hierarchical clustering was performed using the Euclidean distance and the average linkage method. Transcription changes of selected genes from transcriptome results in new born aphids (whole body) were verified by qRT-PCR **(D)**, samples were collected from new daughters of abnormal-nymph-producers at the 3rd day after treatment. n.s. indicates no significant difference according to Student’s *t*-test (*P < 0.05).

The transcriptomic results showed the transcriptional changes of the numerous genes between the two different daughter aphids. Heatmaps were constructed using candidate genes from cuticle proteins, melanization pathways, and chitin biosynthesis. The results showed differences in the transcription levels of these genes between the abnormally tanned (low- and over-tanned) and healthy individuals. The cuticle protein genes (CPs) were either upregulated or downregulated in response to the ds-TH treatment (Figure 6A). The transcription levels of the several candidate melanization genes were also altered. However, the expression levels of *TH*, the target gene of dsRNA, remained unchanged (Figure 6B). Only a slight alteration in the transcription levels was observed for chitin biosynthesis genes (Figure 6C).

Transcriptional verification by qRT-PCR of the selected candidate genes that exhibited relatively large exchange rate changes. The transcriptional results (qRT-PCR) showed different results from those of the transcriptomic data. Some results were similar, such as changes to XM_001949658.5 and XM_029488103.1 (XM_001949658.5, *t* = −3.168, df = 4, *P* = 0.034; XM_029488103.1, *t* = −3.887, df = 4, *P* = 0.018; Figure 6D). However, some other results of selected candidate genes that exhibited strong up- or downregulations in the transcriptomic analysis did not show a significant change in our transcriptional verifications (XM_001946681.5, *t* = 0.002, df = 4, *P* = 0.999; NM_001163201.1, *t* = 1.354, df = 4, *P* = 0.247; XM_003247688.4, *t* = 11.416, df = 4, *P* = 0.201; NM_001326676.1, *t* = 5.681, df = 4, *P* = 0.809; XM_001944914.5, *t* = 0.807, df = 4, *P* = 0.465; Figure 6D). Additional details are available in Figures S12–S16 and the Supplementary Material (*Transcriptome sequencing.rar*).

### Injection of a Dopamine Biosynthesis Inhibitor and Receptor Antagonists

To investigate whether dopamine-related pathways of the nervous system were involved in this transgenerational control system, we treated individual specimens with an inhibitor of dopamine biosynthesis or with receptor antagonists. Only individuals treated with the dopamine biosynthesis inhibitor (metirosine) gave birth to abnormal nymphs. We found that 7 of the 32 mothers were abnormal nymph producers, and phenotypic changes were detected in their daughters (Table 1). All individuals treated with dopamine receptor antagonists (SCH23390, Sulpiride and Pimozide) gave birth to normal and healthy offspring (Table 1). Typical low melanization of nymphs (no over-tanning individual found) were observed in metirosine-treated samples; this was similar to the phenotypic changes described previously in the daughter aphids of mothers with *APTH* knockdown (subsection “Phenotype observations”).

**TABLE 1:** Abnormal offspring detection of *Acyrthosiphon pisum* under dsRNAs and chemicals (dopamine biosynthesis inhibitor and receptor antagonists) injections.

	Descriptions	Proportion of abnormal daughters (abnormal aphids/all daughters)		
		Day 3**	Day 4**	Day 5**
dsRNAs	ds-TH1* (dsRNA of *APTH*)	66/143 (46.15%)	51/116 (43.97%)	37/87 (42.53%)
	ds-TH2* (dsRNA of *APTH*)	21/130 (16.15%)	39/128 (30.47%)	42/132 (31.82%)
	ds-lta* (dsRNA control)	1/100 (1.00%)	0/95 (0)	0/113 (0)
Chemicals	Metirosine (dopamine biosynthesis inhibitor)	30/106 (28.30%)	11/119 (9.24%)	11/131 (8.40%)
	SCH23390 (D1 antagonist)	0/97 (0)	0/109 (0)	0/102 (0)
	Sulpiride (D2 and D3 antagonist)	0/73 (0)	0/90 (0)	0/90 (0)
	Pimozide (D2, D3, and D4 antagonist)	0/90 (0)	0/63 (0)	1/101 (0.99%)
	Saline (0.9%) (control)	0/146 (0)	0/123 (0)	0/101 (0)

*See Figure 3D for more details about dsRNAs injections. **After injections.

## Discussion

Many of the phenotypic features of *A. pisum* are maternally determined and can lead to phenotypic changes in their daughters. In this study, artificially induced (RNAi) and host-related (under host transition period) transgenerational phenotypic regulations in *A. pisum* were observed. These findings are particularly useful for furthering our understanding of the mechanisms underlying the phenotypic determination in aphids. We detected strong, predictable, repeatable, and consistent phenotypic changes in the daughter aphids of the treated individuals. Low and high levels of cuticular melanization and sclerotization were detected in the nymphs. The periods of host transition, from feeding on *V. faba* (high L-DOPA content) to the *T. repens* (low L-DOPA content), and simultaneous treatments with *APTH* (converts L-tyrosine to L-DOPA) dsRNAs were both essential for this phenomenon. We suggested that the maternal effects played an important role, and a transgenerational system based on dopamine/L-DOPA signals between the mother and embryos could exist. This transgenerational regulation may be related to the phenotypic plasticity of the pea aphid but requires further investigation to improve our understanding.

*TH*, the target gene silenced by RNAi in this study, is a key regulatory enzyme upstream of melanization pathway (Hearing et al., 1980; Ma et al., 2011). Irregular and disordered transcriptional feedback in the abdomen showed strong but unclear dopamine-related regulation response patterns, and we hypothesize that this reaction was caused by the introduction of the *APTH* dsRNA. In this experiment, the dietary intake of the L-DOPA in the aphids was markedly reduced (host change). This severe reduction in L-DOPA assimilation could promote a reorganization of the internal biochemical environment. The reduction in L-DOPA content may have triggered endogenous L-DOPA biosynthesis and maintained its downstream reactions. The dopamine pathway is moderately susceptible to imbalances, and even weak interference from exogenous dsRNA could disrupt the transmission of molecular signals and cause irregular and disordered expression feedback of *APTH* in *A. pisum*.

Further analysis of the distribution of *TH* confirmed that *APTH* was downregulated in the insect cuticle and explained the irregular transcriptional feedback in the abdomen. Distribution differences of TH were detected in the developing cuticle tissues of the embryos. These results are consistent with the findings from a study using microarrays and *A. pisum* (Rabatel et al., 2013) and another study showing that external dsRNA could spread throughout the whole body in aphids (Wang et al., 2015). The nervous systems in embryos with strong positive fluorescence might be responsible for the irregular expression in the abdomen, and it is also possible that the dopamine-based neurological systems displayed strong feedback to the RNAi performance.

When *APTH* transcription was modified in the mothers, the disordered melanization phenotype was subsequently detected in the daughters. This indicated that the melanization failure is a systemic disorder of the daughter aphids’ cuticle. Studies on the biological parameters from the abnormal aphids revealed that a maternal effect might determine this phenotype. Combining the results of *APTH* transcription detection and L-DOPA/dopamine content assays, which did not exhibit any significant differences, for the abnormal daughters, no transgenerational RNAi was observed. Furthermore, treated mothers continued to produce abnormal offspring even after the *APTH*-RNAi effect had weakened or disappeared altogether. Consequently, the phenotypic changes in the daughters may show long-term persistence and, generally, are irreversible. These conflicting results reflect the complexity of this phenomenon.

Furthermore, the daughter aphids with the abnormal phenotypes could not revert to the normal phenotypes at any subsequent stage in their lives, suggesting that these phenotypic changes were not directly induced by the RNAi (which could be recovered). Further experiments with the nymphs, including transcriptional analysis, L-DOPA/dopamine content analysis, and transcriptome sequencing, supported this conclusion. Melanization rate, body size, development time at each stage, and lifespan were markedly different between the abnormal and control nymphs. The abnormal aphids are born with signals that determine their future developmental patterns. This phenomenon is similar to the determination of wing and sexual dimorphism in aphids and is manifested as a maternal effect (Braendle et al., 2006; Dombrovsky et al., 2009; Brisson, 2010). Winged and sexual individuals differ from the typical parthenogenetic individuals in terms of their biological traits and appearance, including cuticular sclerotization and melanization (Kring, 1977; Ishikawa and Miura, 2007; Brisson, 2010). These differences are based on L-DOPA/dopamine reactions, and consequently, winged aphids are normally heavier than wingless aphids (Ishikawa and Miura, 2007).

We assumed that there was a connection between this induced phenotypic change and natural phenotypic regulations, as they may share a similar regulation pathway. Combining the results discussed above, several similarities were observed between our induced phenotypic changes and the natural maternal dimorphisms: (1) a certain proportion of the nymphs showed phenotypic changes (Figure 3; Müller et al., 2001; Wang et al., 2016); (2) the appearance of anomalous phenotypes in the offspring decreased over time, possibly because of signal attenuation in the mothers (Figure 3; Sutherland, 1969; Müller et al., 2001); and (3) the observed phenotypes in the transformed nymphs were generally permanent and irreversible (Figure 3). These similarities suggest that the phenotypic changes induced by the maternal *APTH*-RNAi was an atypical maternal effect in aphids. The fact that densely stimulated (physical contact) mothers can produce winged nymphs (irreversible phenotypic changes), whereas nutritional or photoperiod stimulations may induce mothers to generate sexual nymphs (irreversible phenotypic changes), reflect their adaptations to environmental changes (Nunes and Hardie, 1996; Miura et al., 2003; Sack and Stern, 2007; Dombrovsky et al., 2009).

The transcriptome sequencing results supported our hypothesis, as numerous transcriptional changes were observed, but candidate melanization gene expressions were relatively stable between the two samples. Heatmaps revealed no obvious differences between the abnormal and control nymphs with respect to the expression of the numerous candidate melanization genes (including *TH*) upstream of melanization regulation. In contrast, several genes downstream from *APTH*, including *laccase-like*, *yellow-like*, and *dopamine N-acetyltransferase-like* (Andersen, 2010), were either upregulated or downregulated in the abnormal nymphs in comparison to that in the control nymphs. Several *CPs* (Andersen et al., 1995) exhibited obvious changes between the abnormal and control nymphs, and these observations corroborated the identified microstructural collapses of the cuticle. The abnormally transcribed genes may be essential for cuticle formation, and their disruptions could cause the cuticle structures to collapse. The expression levels of several candidate chitin biosynthesis genes (Merzendorfer, 2006) in the abnormal nymphs were also slightly altered relative to those in the control nymphs. Our transcriptome sequencing results showed more changes than those that could be transcriptionally verified (Supplementary Figure S16). We also verified the transcriptional changes of the candidate genes (selected from transcriptome data) in the newborn aphids by qRT-PCR and found that while some candidate genes showed results similar to the transcriptome analysis (such as XM_001949658.5, XM_029488103.1, and XM_003247688.4), other genes (such as NM_001326676.1 and XM_001944914.5 of the melanization pathway) did not. We suggest that some genes might be directly regulated by the mothers, and daughter aphids that are born with transcriptional anomalies would not be able to revert to healthy individuals during further development processes. In contrast, other transcriptional changed genes may be downstream regulated after birth, and consequently, changes in their expression levels may not be detected in present study. However, the inefficiency of the RNAi of *A. pisum* made it difficult to further confirm the functions of the candidate *CPs* and reproduce the desired phenotypic change within a single generation by modifying these genes.

The malfunctioning cuticular layer observed with the transcriptional changes directly explained the physiological causes of these phenotypes. Cuticular dysplasia with microstructural collapse in *A. pisum* could result in abnormal melanization, which manifests as poor tanning. Analysis of the histological sections indicated that the cuticle and epidermal cells had thinned considerably, and there were anomalies in the cuticle layer, which is the site of melanin precipitation (Brey et al., 1985; Moussian, 2013). Malformed cuticular layers cannot support melanization, even with enough substrates (L-DOPA and dopamine) and fully functional enzymes (including TH). Therefore, abnormal melanization may account for the observed phenotypic changes. The presence of the TH protein negated the RNA intergenerational interference and the irregular distribution of the TH protein provided evidence for the collapse of the cuticle structure. A malfunctioning cuticle layer would not support melanization, regardless of whether the substrates and key enzymes were sufficient and functional, respectively.

We hypothesized that L-DOPA, the reaction product of TH and one of the key chemicals that differ among *A. pisum* individuals, plays an important role in trans-generational regulation. In the present study, we observed the phenotypic changes in response to the changing levels of L-DOPA intake (host plant alternation) and L-DOPA self-synthesis modifications (*APTH*-RNAi). Our results suggest that L-DOPA and dopamine (the neurotransmitter derived from L-DOPA) might participate in the intergenerational signal transmission system. An L-DOPA/dopamine-based developmental signal is transmitted from the mothers to the embryos and determines the future developmental patterns of the nymphs. In this study, a disordered signal induced by *APTH* dsRNA was received by the embryos and caused pathological phenotypic changes thereafter.

The transgenerational signal might not be transmitted via the nervous system (dopamine and its receptors). Studies have shown that physical contact induces wing dimorphism in aphid offspring (Gallot et al., 2010; Vellichirammal et al., 2016), and this stimulation could affect the dopamine pathway (Supplementary Figure S8). However, the results of chemical intervention confirmed that transgenerational signals were not transferred through dopamine receptors and that only the dopamine biosynthesis inhibitor could induce similar phenotypic changes in the daughter aphids. Combined with the essential conditions of the host plant alternations (L-DOPA intake considerably reduced), L-DOPA/dopamine reductions must be the key factor, but dopamine receptor-based neurological regulation is not involved in this transgenerational phenotypic regulation. We suggest the presence of another downstream pathway, which is regulated by dopamine/L-DOPA content, that acts as a transgenerational signal between the mothers and embryos. Further research is required to fully understand this non-neuronal dopamine regulation.

We utilized RNAi and found that it could induce strong phenotypic changes in *A. pisum*. Variable RNAi efficiencies in aphids have previously been reported (Christiaens and Smagghe, 2014; Christiaens et al., 2014; Sapountzis et al., 2014; Singh et al., 2017; Cao et al., 2018). This investigation, however, has demonstrated a successful case for aphid RNAi based on the downregulation results of our target gene.

The aphid phenotypes observed were indicative of the complexity and flexibility of the transgenerational regulations. The present study shows that maternal phenotypic regulation systems of *A. pisum* can be affected in certain conditions, based on the transcriptional modifications. Mother aphids determine the developmental patterns of their daughters, and the dopamine pathway regulation is likely to be involved in cuticular development in daughter aphids via a pathway that does not depend on dopamine receptor-based neurological regulation. L-DOPA, the key chemical in this process, is present in both plants and aphids and functions in the physiological regulations of this process. However, how mother aphids transmit signals to their embryos and how the embryos receive them remains poorly understood. Future studies should focus on determining the genes that may be involved in this process.

## Data Availability Statement

All datasets generated for this study are included in the article/Supplementary Material.

## Author Contributions

YZ and T-XL designed the research. X-XW performed the research. Z-JF, Z-SC, and Z-FZ provided the assistance. YZ and X-XW analyzed the data. and YZ, H-GT, X-XW, and T-XL wrote the manuscript.

## Conflict of Interest

The authors declare that the research was conducted in the absence of any commercial or financial relationships that could be construed as a potential conflict of interest.

## Footnotes

1 http://www.whbioacme.com
