# Supplementary figures and images for: The L-DOPA/Dopamine Pathway Transgenerationally Regulates Cuticular Melanization in the Pea Aphid Acyrthosiphon pisum
Source: Front Cell Dev Biol. 2020 May 5;8:311. doi: 10.3389/fcell.2020.00311 (PMC7214743; doi:10.3389/fcell.2020.00311)

Cluster analysis of differentially expressed genes

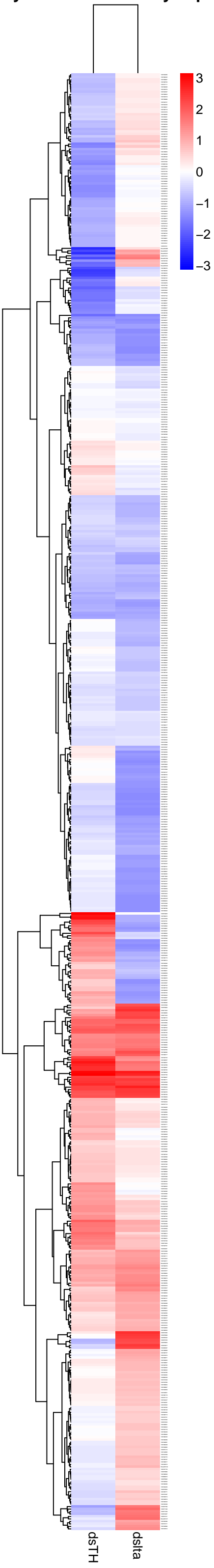

Supplement: Supplementary file 1 [file Data_Sheet_1.ZIP › Transcriptome sequencing/Transcriptome sequencing/heatCluster.detail.pdf]

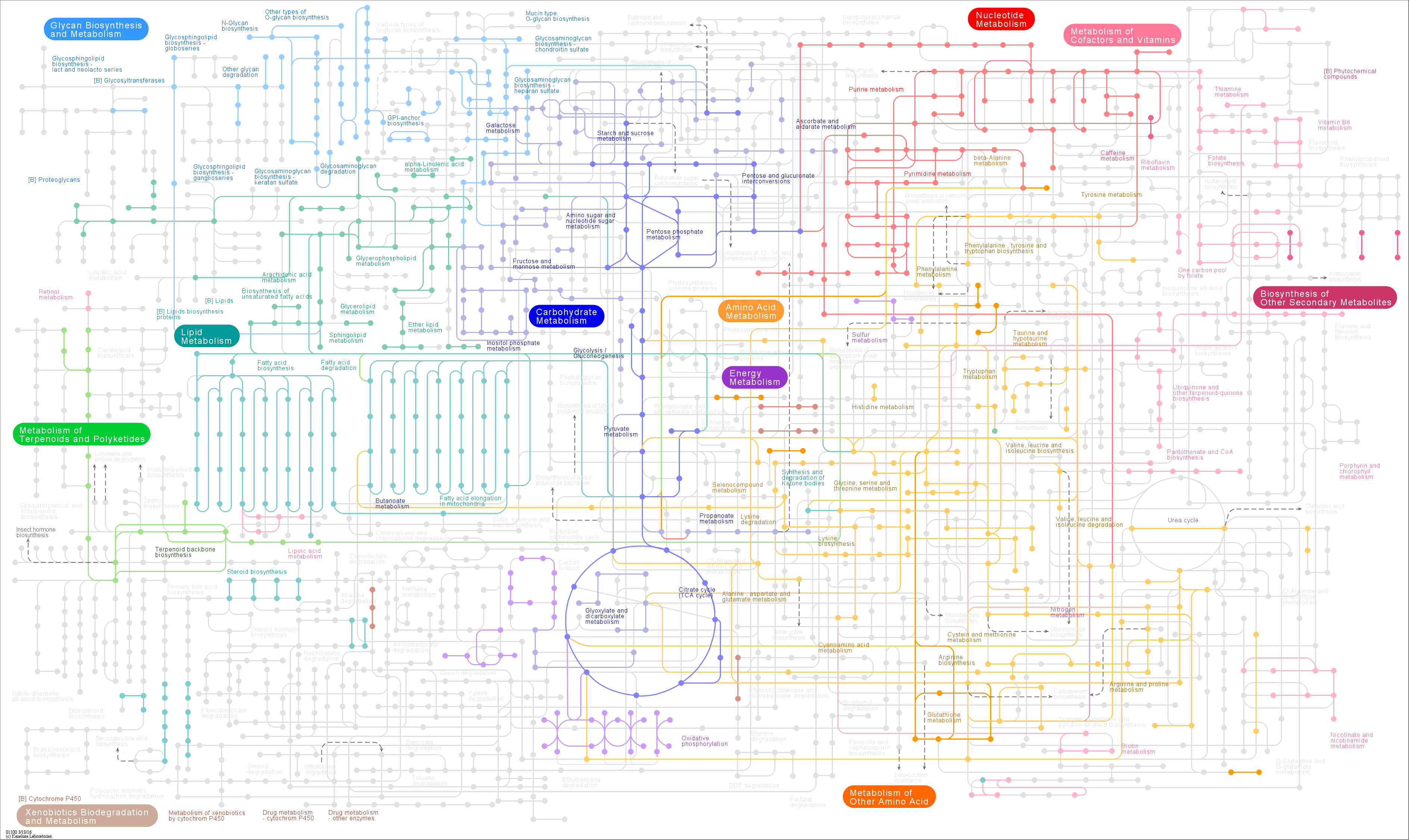

Supplement: Supplementary file 1 [file Data_Sheet_1.ZIP › Transcriptome sequencing/Transcriptome sequencing/KEGG/KEGG_pathway.png]

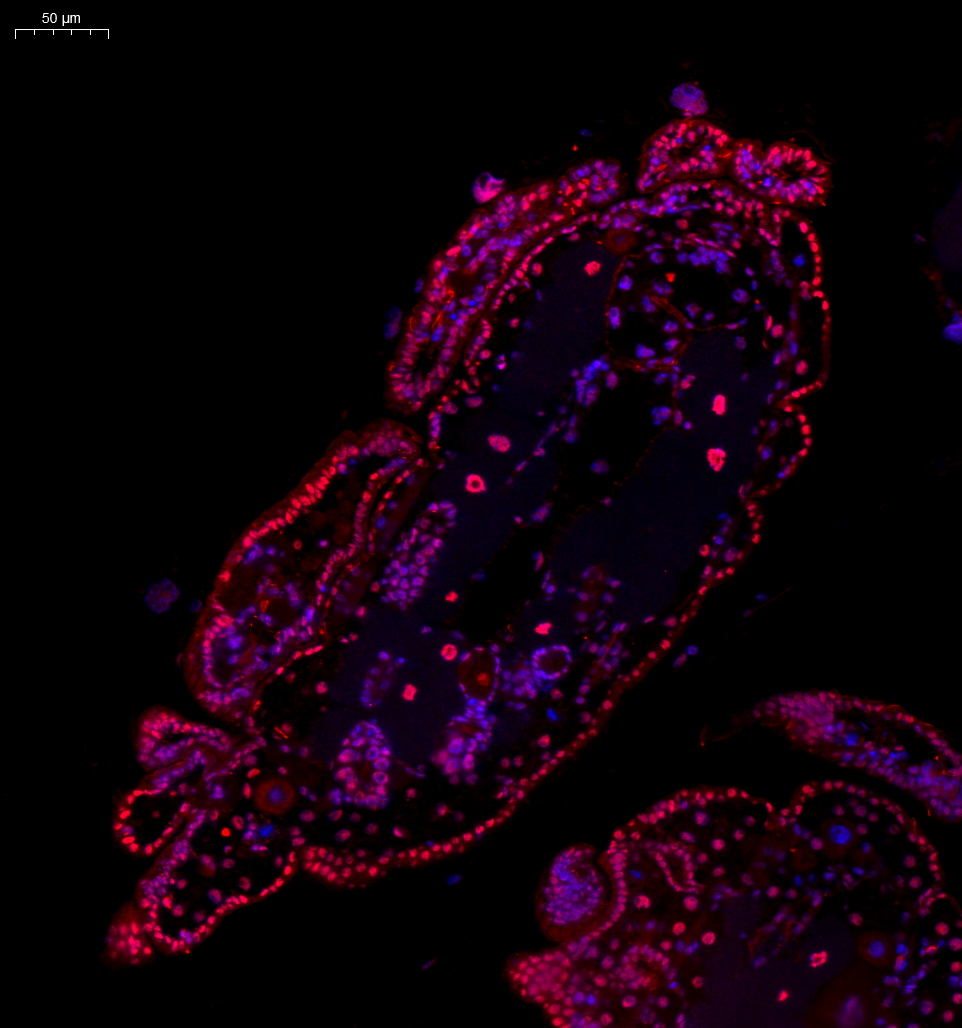

Supplement: Supplementary file 2 [file Data_Sheet_2.ZIP › daughters/CK-lta/T1 IF TH红_23.4x-1.jpg]

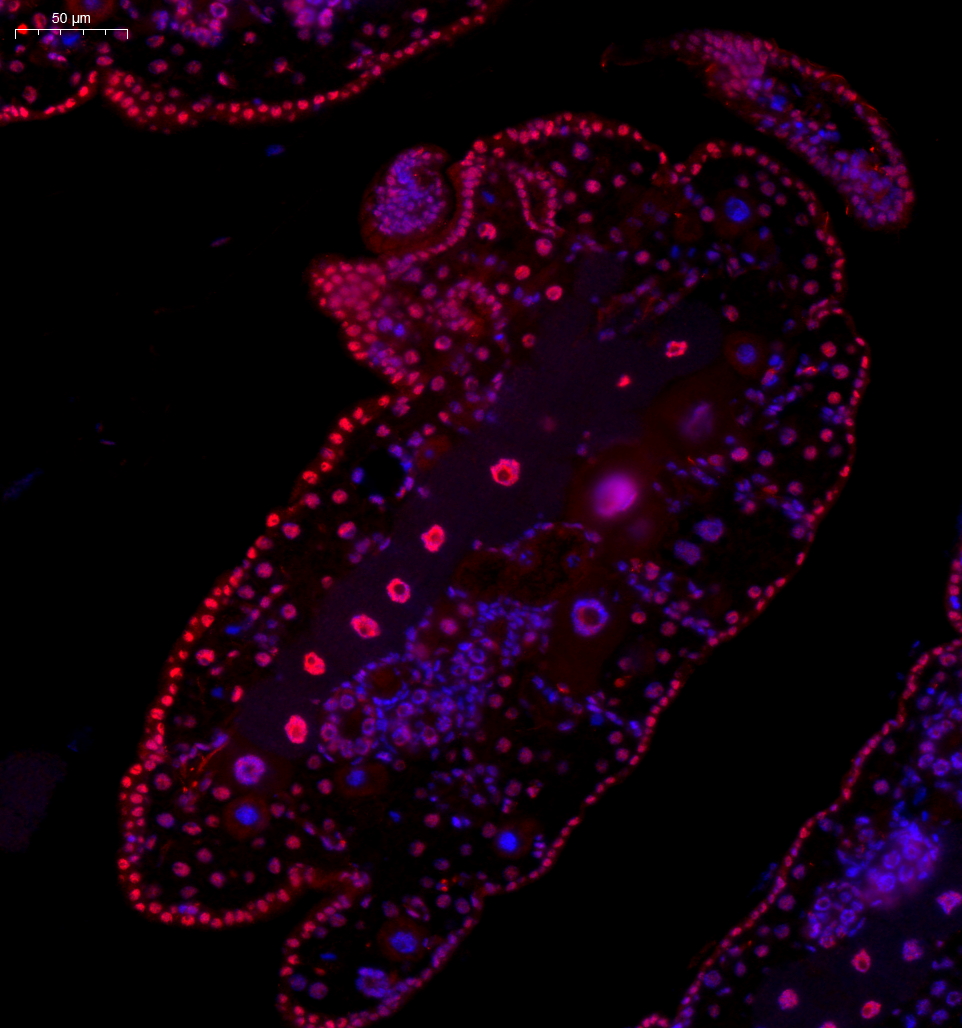

Supplement: Supplementary file 2 [file Data_Sheet_2.ZIP › daughters/CK-lta/T1 IF TH红_28.1x-2.jpg]

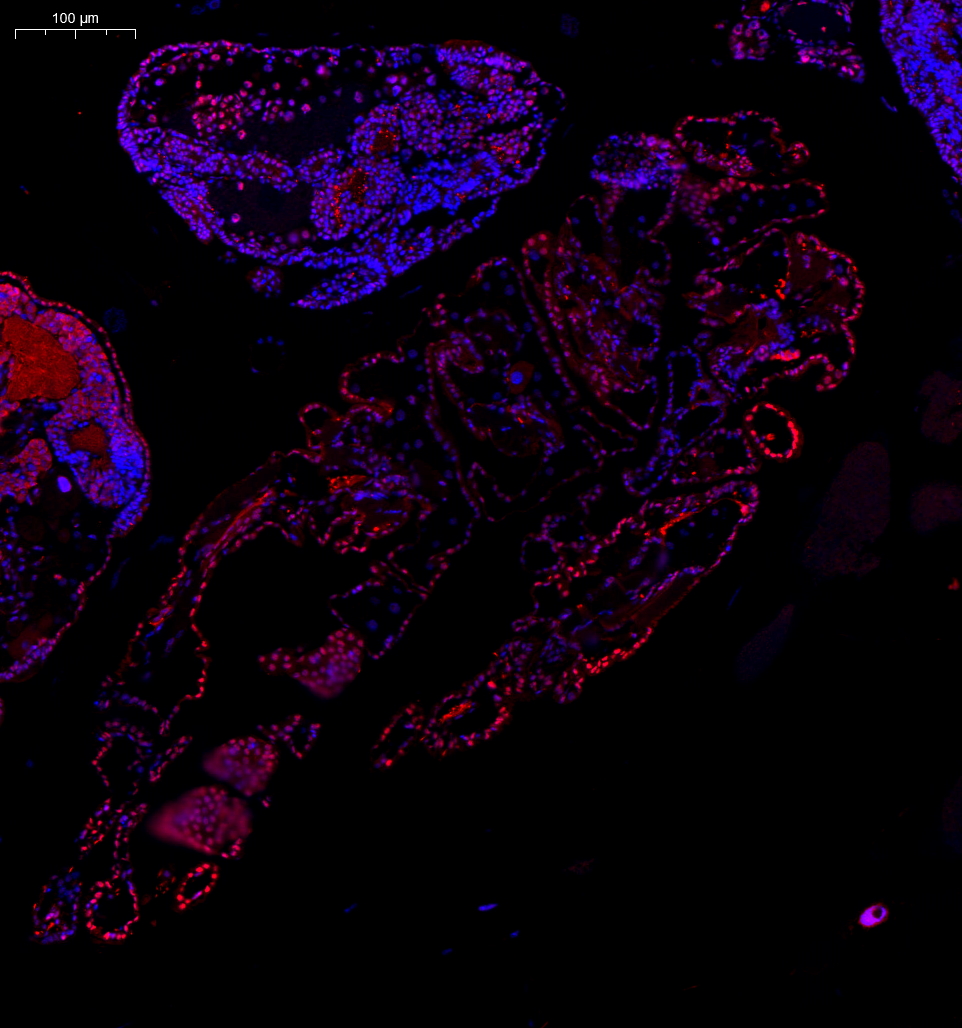

Supplement: Supplementary file 2 [file Data_Sheet_2.ZIP › daughters/CK-lta/T2 IF TH红_15.1x-4.jpg]

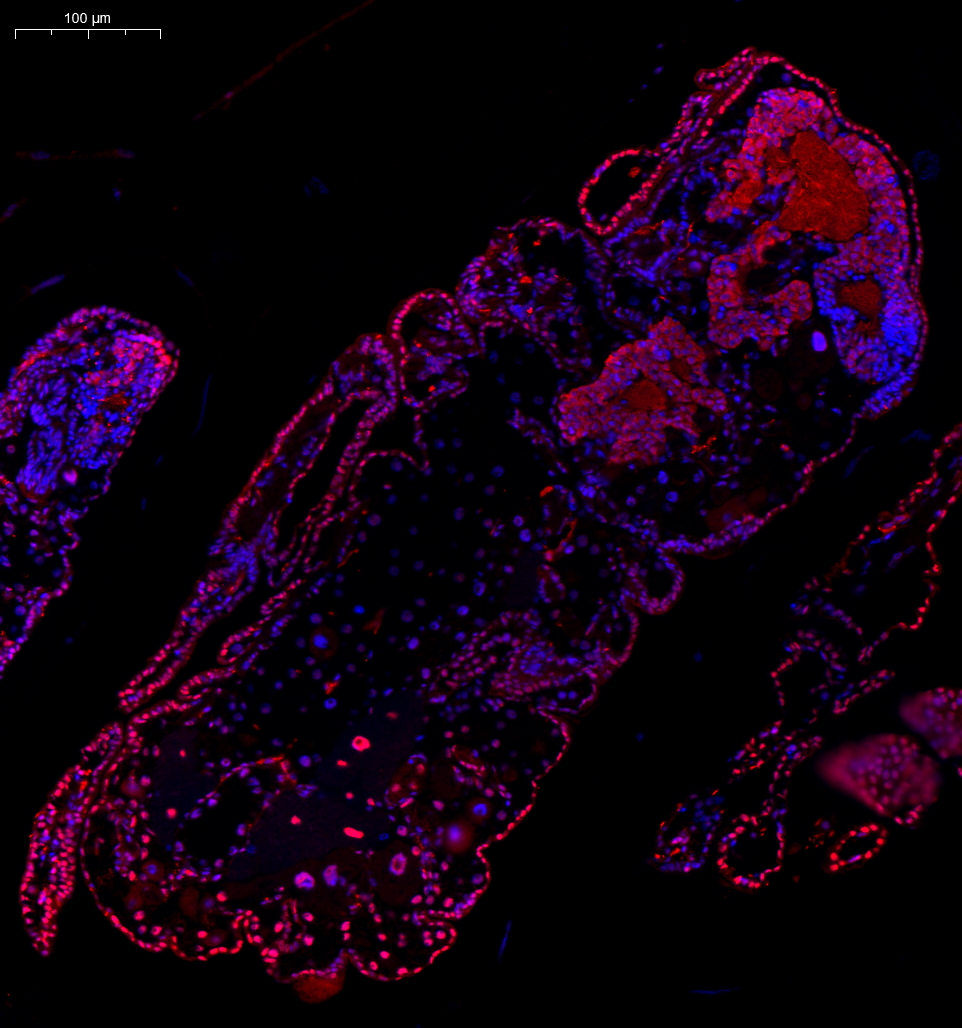

Supplement: Supplementary file 2 [file Data_Sheet_2.ZIP › daughters/CK-lta/T2 IF TH红_18.1x-5.jpg]

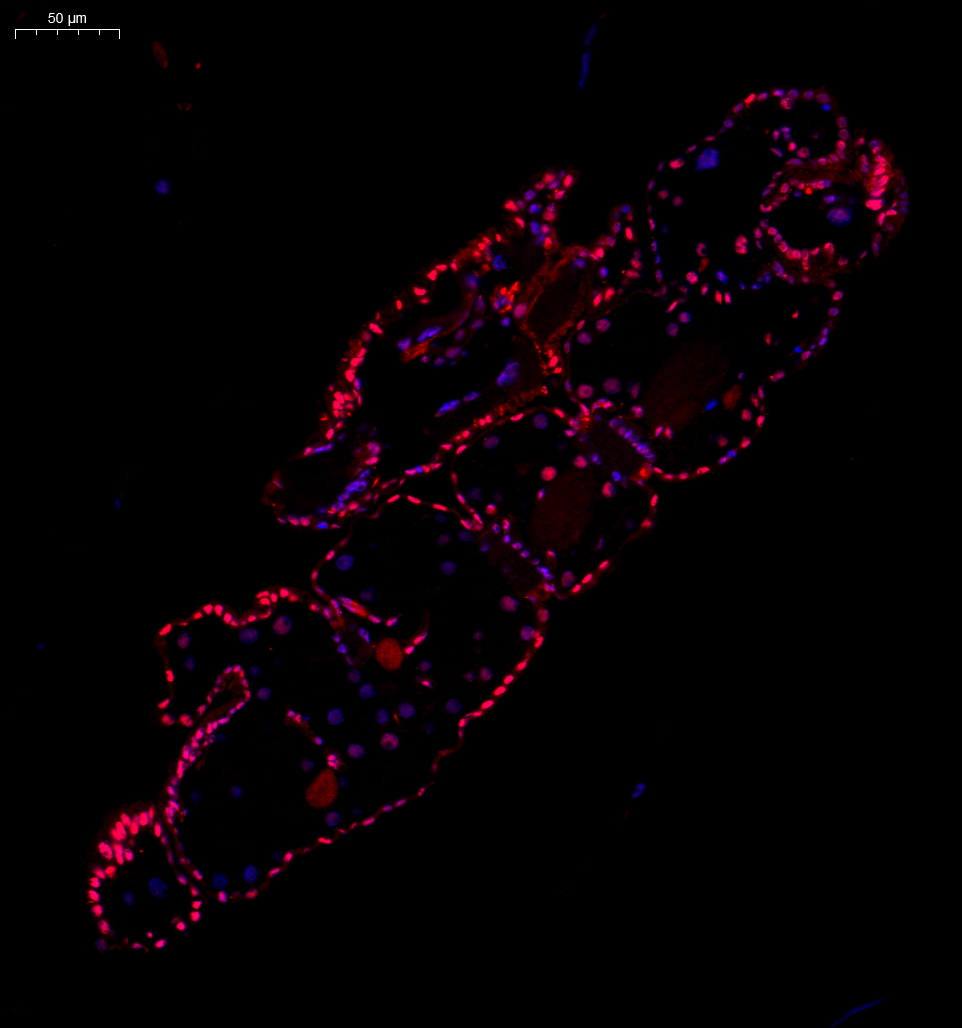

Supplement: Supplementary file 2 [file Data_Sheet_2.ZIP › daughters/CK-lta/T2 IF TH红_26.0x-6.jpg]

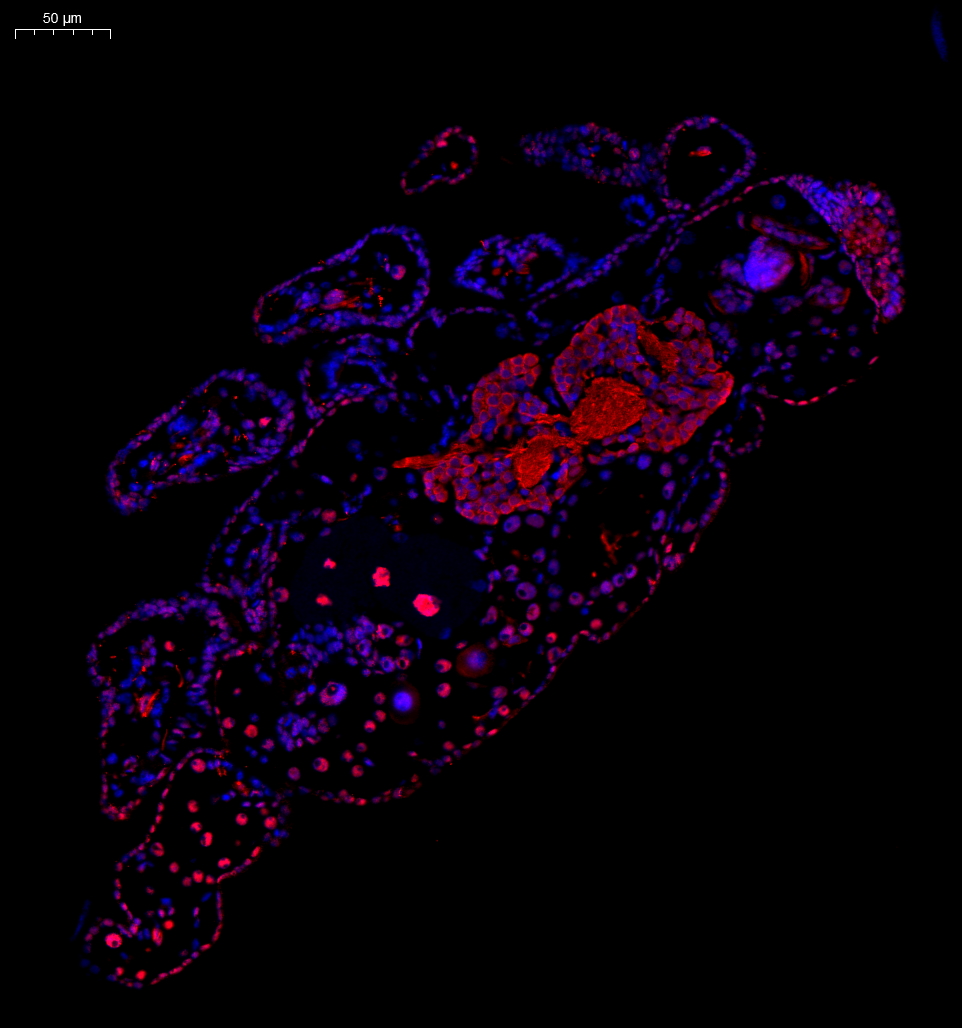

Supplement: Supplementary file 2 [file Data_Sheet_2.ZIP › daughters/CK-lta/T3 IF TH红_23.8x-7.jpg]

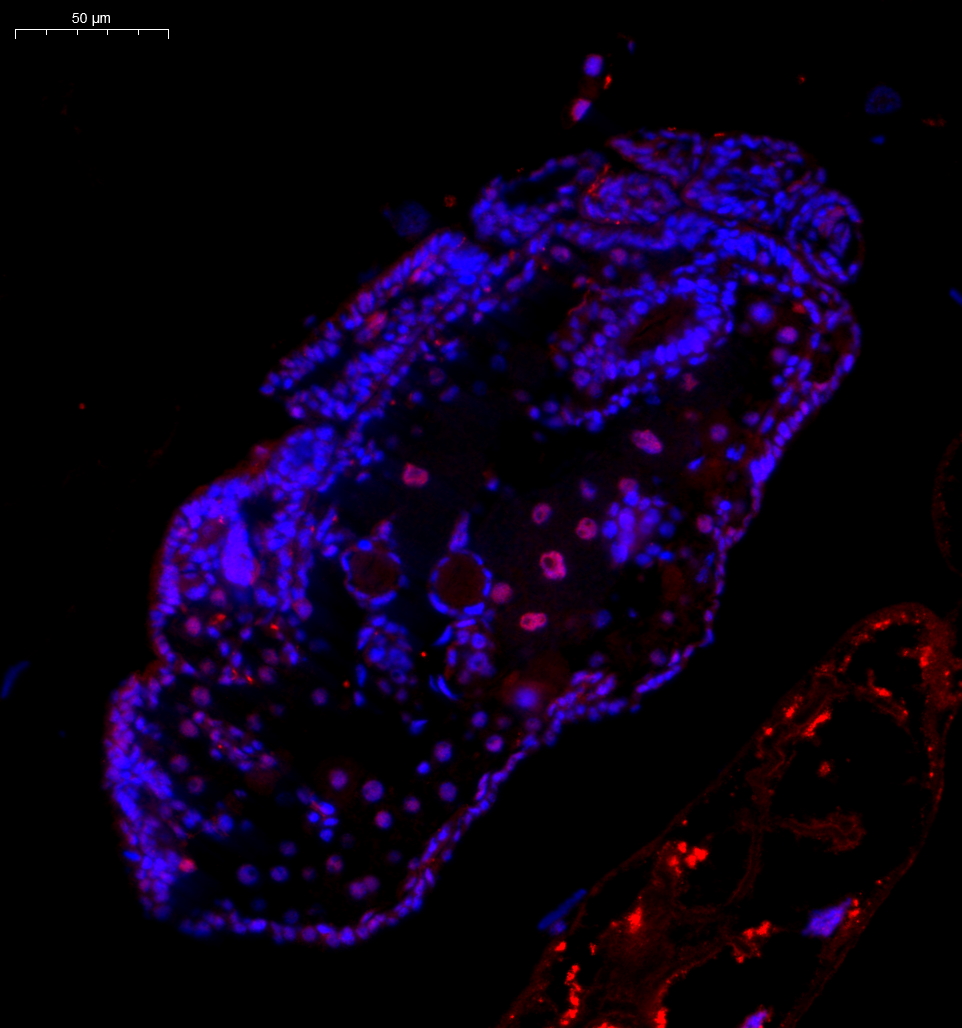

Supplement: Supplementary file 2 [file Data_Sheet_2.ZIP › daughters/CK-lta/V3 IF TH红_38.1x-8.jpg]

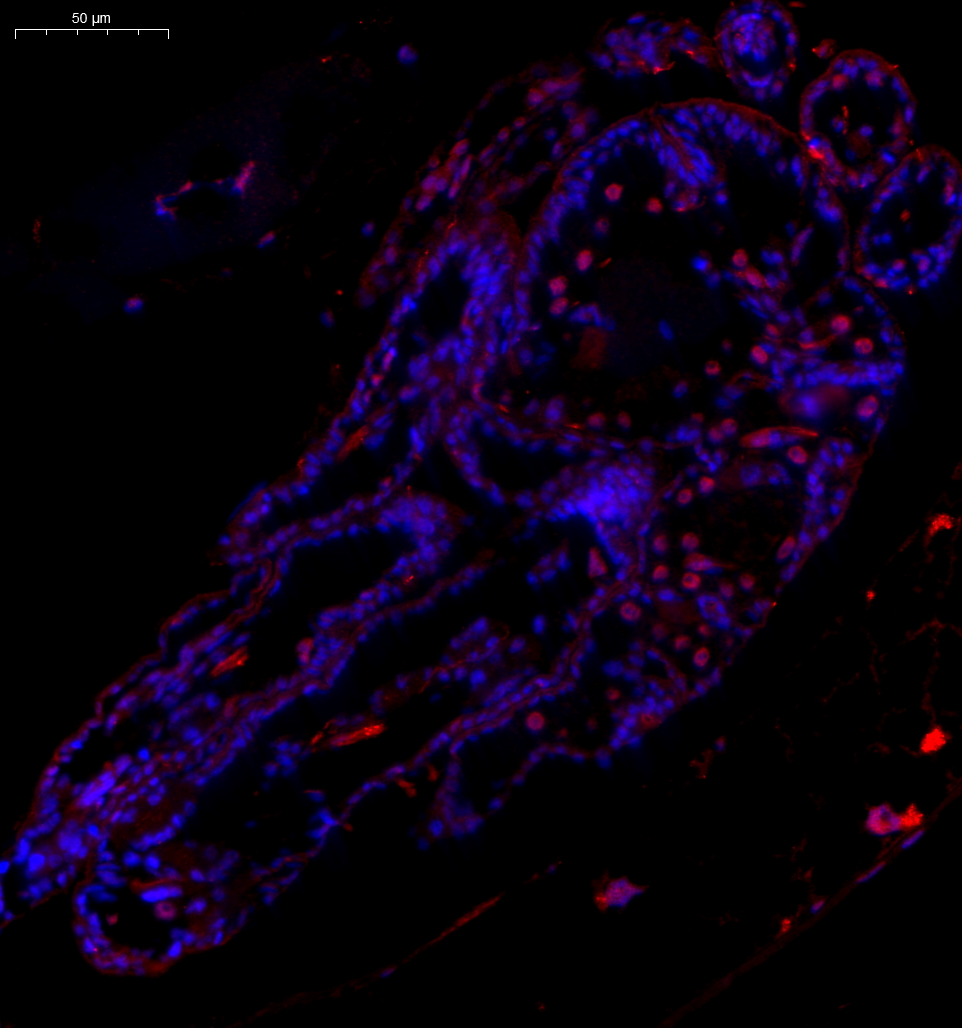

Supplement: Supplementary file 2 [file Data_Sheet_2.ZIP › daughters/CK-lta/V3 IF TH红_38.1x-9.jpg]

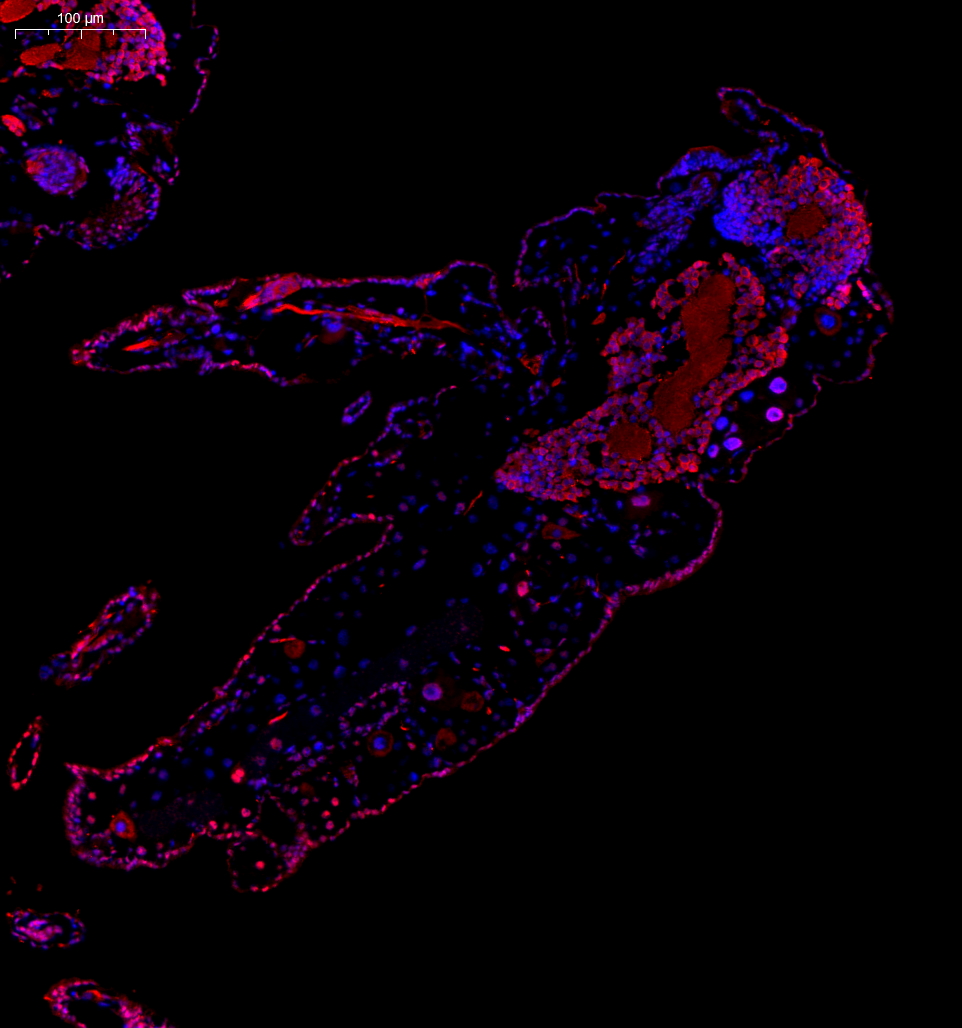

Supplement: Supplementary file 2 [file Data_Sheet_2.ZIP › daughters/CK-lta/V4 IF TH红_16.3x-10.jpg]

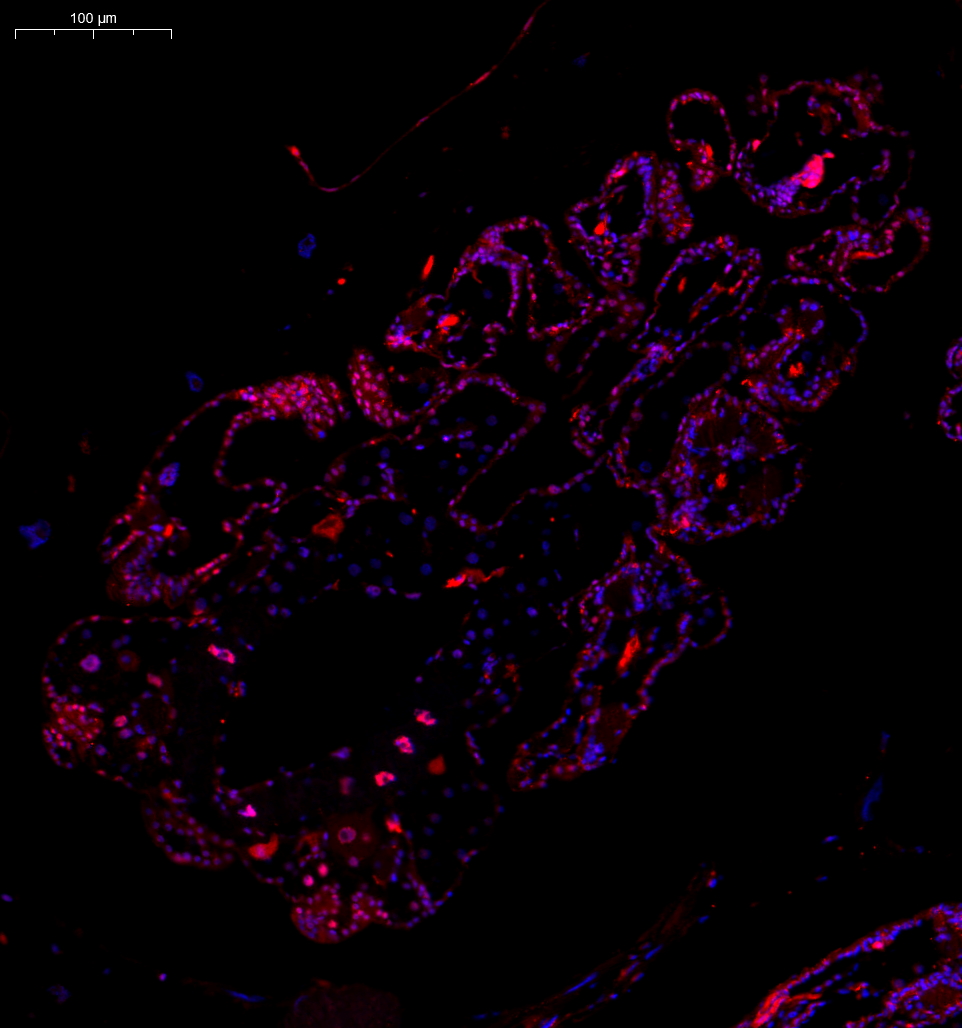

Supplement: Supplementary file 2 [file Data_Sheet_2.ZIP › daughters/CK-lta/V5 IF TH红_19.5x-13.jpg]

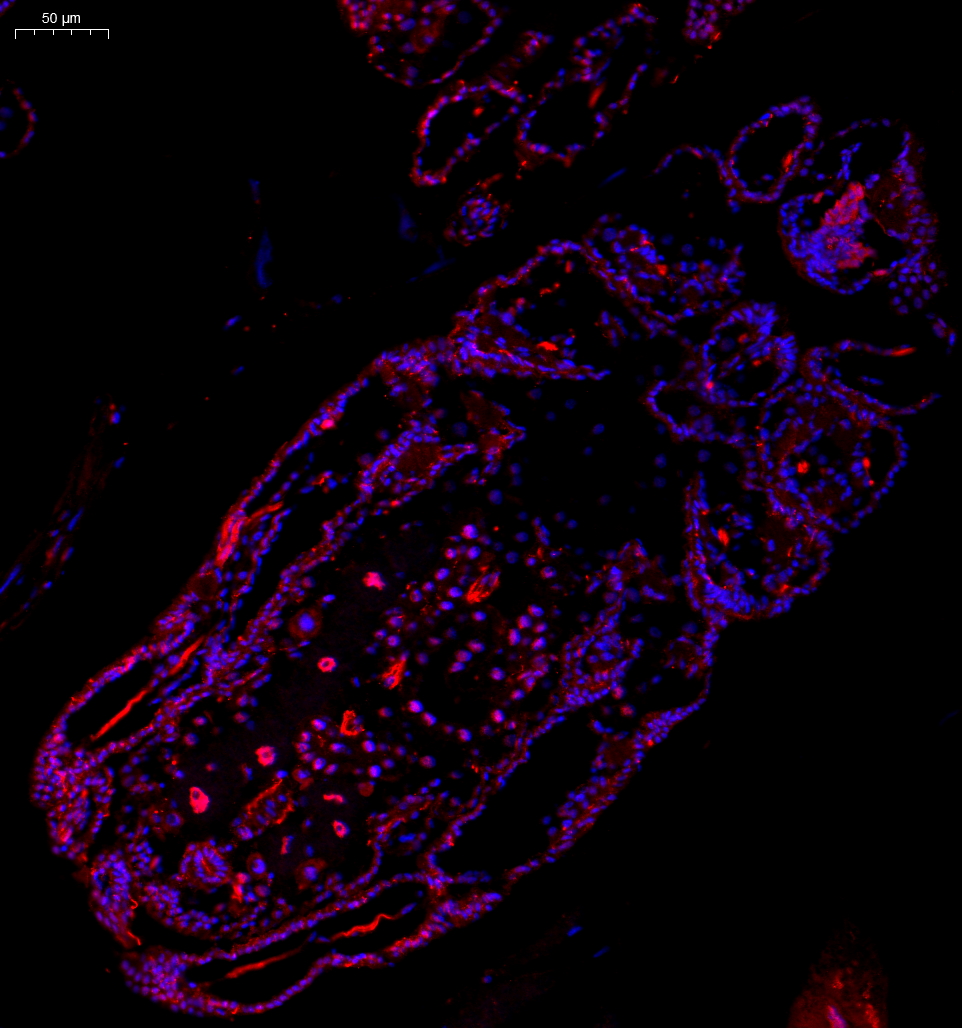

Supplement: Supplementary file 2 [file Data_Sheet_2.ZIP › daughters/CK-lta/V5 IF TH红_23.4x-11.jpg]

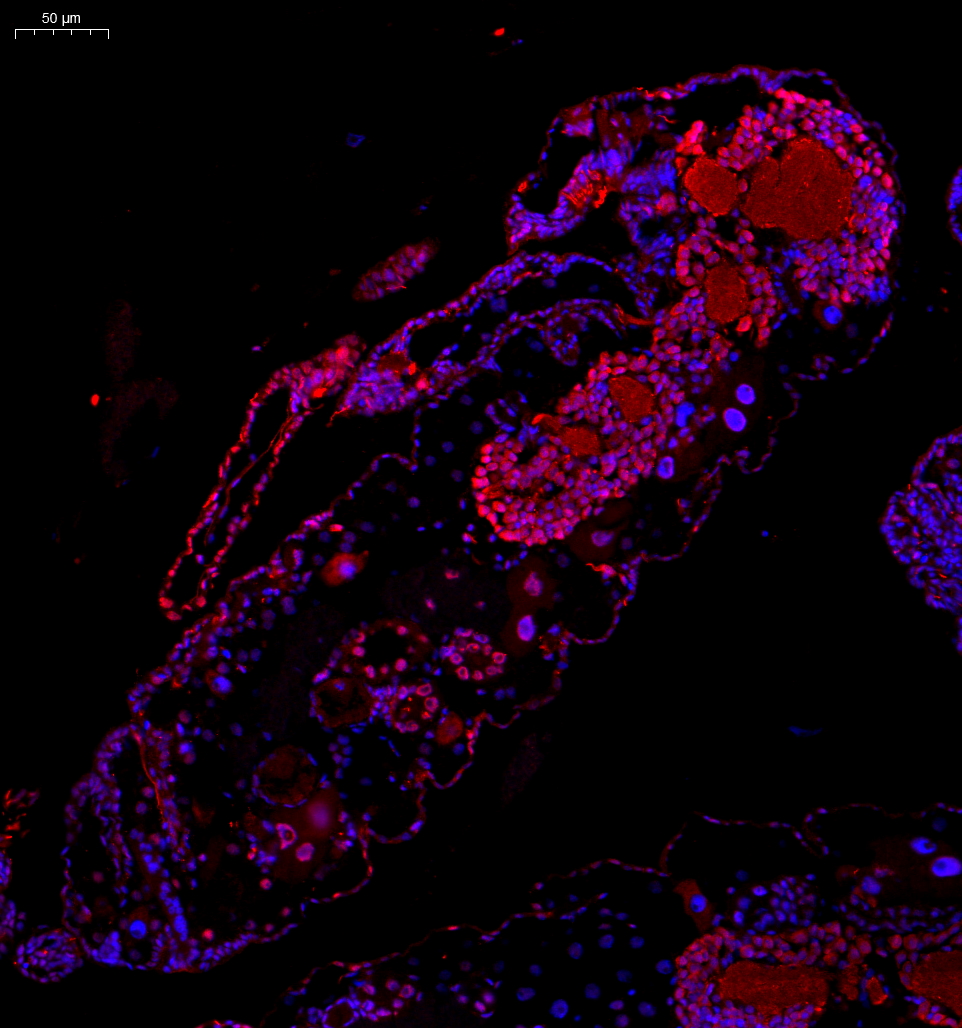

Supplement: Supplementary file 2 [file Data_Sheet_2.ZIP › daughters/CK-lta/V5 IF TH红_23.4x-12.jpg]

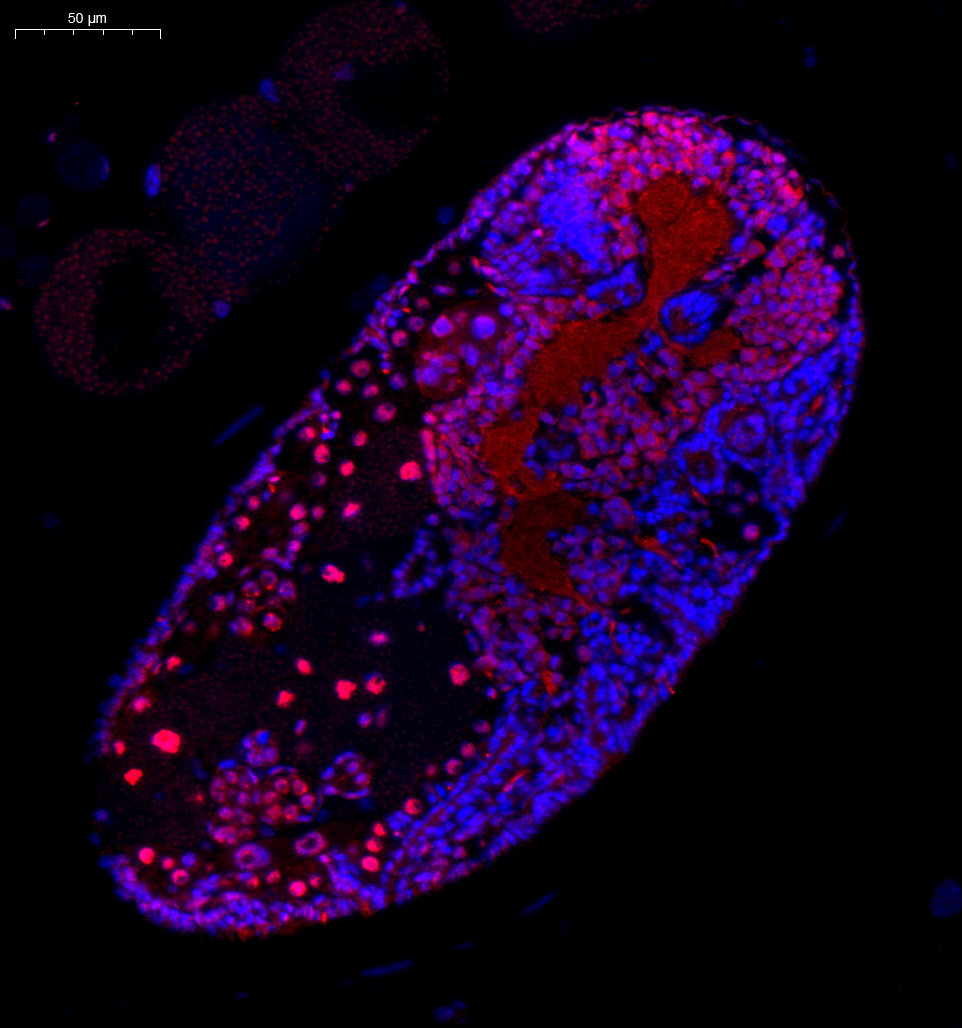

Supplement: Supplementary file 2 [file Data_Sheet_2.ZIP › daughters/CK-lta/VW2 IF TH红_36.2x-14.jpg]

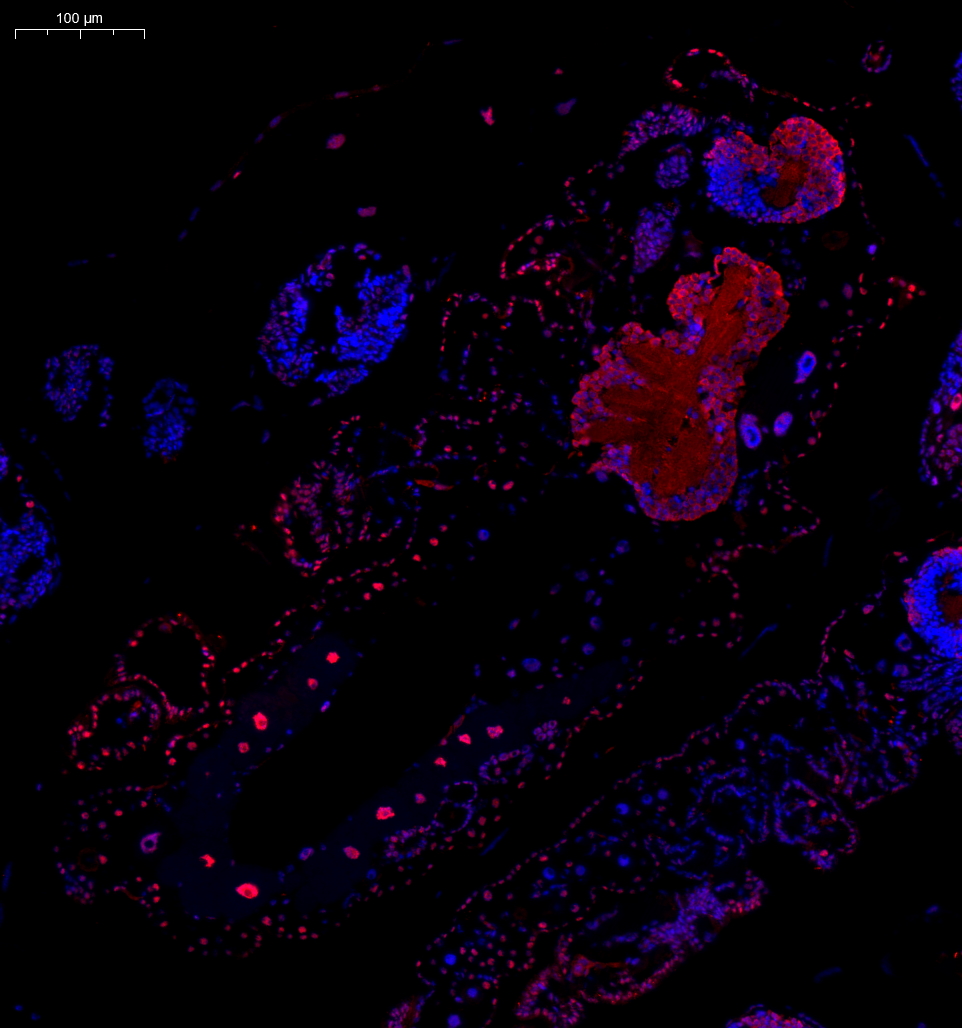

Supplement: Supplementary file 2 [file Data_Sheet_2.ZIP › daughters/TH/R1 IF TH红_16.1x-2.jpg]

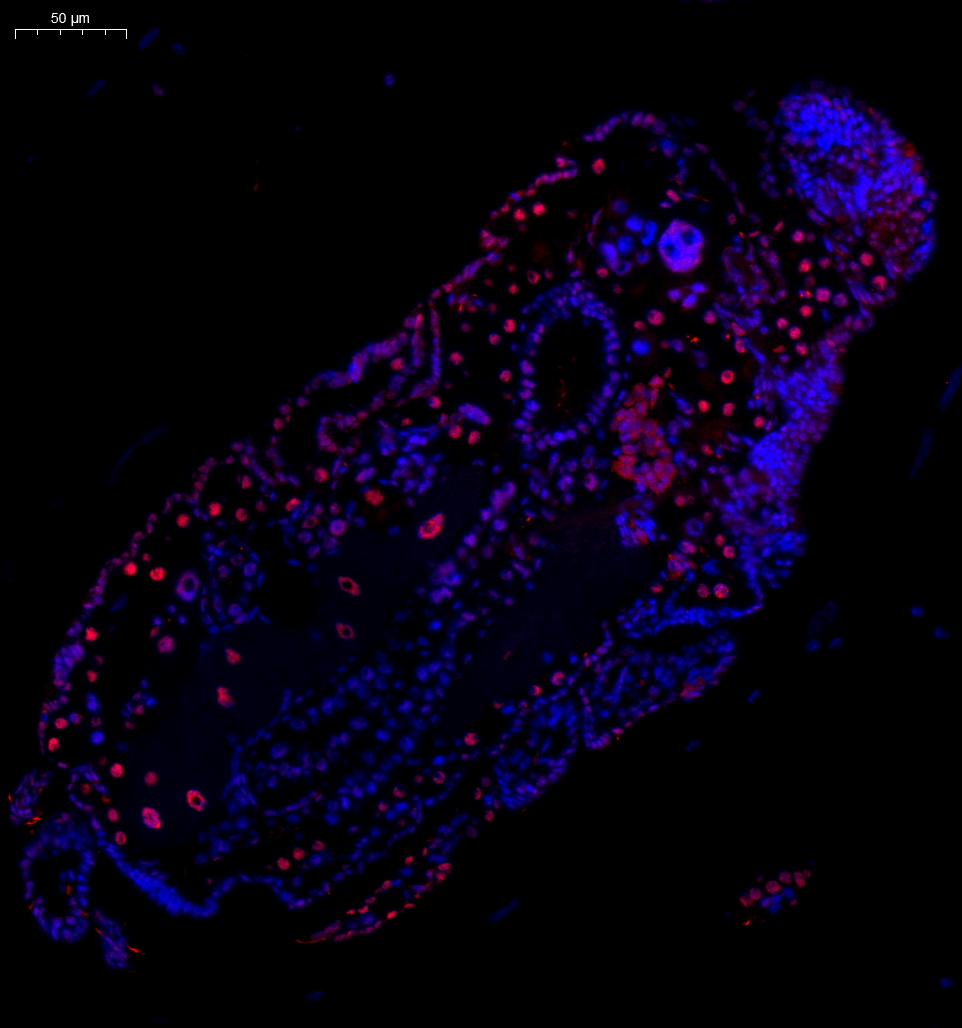

Supplement: Supplementary file 2 [file Data_Sheet_2.ZIP › daughters/TH/R1 IF TH红_27.8x-1.jpg]

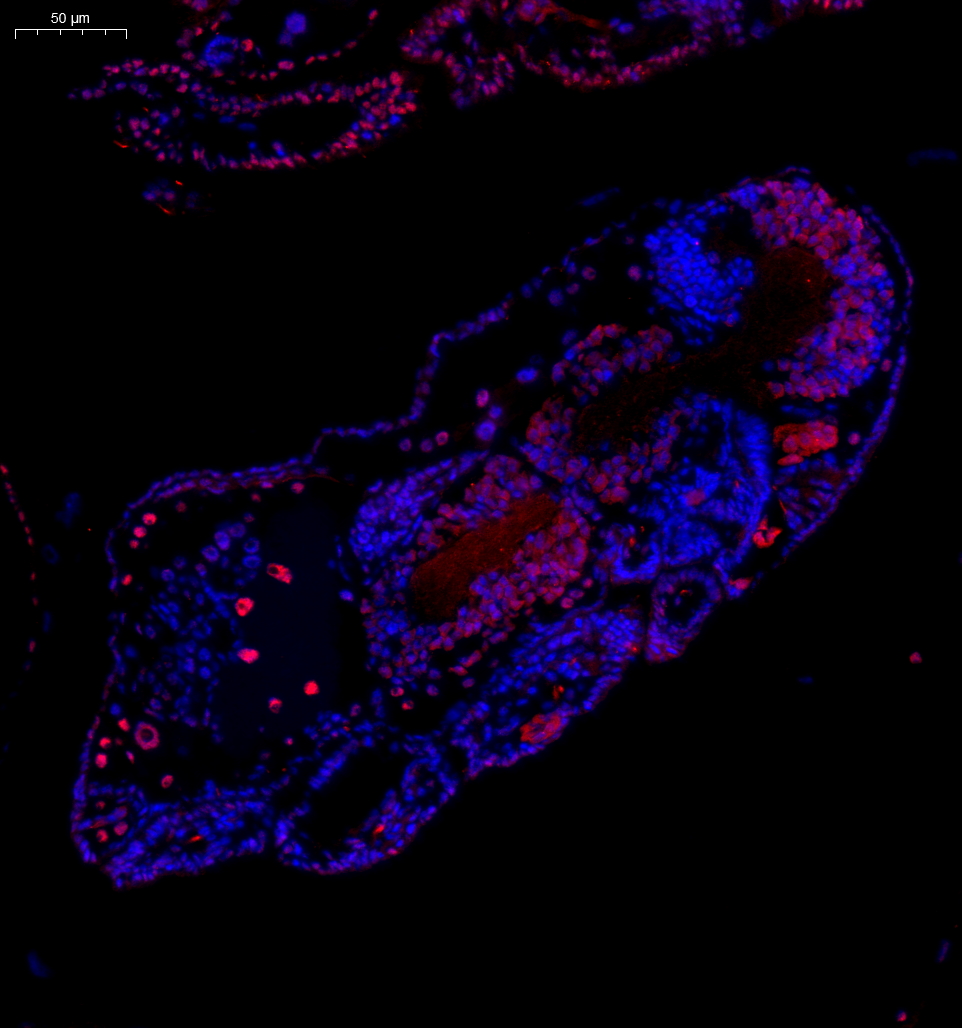

Supplement: Supplementary file 2 [file Data_Sheet_2.ZIP › daughters/TH/R1 IF TH红_27.8x-3.jpg]

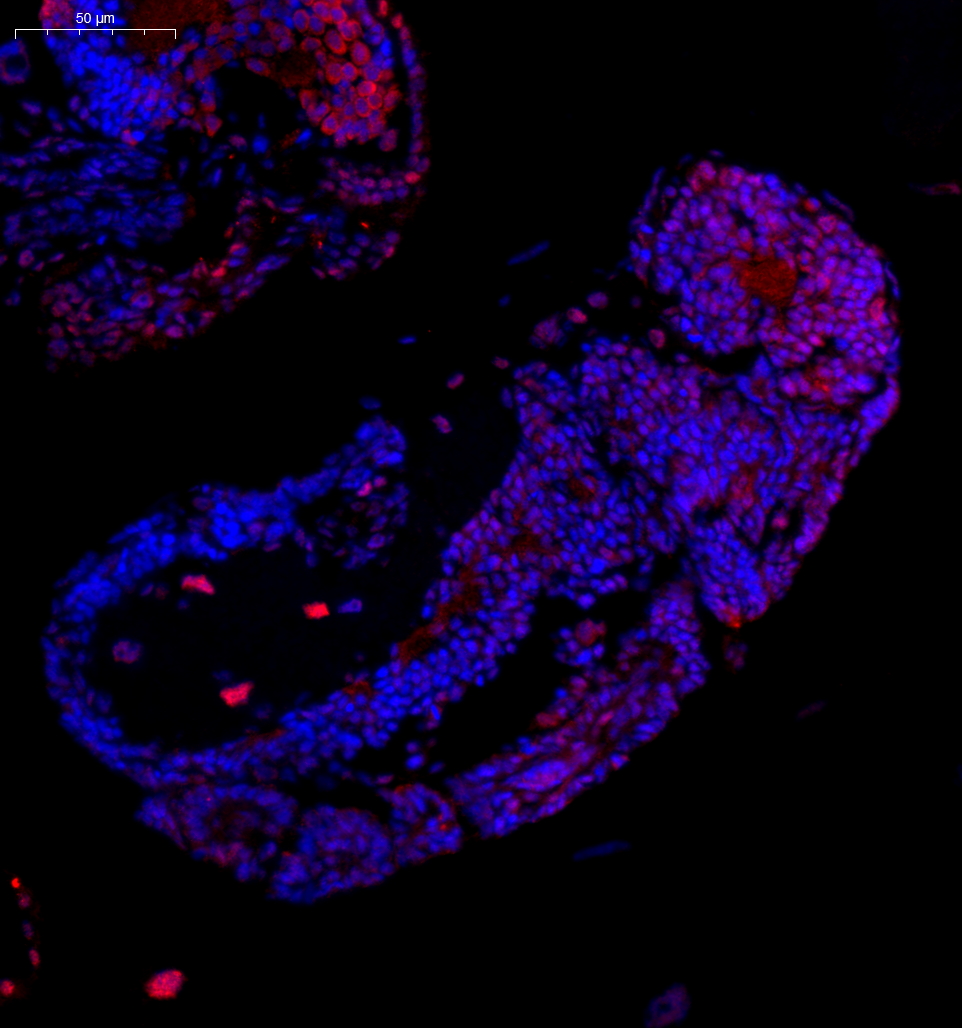

Supplement: Supplementary file 2 [file Data_Sheet_2.ZIP › daughters/TH/R1 IF TH红_40.0x-4.jpg]

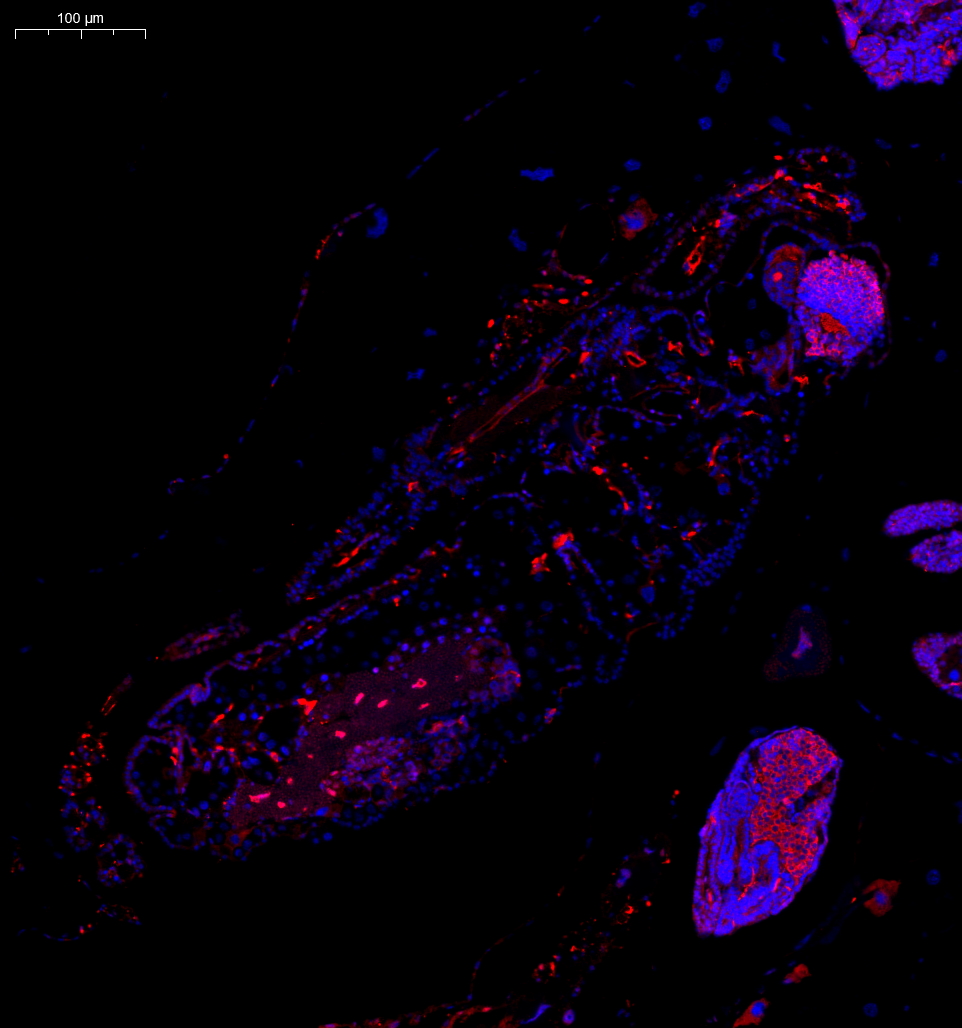

Supplement: Supplementary file 2 [file Data_Sheet_2.ZIP › daughters/TH/R2 IF TH红_16.3x-5.jpg]

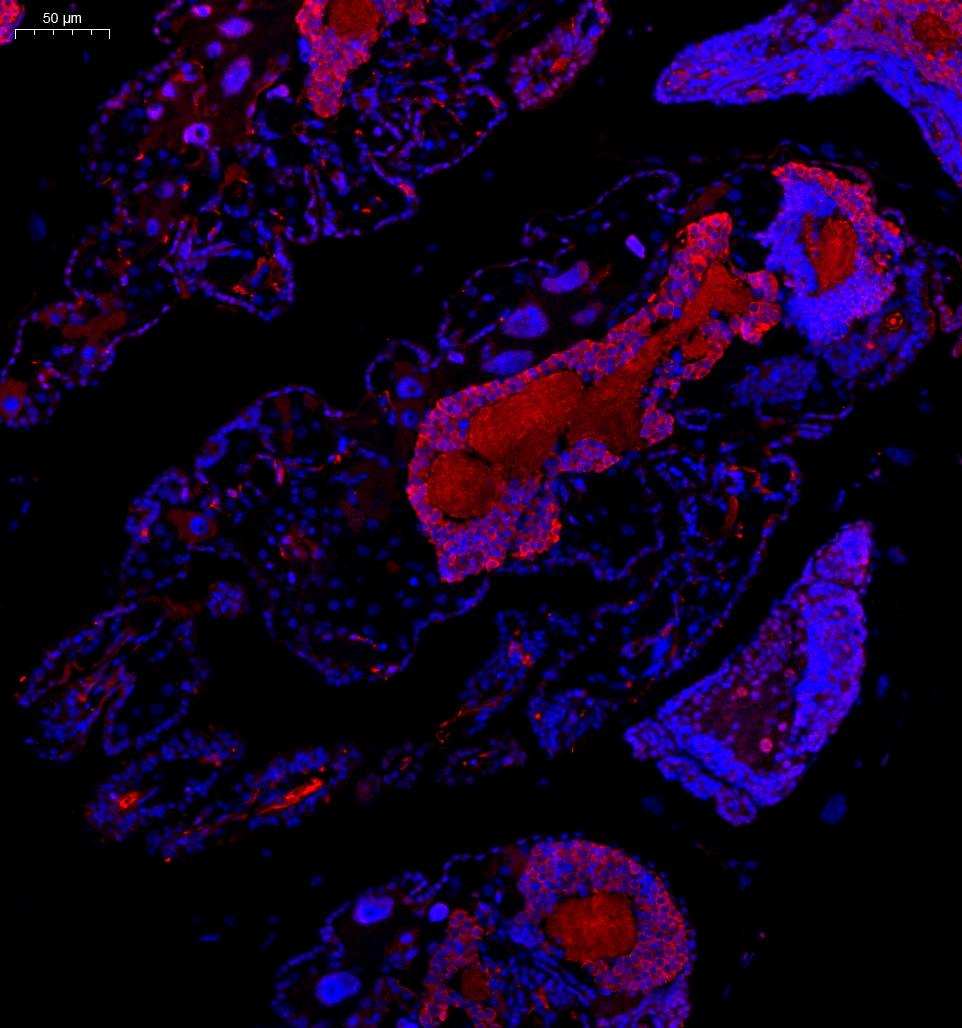

Supplement: Supplementary file 2 [file Data_Sheet_2.ZIP › daughters/TH/R2 IF TH红_23.4x-7.jpg]

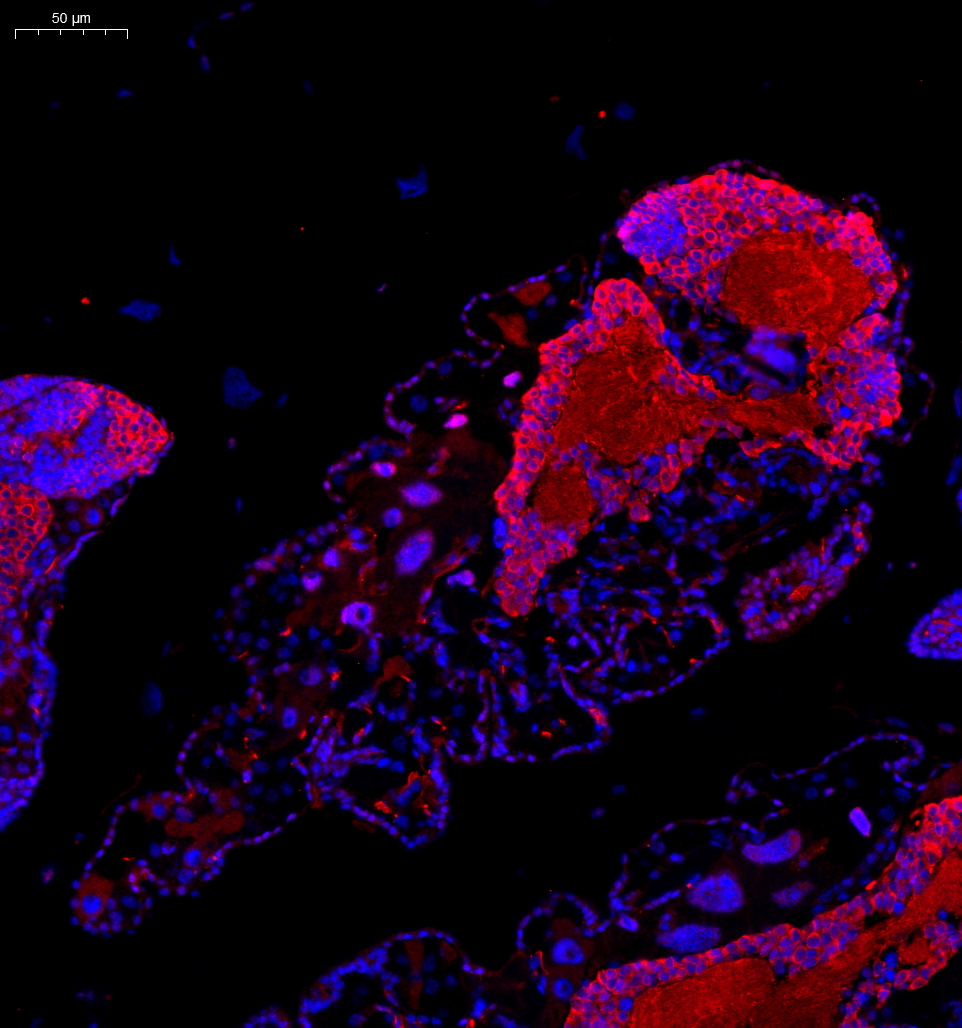

Supplement: Supplementary file 2 [file Data_Sheet_2.ZIP › daughters/TH/R2 IF TH红_28.1x-6.jpg]

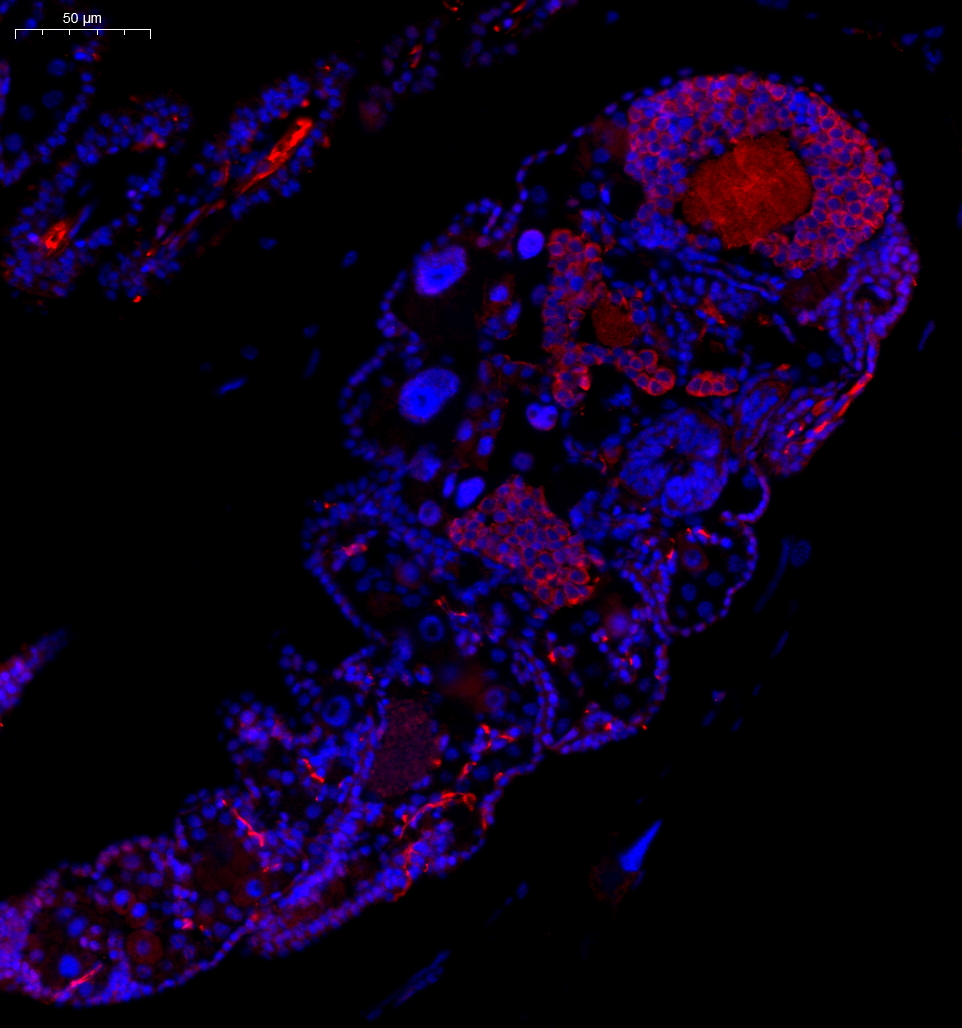

Supplement: Supplementary file 2 [file Data_Sheet_2.ZIP › daughters/TH/R2 IF TH红_33.7x-8.jpg]

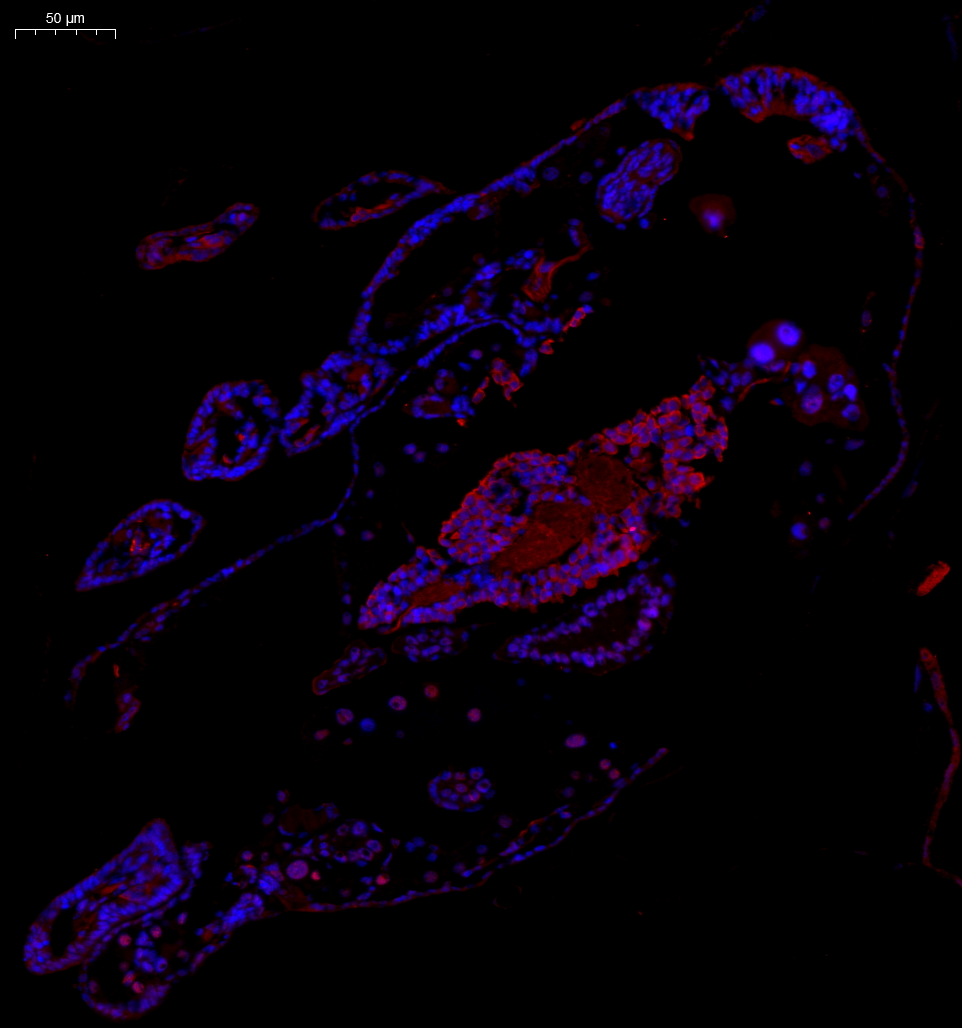

Supplement: Supplementary file 2 [file Data_Sheet_2.ZIP › daughters/TH/R3 IF TH红_25.1x-11.jpg]

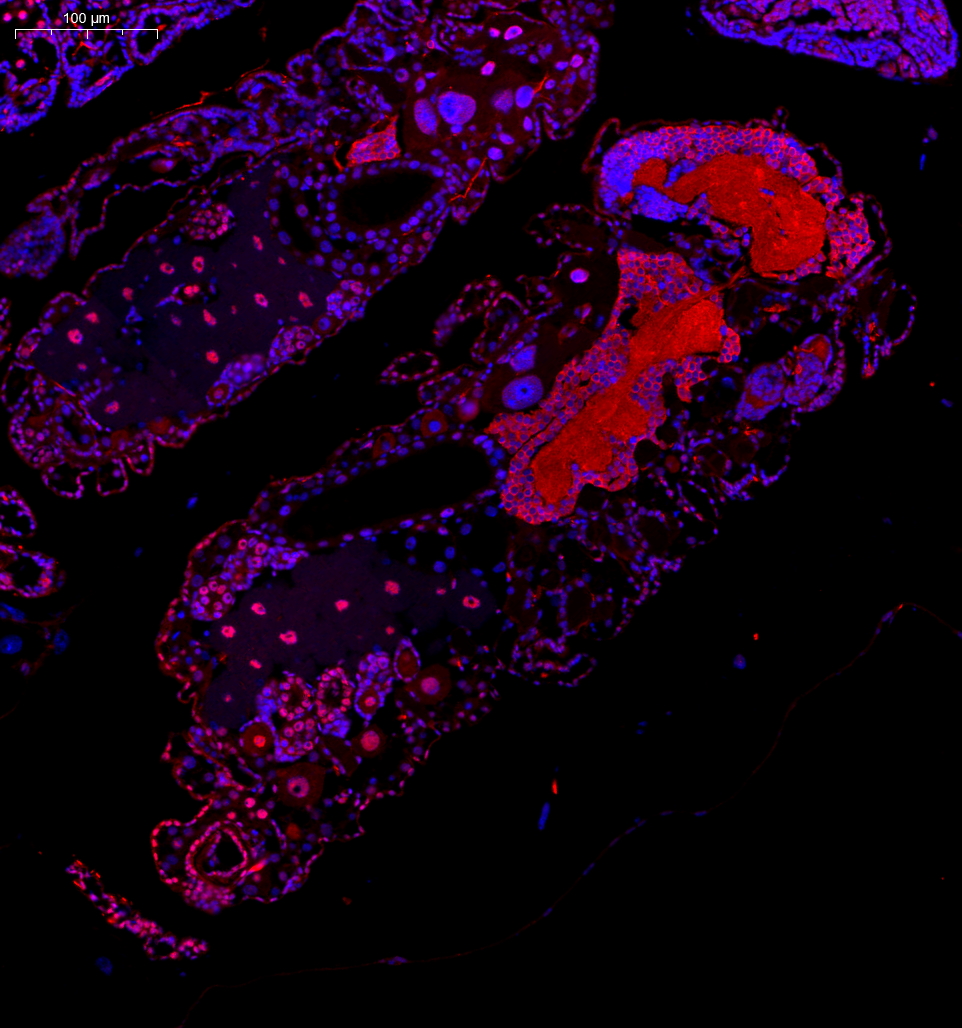

Supplement: Supplementary file 2 [file Data_Sheet_2.ZIP › daughters/TH/R4 IF TH红_17.8x-9.jpg]

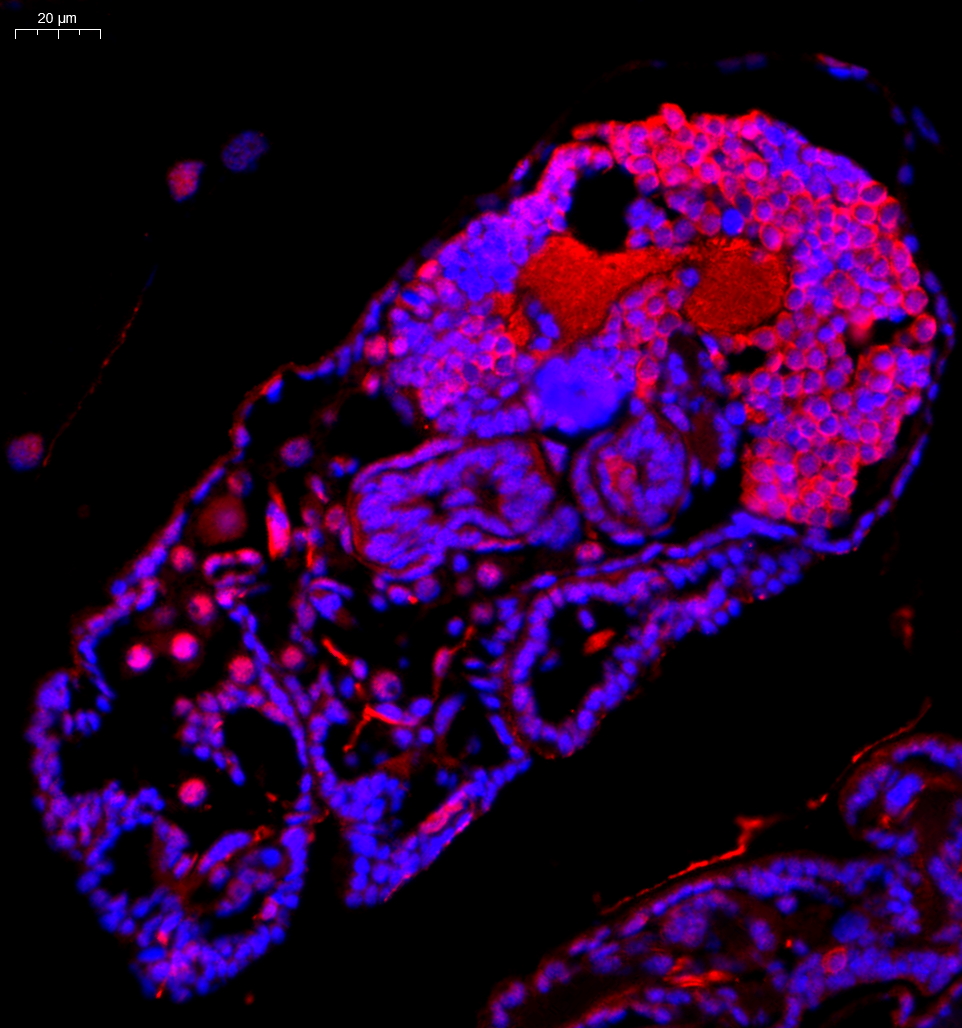

Supplement: Supplementary file 2 [file Data_Sheet_2.ZIP › daughters/TH/R4 IF TH红_53.1x-10.jpg]

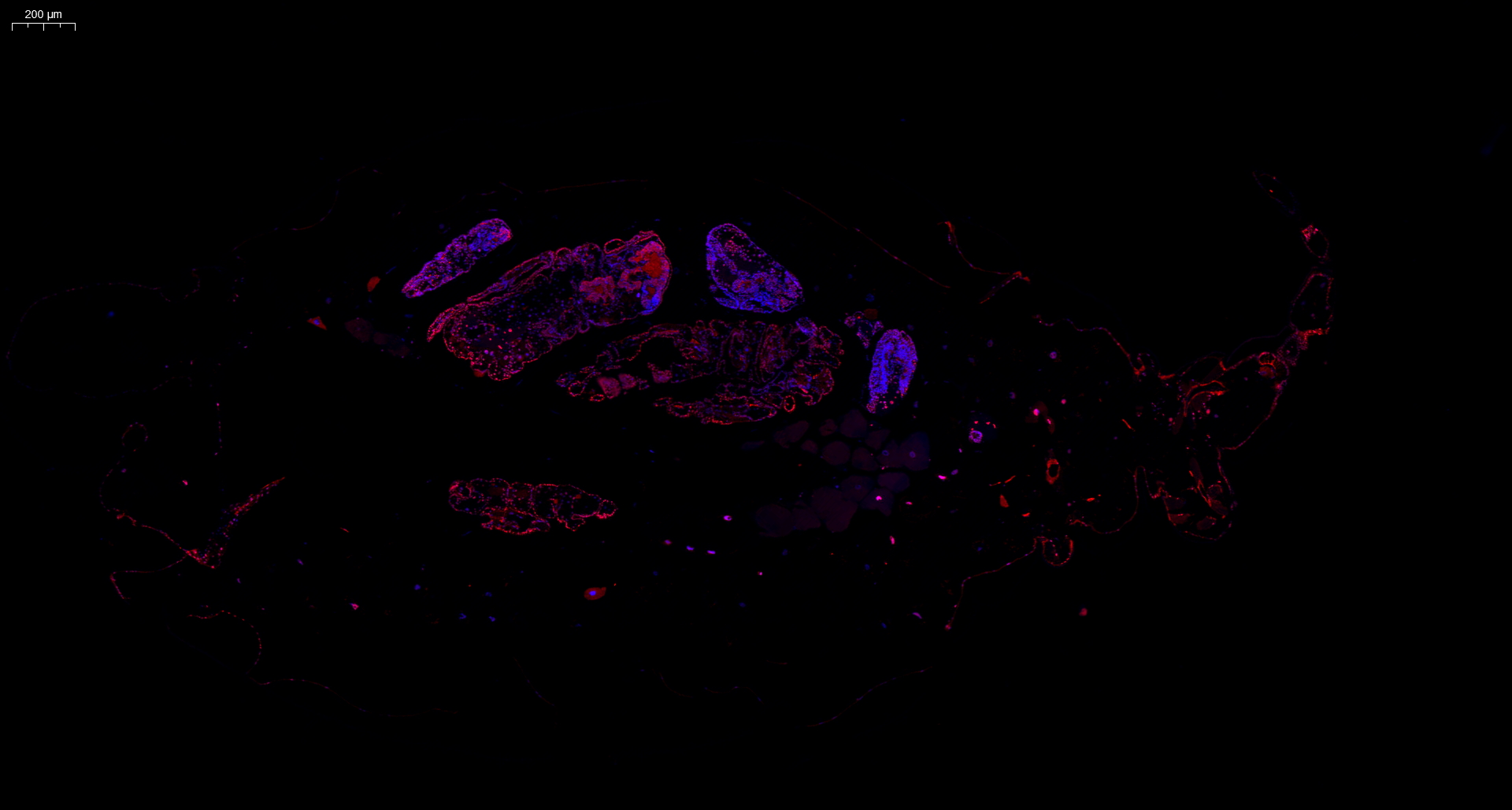

Supplement: Supplementary file 2 [file Data_Sheet_2.ZIP › mothers/T.repens-ck_5.0x-1.jpg]

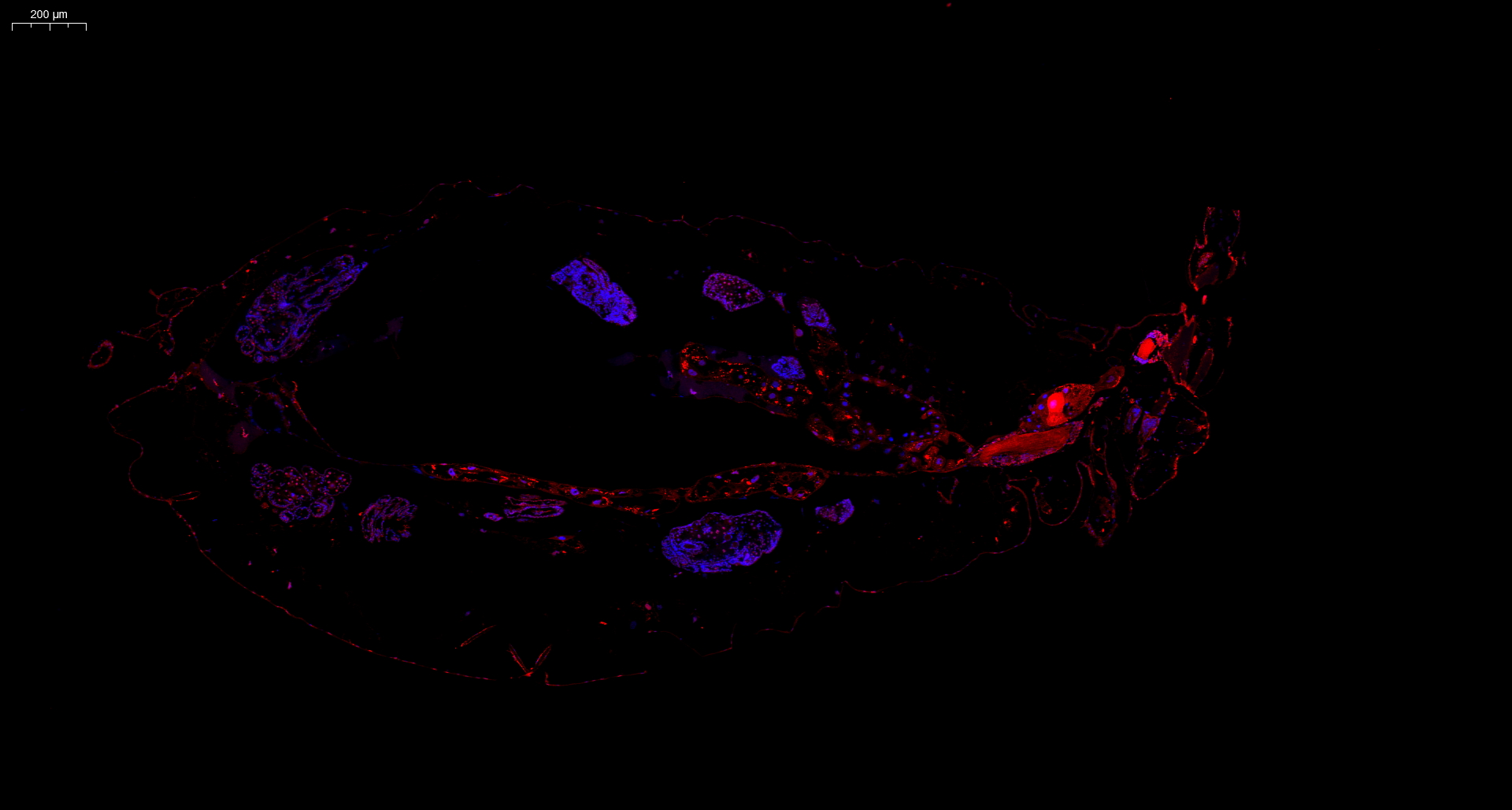

Supplement: Supplementary file 2 [file Data_Sheet_2.ZIP › mothers/T.repens-ck_5.9x-4.jpg]

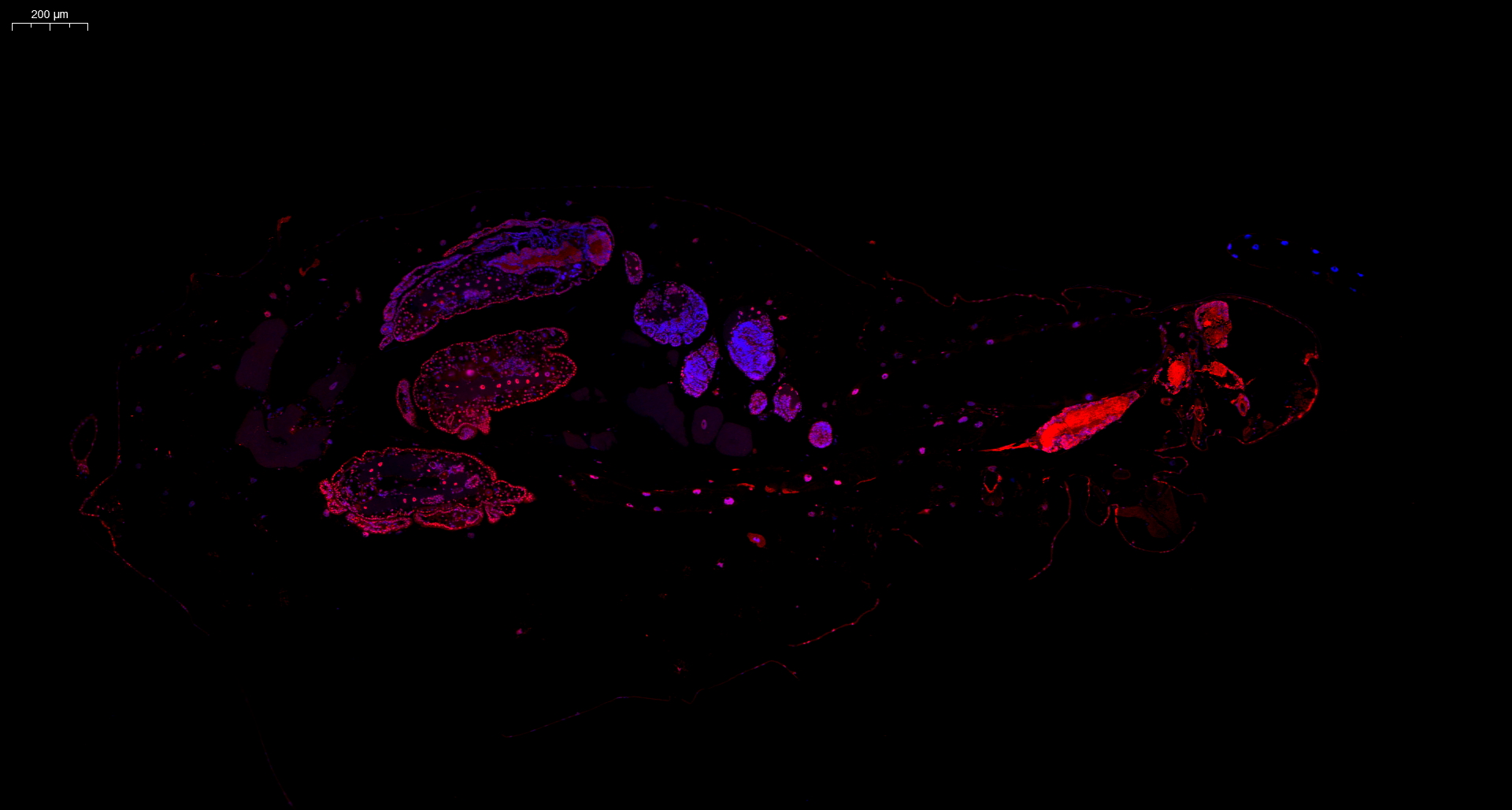

Supplement: Supplementary file 2 [file Data_Sheet_2.ZIP › mothers/T.repens-ck_6.0x-2.jpg]

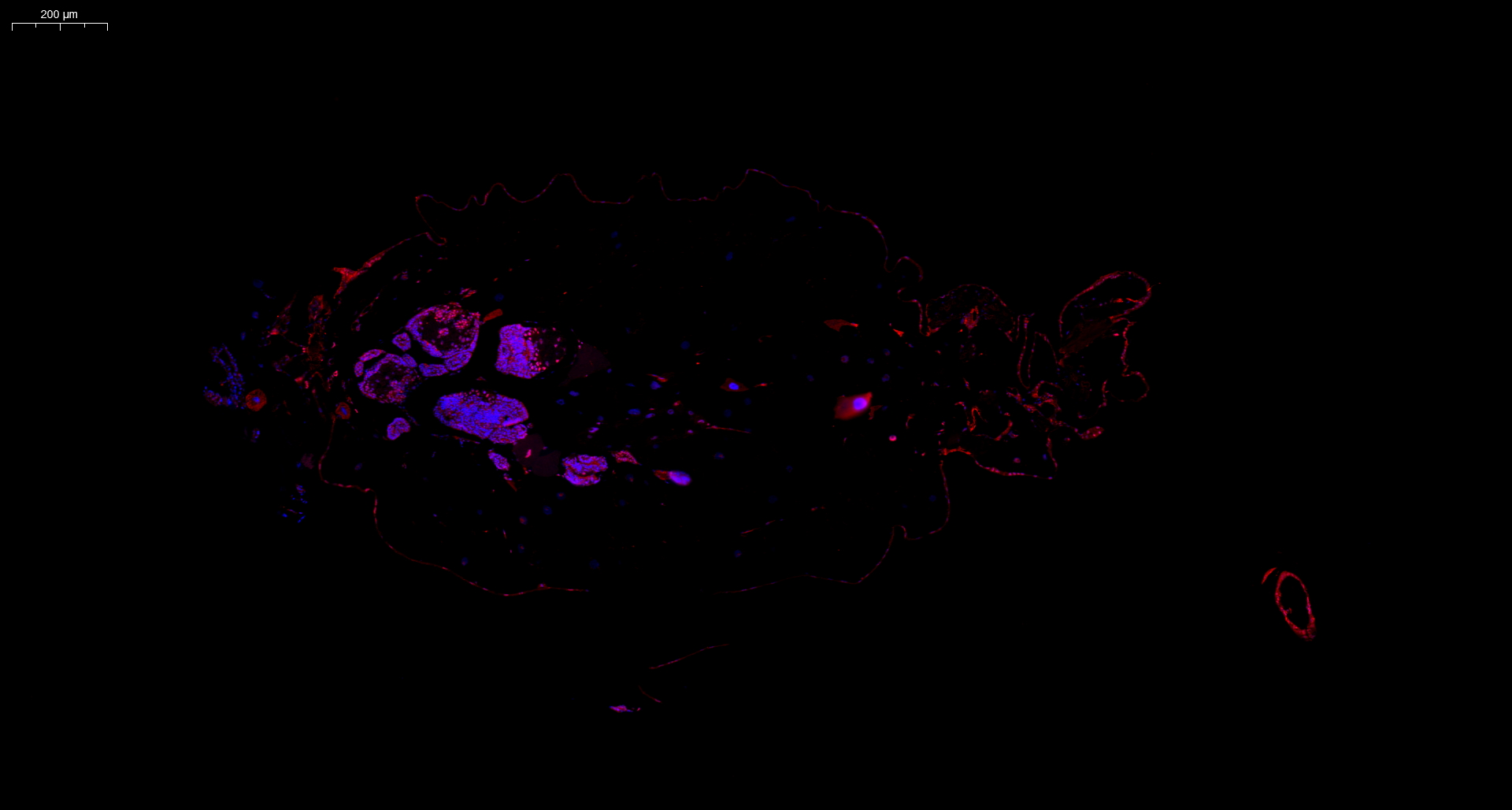

Supplement: Supplementary file 2 [file Data_Sheet_2.ZIP › mothers/T.repens-ck_7.0x-3.jpg]

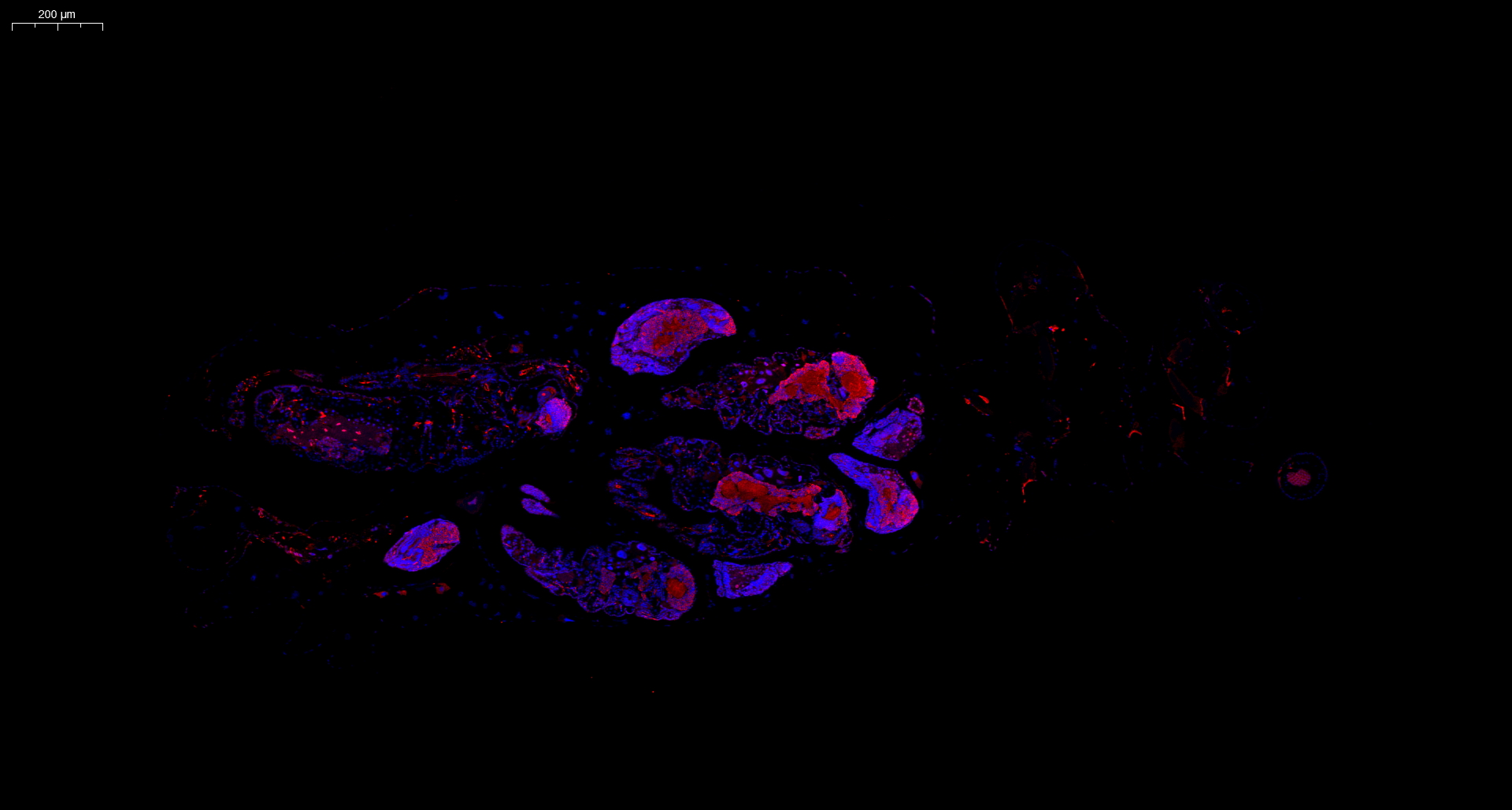

Supplement: Supplementary file 2 [file Data_Sheet_2.ZIP › mothers/T.repens-RNAi_7.0x-1.jpg]

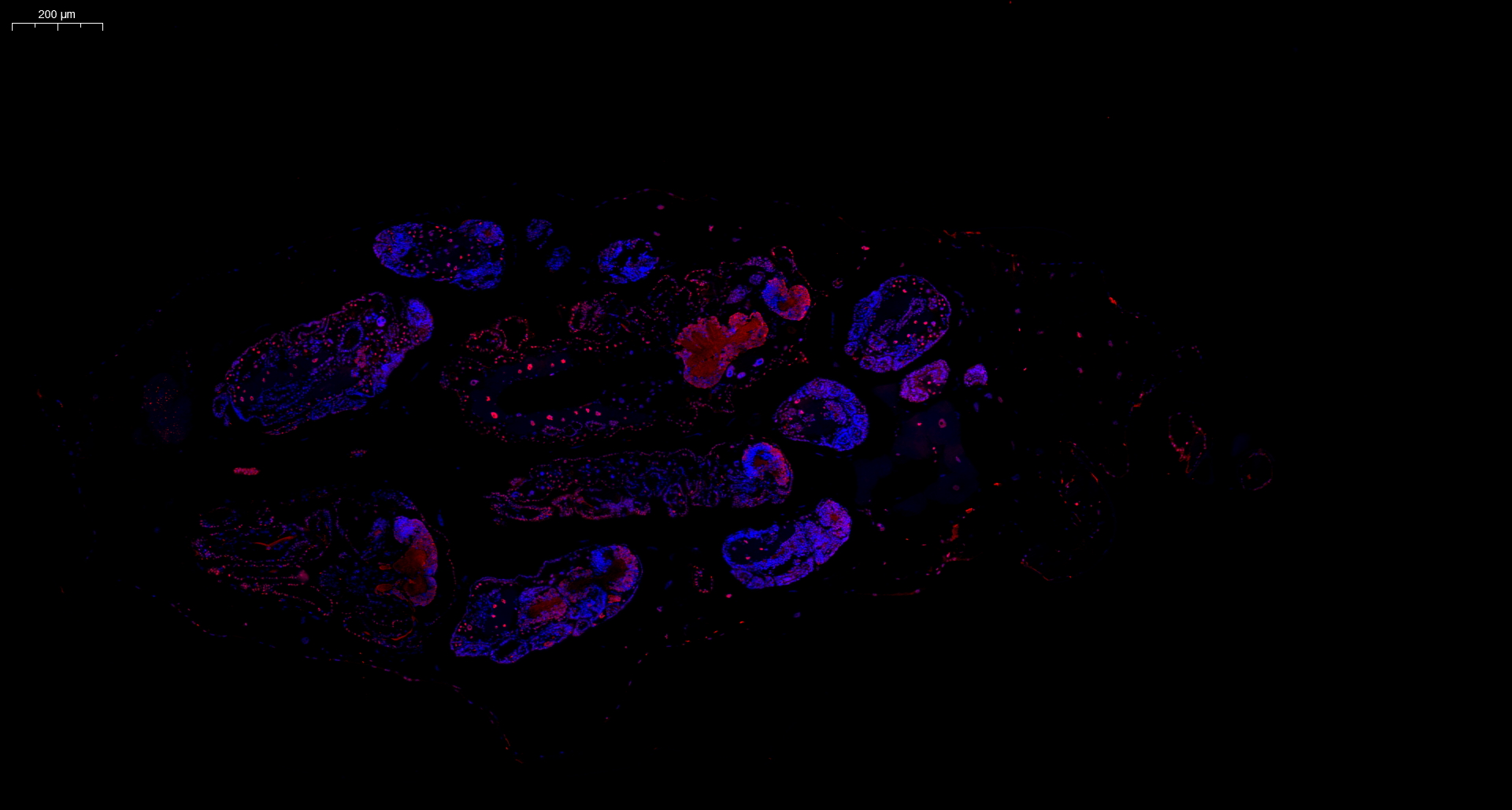

Supplement: Supplementary file 2 [file Data_Sheet_2.ZIP › mothers/T.repens-RNAi_7.2x-2.jpg]

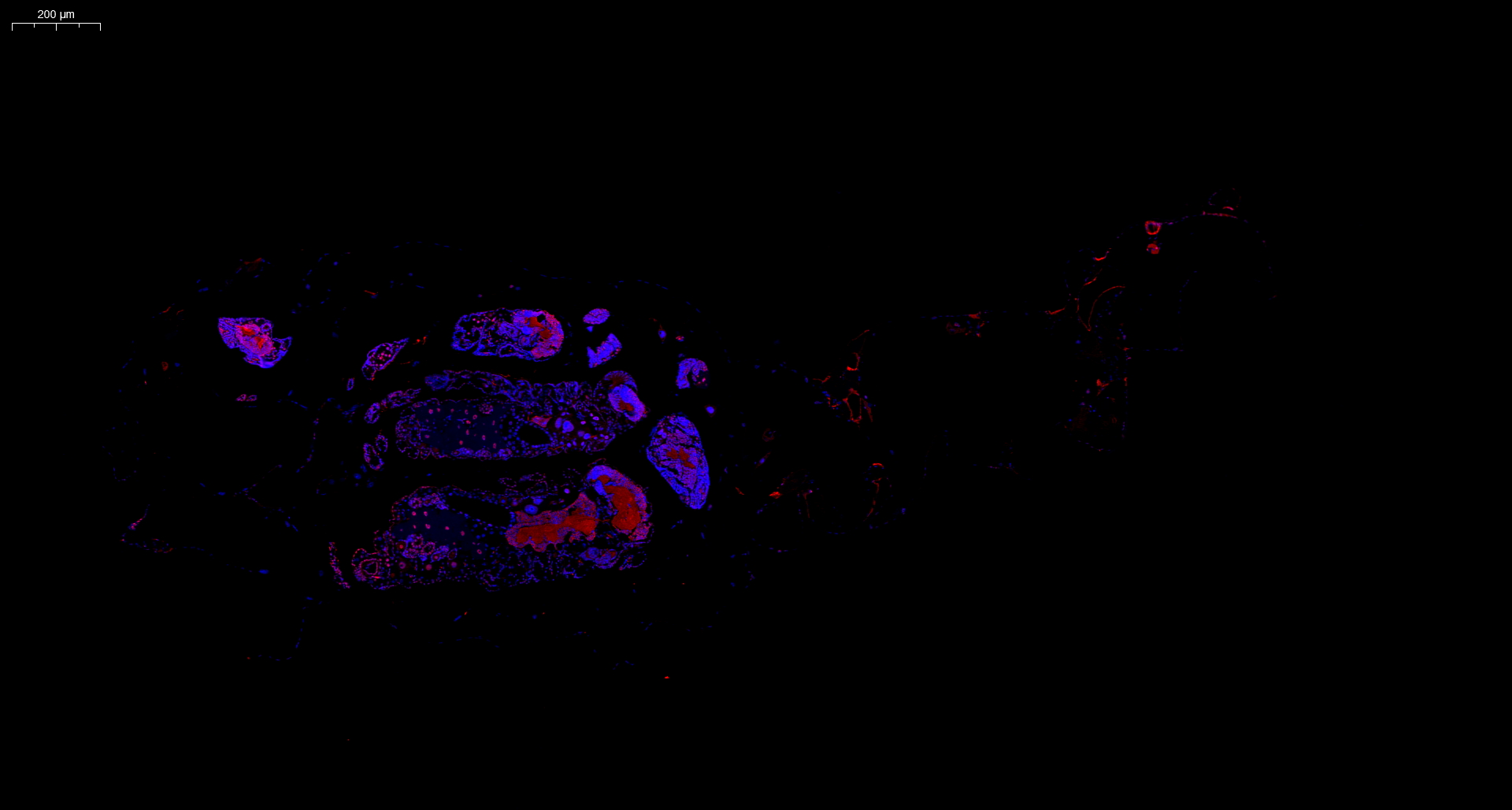

Supplement: Supplementary file 2 [file Data_Sheet_2.ZIP › mothers/T.repens-RNAi_7.2x-3.jpg]
