# Supplementary material for: The L-DOPA/Dopamine Pathway Transgenerationally Regulates Cuticular Melanization in the Pea Aphid Acyrthosiphon pisum
Source: Front Cell Dev Biol. 2020 May 5;8:311. doi: 10.3389/fcell.2020.00311 (PMC7214743; doi:10.3389/fcell.2020.00311)
Supplement: Supplementary file 3 [file Data_Sheet_3.PDF]

The L-DOPA/dopamine pathway trans-generationally regulates cuticular melanization in the pea aphid *Acyrthosiphon pisum*

[1]Yi Zhang; [1]Xing-Xing Wang; [1]Hong-Gang Tian; [1]Zhan-Feng Zhang; [1]Zhu-Jun Feng; [1]Zhan-Sheng Chen and [1]Tong-Xian Liu

[1] Key Laboratory of Integrated Pest Management on Crops in Northwestern Loess Plateau, Ministry of Agriculture, Northwest A&F University, Yangling, Shaanxi, 712100, China

S1. Materials and Methods

S1.1. dsRNAs synthesis, delivery and transcriptional analysis

dsRNAs synthesis

The dsRNA of the *A. pisum tyrosine hydroxylase* (*APTH*) gene was prepared using the T7 RiboMAX system (Promega Corporation, Madison, WI, USA). The dsRNA of the *lymphotoxin-alpha* gene (*lta*; Gene ID: 16992) of *Mus musculus* was used as a control. The primers used to synthesize *APTH* and *mus-lta* dsRNAs (designated as ds-TH1, ds-TH2, and ds-lta, respectively) are shown in Table S1 and Fig S1.

dsRNAs delivery

The dsRNAs were injected into *A. pisum* according to the method described by Barron and Zhang using a Nanoject II auto-nanoliter injector (Drummond Scientific Company, Broomall, PA, USA) fitted with a glass needle (P-97 micropipette puller; Sutter, Novato, CA, USA; pulling program: Pull = 100; VEL = 100; Time = 100). The dsRNA solution (6,000 ng/μL; 200 nL/aphid) was injected into the ventral thorax of 3-day-old adults through the fissure at the base of the hind legs (T3 segment) (Fig S4).

Aphids during the host transition period were used for the RNAi experiments. New-born aphids (about 100 individuals for each experiment) on *V. faba* were immediately transferred and reared on *T. repens* until they reached the adult stage. Individuals at 3 d post-molting were prepared for dsRNA injection. The injected aphids were transferred to plastic cells in 24-well plates containing *T. repens* leaves and reared for further experimentation (at least 80 survived individuals were selected for transcriptional analysis, and at least 30 survived individuals were selected for reproduction experiment each time).

Fully adapted *A. pisum* (more than 50 generations) reared on the two hosts were also treated with dsRNAs. For observation of parent generation phenotypes, fourth-instar individuals (12 h after molting) were prepared for dsRNA injection.

RNA extraction and transcriptional analysis

Mother generation

The injected aphids were reared for 5 d. Specimens of mother aphids were collected daily and dissected in liquid nitrogen between the T1 and T2 segments for *APTH* expression analysis. Intact bodies were also collected from each treatment for transcriptional analysis. Transcript levels were measured using six independent replicates from ten aphids of each replicate.

More replicates performed to analyze the *APTH* expression of abdomen under RNAi. Abdomen of each treated aphids (after 48 hours) were dissected and collected for further transcriptional analysis. nine independent replicates were prepared for each treatment from ten aphids of each replicate.

Daughter generation

Newly hatched nymphs from treated mothers were collected within 30 min after laying and flash-frozen in liquid nitrogen. Abdominal segments were removed before RNA extraction. Nymph samples were collected for 3 d after the mothers had been treated. Transcript levels were measured using six independent replicates from 20 nymphs of each replicate.

The aphid samples were frozen in liquid nitrogen immediately after collection. RNA was extracted using RNAiso Plus (TaKaRa Bio Inc., Kusatstu, Shiga, Japan) and the cDNA was synthesized using a PrimeScript RT reagent kit and gDNA Eraser (TaKaRa Bio Inc., Kusatstu, Shiga, Japan).

Quantitative reverse-transcription polymerase chain reaction (qRT-PCR) was performed using SYBR Premix Ex Taq II (TaKaRa Bio Inc., Kusatstu, Shiga, Japan) in an IQ-5 system (Bio-Rad Laboratories, Hercules, CA, USA). The primers were designed using NCBI's Primer-BLAST (http://www.ncbi.nlm.nih.gov/tools/primer-blast/index.cgi?LINK_LOC=BlastHome) (Table S1).

S1.2. L-DOPA and dopamine extraction and assay

The injected aphids were reared (*T. repens*) for 72 h after treatment and subsequently collected for LC/MS analysis. Newly hatched nymphs (with in 30 min) laid between 72 h and 96 h after treatment were also collected and prepared for LC/MS analysis. Ten micrograms of aphids were placed in a 1.5-mL centrifuge tube and ground in 1 mL extraction solution (0.05 M HCl in 50% v/v ethanol). The homogenate was centrifuged at 20,000 × *g* and 4°C for 10 min. The supernatants were analyzed using an LTQ XL linear ion trap mass spectrometer (Thermo Fisher Scientific, Waltham, MA, USA). Data-dependent tandem mass spectrometry (MS/MS) was used to identify, quantify, and fragment L-DOPA and dopamine. The masses of L-DOPA and its dopamine precursor, product ions, full-scan- and daughter-ion MS spectra, and their selected ion retention times (min) are shown in Table S2 and Fig S9. All data were acquired and processed using Xcalibur v. 2.1 (Thermo Fisher Scientific, Waltham, MA, USA). Quantification was achieved by comparison with a mixture of L-DOPA and dopamine external standards of known concentrations. Each treatment was conducted with 20 replicates (Ten micrograms of aphids of each replicate).

S1.3. Cuticle morphology analysis

HE staining

Histological sectioning staining and immunofluorescence were performed at the second thoracic (T2) segment of third-instar *A. pisum*. The samples were fixed in 10% (v/v) buffered formalin overnight, dehydrated, embedded in paraffin, and sectioned. Slides were prepared by soaking in xylene twice for

20 min, 100% alcohol twice for 5 min, and 75% alcohol for 5 min, followed by rinsing with water. The slides were then immersed in hematoxylin solution for 3–5 min and rinsed with water. The sections were differentiated with acid alcohol and rinsed again, treated with ammonia solution, and rinsed in slowly running tap water, and then stained in eosin in 85% alcohol for 5 min, 95% alcohol for 5 min, and eosin for 5 min. The sections were dehydrated three times with 100% alcohol (each for 5 min), treated with xylene twice (each for 5 min), and mounted with resin. Digital images were acquired using a Nikon DS-Ri1 camera (Nikon, Tokyo, Japan), a Nikon 80i microscope system (Nikon, Tokyo, Japan), and Nis-Elements v. 3.22.14 (Build 736, Nikon, Tokyo, Japan). Cuticle thickness was determined from the digital images.

Immunofluorescence

Mothers (wingless adults) treated with ds-TH and ds-lta were prepared for immunofluorescence experiments, and all samples were collected 48 hours after ds-RNA injection. Transverse longitudinal sections from head to tail of *A. pisum* samples, were stained with anti-rabbit TH IgG antibody. The transverse sections were initially placed in a microwave oven for antigen retrieval in a repair kit filled with EDTA antigen retrieval buffer (pH 8.0). After cooling, the slides were placed in PBS (pH 7.4) and washed three times on a decolorizing shaker (each for 5 min). After drying, a circle was placed around the tissue using an Imm-Edge pen, and an Auto-Fluo Quencher was added to the circle for 5 min, rinsed with running water for 10 min, and then incubated with BSA in the circle for 30 min. The primary antibody was mixed with PBS on a section, and the sections were incubated at 4ºC overnight. The slides were then placed in PBS (pH 7.4) and washed three times (each for 5 min). The secondary antibody was added to the circle and incubated for 50 min at room temperature. The slides were then placed in PBS (pH 7.4) and washed three times (each for 5 min). After drying, DAPI stain was added, followed by incubation at room temperature for 10 min. The slides were then placed in PBS (pH 7.4) and washed three times (each for 5 min). Finally, the sections were dried and then sealed with an anti-fluorescence quenching capsule. All captured images were under Cy^{TM}3 fluorescence Filtering at default settings (brightness, contrast and saturation).

Abnormal and control nymphs obtained from the ds-TH and ds-lta treatments, respectively, were prepared for immunofluorescence experiments. Transverse crosscutting sections at the second thoracic (T2) segment of third-instar nymphs of *A. pisum* were stained with anti-rabbit TH IgG antibody. The protocol was the same as that detailed for the mothers above.

S1.4. Tests of dopamine transition signals of nervous system

Injection of dopamine/L-DOPA biosynthesis inhibitor

Metirosine (α-methyltyrosine, tyrosine hydroxylase enzyme inhibitor; No.1443205, Merck KGaA, Darmstadt, Germany; dissolved in saline) was prepared and injected into *A. pisum* (0.1 mg/mL of 0.9% saline, 100 nL for 30 mother aphids) according to the method described above (S1.1). Daughters of treated aphids were reared for phenotypic detection.

Injections of dopamine receptor antagonists in nervous system of *A. pisum*

Three dopamine receptor proteins were observed from the NCBI protein database of *A. pisum*: dopamine receptor 1-like (D1, XP_016661804.1), dopamine receptor 2-like (D2, XP_008178377.1), and dopamine receptor 4-like (D4, XP_016662867.1). Accordingly, SCH23390 (antagonist of the D1; No. D054, Merck KGaA, Darmstadt, Germany; dissolved in saline), Sulpiride (antagonist of the D2 and D3; No. S8010, Merck KGaA, Darmstadt, Germany; dissolved in saline), and Pimozide (antagonist of the D2, D3 and D4; No. P1793, Merck KGaA, Darmstadt, Germany; dissolved in 10% DMSO + 40% PEG300 + 5% Tween-80 + 45% saline) were selected for injections (1 mg/mL of 0.9% saline, 100 nL of each antagonist treatment with 30 mother aphids), and only the saline was used for the control. The methods were described above (S1.1), and daughters of treated aphids were reared for phenotypic detection.

128

S2. Tables and Figures

S2.1. RNA interference in pea aphid *Acyrthosiphon pisum*

Table S1. List of primers utilized in this study

	Forward (5’ to 3’)	Reverse (5’ to 3’)	PCR type
ds-TH1 (XM_008184777.3)	T7*+ TCAGATACAATGGCAGTAGC	T7+ CCTCTTCTTCAGTAAGTCCG	dsRNA
ds-TH2	T7+ ATGTCGTCCATTTGGAATCA	T7+ TATTCGAAAGCAATGTTGGC	dsRNA
ds-mus-lta (XM_006523731.4)	T7+ CACCCTCTCCACGAATTG	T7+ TAGAAGATGCTGCTGTTTCA	dsRNA
Q-APTH	ATACATACCGTTTCCGTCTG	TCGTCTAGTTTCGTTTGTGT	Q-RT-PCR (E=98.5%)
Q-APDDC (XM_001950520.5)	CTGACATACTGAGCGATTCA	TCTGGCAAGCCTATCATTTT	Q-RT-PCR (E=103.1%)
Q-cuticle protein 1(XM_001949658.5)	CAGTCGCAGCTACAGTGTCT	CGCGAAAACGATTGCGAGTA	Q-RT-PCR (E=109.1%)
Q-cuticle protein 7(XM_001946681.5)	GGACGTATGGAAGGGATGCT	GCGGCGAGGATGATGAATTT	Q-RT-PCR (E=102.5%)
Q-cuticle protein (NM_001163201.1)	CGTCCTCAAGGGGACTCCAT	ATGCGACCTGTATCCTTGCC	Q-RT-PCR (E=92.5%)
Q-cuticle protein 19(XM_029488103.1)	TCTGTTTCGTACAGTATCTCTGTT	GCGAAAAACGACGATTGGAGA	Q-RT-PCR (E=97.7%)
Q-cuticle protein 19.8(XM_003247688.4)	TCAAGTCTCGCAGCGTCATT	GGTCGTGAACGGCGTAATTG	Q-RT-PCR (E=97.0%)
Q-DNAT (NM_001326676.1)	CGTGGAGTGCTCCAGTCATT	TTGTGTGAGGAGGCTGTGTC	Q-RT-PCR (E=100.9%)
Q-yellow-like (XM_001944914.5)	TGGACGTGAACTGTCATCCG	GTTGCATGCGTACGGCTAAA	Q-RT-PCR (E=91.4%)
Q-APRpl7 (Ref)	GCGCGCCGAGGCTTAT	CCGGATTTCTTTGCATTTCTTG	Q-RT-PCR (E=100.7%)

*T7 sequence: 5’-taatacgactcactataggg -3’

TGTCTTTTCATTCGCATCCGATTGCACGTGTGACGTGCGTTCTCATCCGCCGGTCGACGTAAATATACTCGTAATTGTT
TCGGGTTCTCAGCACATATAGCGACAAATAGAGGACTTATACCACTGTACCAGCCACGTTCAGTTATTCCGATTTTCCG
Q-TH
TCAAGCGCAAAATCCGAGAAGTCCTAAACCGGAACCAGGTA[illegible]GCACCTGCCATCATCGTTATTATACGCAC
GCCCTTGTCGTCGTATACGTAATTGTTGCCAGCCAGCAGAGT[illegible]CAT**ATACATACCGTTTCCGTCTG**TTGT
CGATTTCGAGAGGAAATCTAGTAGCCCGTGAAAATCGTTTCCGATCCGTCAGAAACGACCCAATATACGGCTATTTG
TAACTCTCGTCCGCCGCAGAGTTATTAAAAACC**ACACAAACGAAACTAGACGA**GTTGTCGACCCGCAATCAAACCG
TGACATTAGCTTTGACCGTAATTTGTTATTTTTCATTTATCCGCCGATAAATTCGATTTCGCCGCGACCCGCGATATTTCT
TGGTGATCATTTACACTGGAGTTTGGAGCTTACGGCATTTTGCTACACAGTTTCGGTAGAGTCAACGCCAAGAAACC
GCAA**TCAGATACAATGGCAGTAGC**AGCAGCCCAAAAGAGCCGAGAGATGTTCGCCATCAACAAGTCATATAGCATC
GAGAACGGATATCCAGCGAGGAGACGTTCTCTCGTAGACGACGCCAGATTCGAGACGATCGTCGTGAAGCAGACCA
AACATTCCGTTTTGGAGGAAGCCCGGCAATTAGCGAACGACGCTTCTTTAGCGACCGACGCAGCCGTAGCGCATTTC
GCCAGTCAACTTCAACGTCAACATCTTGATCAAATTAACGATCAATACGACGATCAAGTTTTTGAATCCGAAGGCAAA
ds-TH1
TCTATTGAACACGATCATGAAAACGATGTAGA[illegible]ATCATCTGAACCATCGTCCGACAATGACTCAGATG**CC**
GGACTTACTGAAGAAGAGGTGCTATTGGAGA[illegible]AGCCAAGACAAAGATGCGGAGATGGCTGTACAAAG
GGCTACTCTGGTATTGCGTCTGCGCGAAGGTATCAATTCACTCGCCCGGGTATTGAAAGTAATCGAAAATGGAAAAGG
CA**ATGTCGTCCATTTGGAATCA**AGGCCGTCTAAGGAACAAGGCGTTCAATTTGACATTTTGGCCAAAGTGGACATG
ACGAGAAAAGATCTATTAGCATTAATGAAGACACTCAGACAAAGCTCTACATTAGCAAGCATTACGATTCTAGCCGAA
GACAACATCAACGTCAAGAACCCGTGGTTCCCGAGACATGCCCGTGACTTGGACAACTGCAACCACTTGATGACCA
ds-TH2
AATACGAACCGGAATTGGACATGAACCACCCC[illegible]GACAAAGTTTACCGCTCCCGCAGGAAGGAAATC**GC**
CAACATTGCTTTCGAATATAAATTCGGCGACT[illegible]TTATCGAATACACTGCTGACGAAGTCAGTACATGGA
CCGCTGTTTTCAACAACGTGTTGGACCTGATGCCCAAGCACTTCTGCATGGAATACAGGGACGCTTTCAAGATGCTA
CAGGACGATAACATATTCACTGCTGACAAGATCCCACAACTTAAGGATATGAACGAATTCTTGAAAAAGCACACAGG
ATTCACTCTCCGGCCGGCGGCTGGTTTGTTGACGGCCCGAGACTTCCTCGCCAGCCTCGCGTTCCGTGTGTTCCAAA
GCACGCAGTACATTCGTCACAGCACATCACCATTCCACACACCTGAACCGGATTACATCCACGAAGTCCTCGGACAC
ATGCCTTTATTGGCCGATCCGAGTTTCGCTCAGTTCTCACAAGAACTCGGTTTAGCGTCTCTTGGAGCTTCTGACGAA
GAAATCGAAAAACTGTCAACTGTGTACTGGTTCACGATCGAGTTCGGTCTCTGCAAGGAACAAGGAGAGGTGAAGG
CCTACGGCGCAGGACTGCTGTCTGCTTACGGTGAACTTTTGCACGCTGTTTCTGGCAAACCCGAACTTCGACCATTC
GAACCATCAATCACAGCCGTCCAACCATACCAAGATCAAGAATACCAGCCAATTTATTTCGTCGCTGAAAGCTTCGAA
GACGCCAAAGACAAATTCAGGAAATGGGTTTCATCCATGTCACGACCATTCGAAGTCCGTTATAACCCACACACCCA
GAGAGCAGAAATCCTAGACTCAGTTGAACAGCTCAACAATCTCATGACACAATTGAACTTGGAAATGTTGCATCTAA
ACACCGCACTGAACAAGTTGAAAATGAGATCCGGTTAA

Fig S1. Sequence of *TH* in *Acyrthosiphon pisum*, and two designed dsRNA were marked (positions of primers were marked in bold); fragment for qRT-PCR test was also marked in the sequence

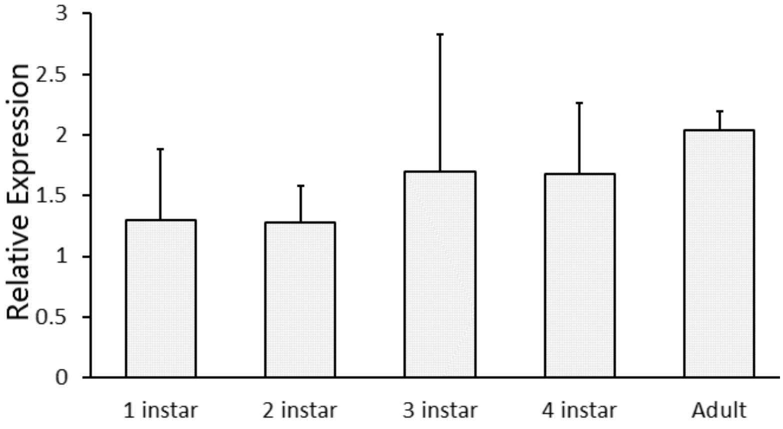

Fig S2. Development patterns of *TH* gene at five developmental stages at hosts alternation condition in head of pea aphid *Acyrthosiphon pisum*. Each value represents the mean±SEM, no significant difference observed based on ANOVA test.

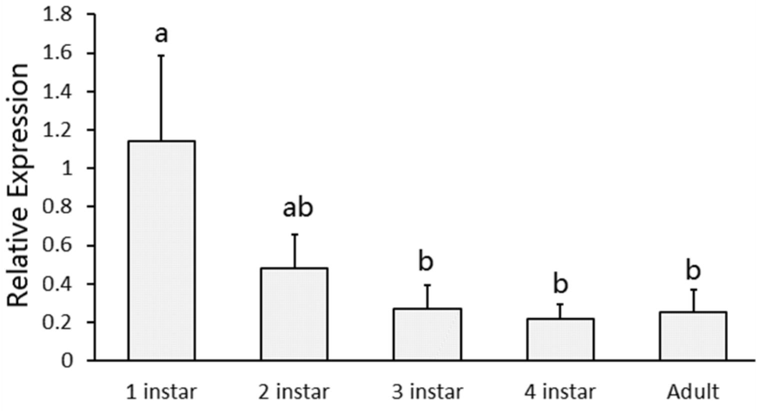

Fig S3. Development patterns of *TH* gene at five developmental stages at hosts alternation condition in abdomen of pea aphid *Acyrthosiphon pisum*. Each value represents the mean±SEM and no significantly different observed ($P < 0.05$, ANOVA, Duncan's test).

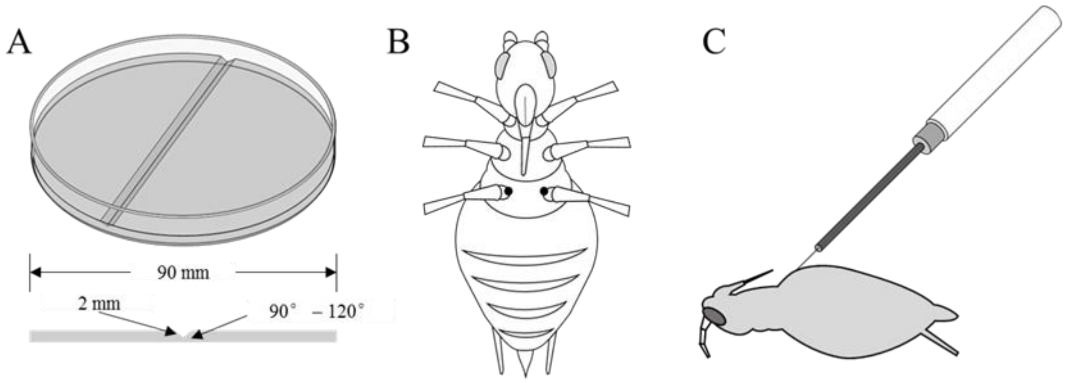

Fig S4. Aphid injection device. Operation pad for injection (with 1% agar, A). Injection (dark spots) position (B) and injection direction (C).

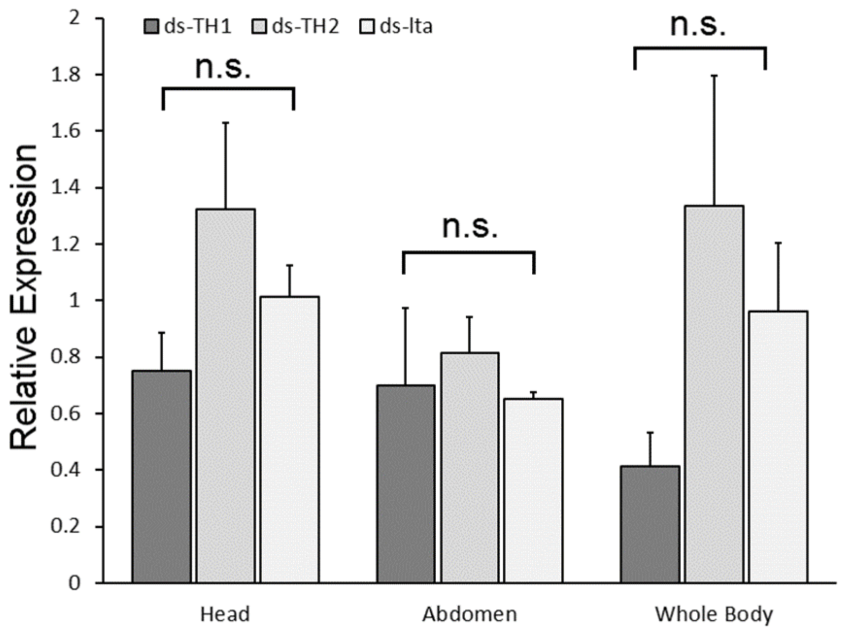

Fig S5. RNAi experiments in wingless pea aphid *Acyrthosiphon pisum*. *DDC* expression in the head, abdomen and whole body of wingless pea aphid under *TH* RNAi after 48 hours. Each value represents the mean±SEM and no significantly different observed (ANOVA, Duncan’s test).

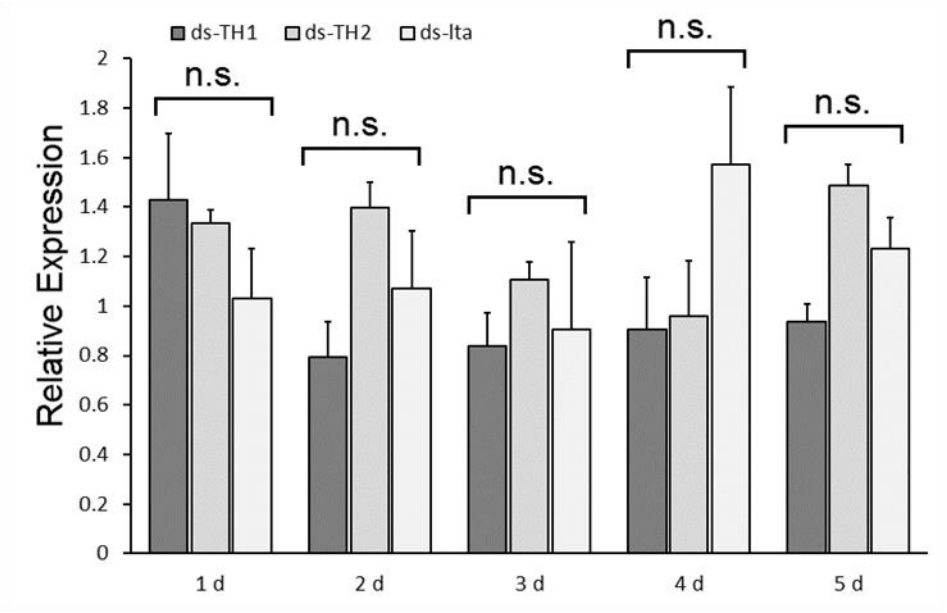

Fig S6. *DDC* expression in head after *TH* dsRNA injection for five days. Each value represents the mean±SEM and no significantly different observed ($P < 0.05$, ANOVA, Duncan’s test).

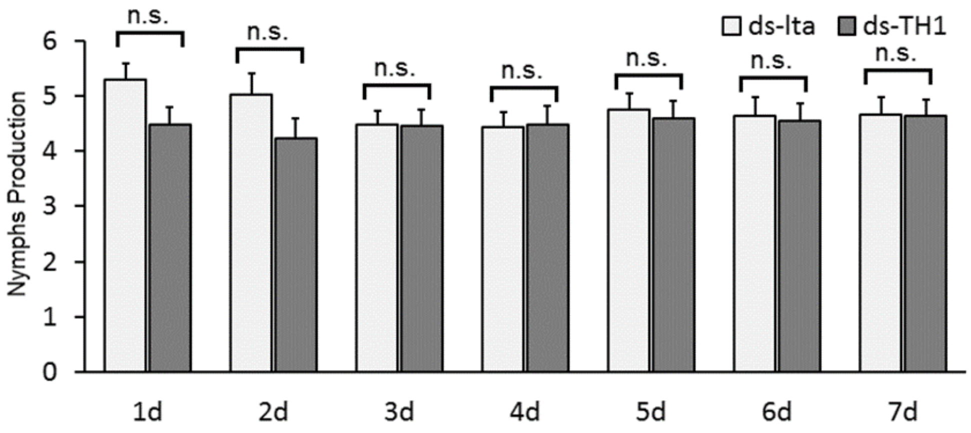

Fig S7. Nymphs’ production changes under *TH* RNAi (ds-TH1). Monitoring lasted for seven days. Each value represents the mean±SEM and no significantly different observed (Student's *t*-test).

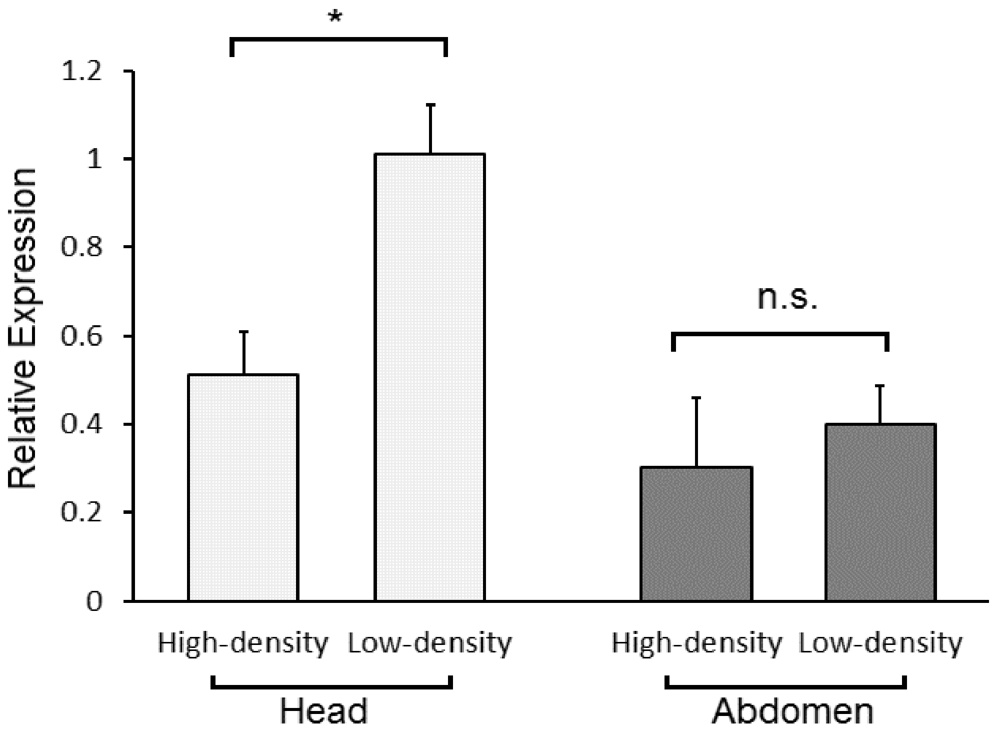

Fig S8. Expression differernces of *TH* in head and abdomen of pea aphid *Acyrthosiphon pisum* with different densities (high: thirty aphids/ 2-week old faba bean; low: five aphids/ 2-week old faba bean). Each value represents the mean±SEM. * indicate significantly different at $P < 0.05$ (Student's *t*-test).

S2.2. L-DOPA and dopamine assay by LC/MS

Table S2. Masses of precursor and product ions and collision energy for liquid chromatography-electrospray ionization tandem mass spectrometry (LC-ESI-MS-MS) analysis of L-DOPA and dopamine

Compounds	Precursor ion (M+H)+(*m/z*)	Product ion (*m/z*)	Collision energy (eV)
L-DOPA	198	152	35
Dopamine	154	137	35

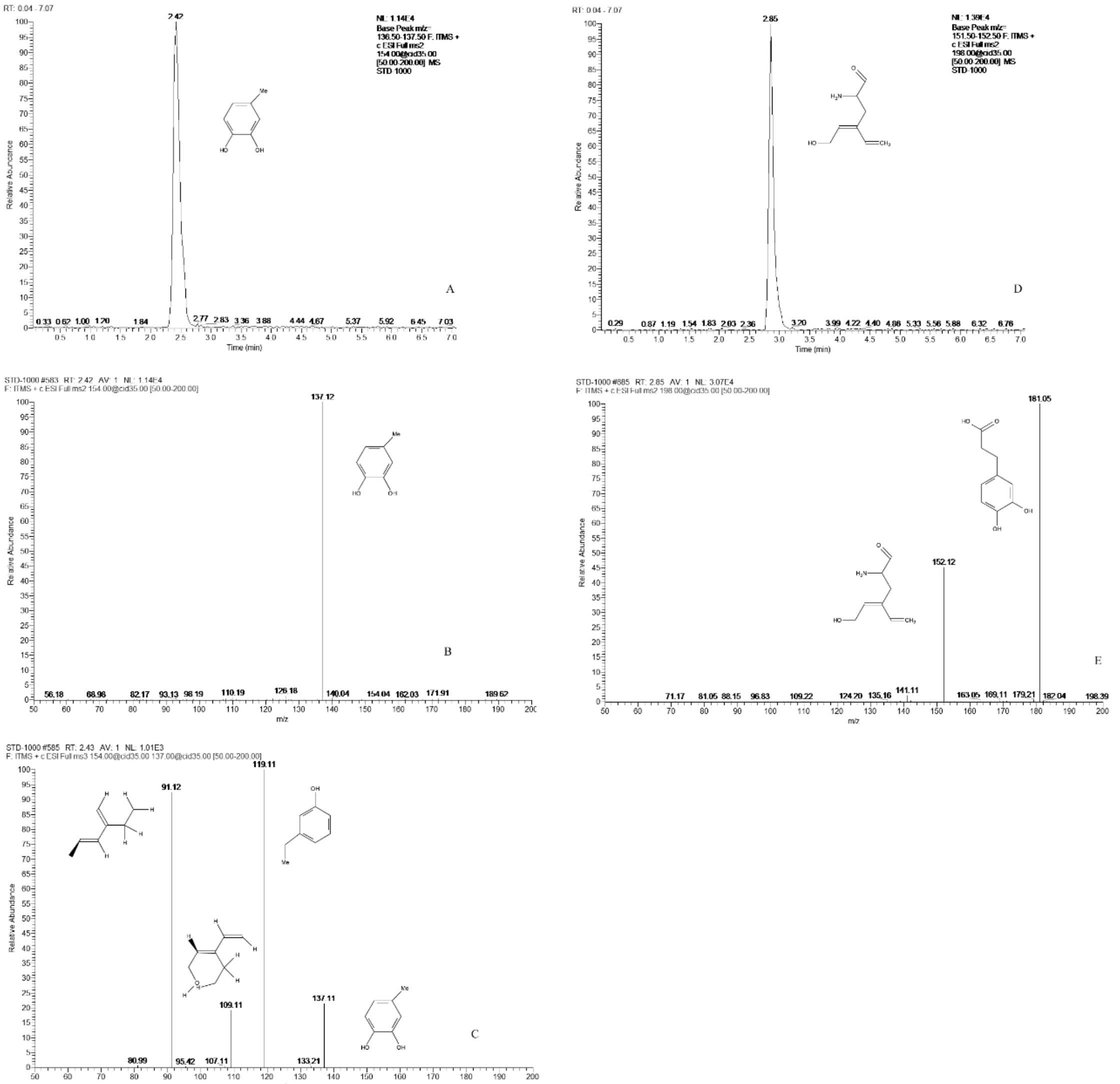

Fig. S9. Full daughter scan MS Spectra and selected ion retention time (min) of dopamine (A, B and C) and L-DOPA (D and E). MS Spectra information was referred to the database of METLIN (Scripps Center for Metabolomics METLIN: Metabolite and Tandem MS Database http://metlin.scripps.edu/terms.php). Liquid chromatography separations were carried out with XTerra MS C18 Column (125Å pore size, 5 µm, 150 mm ×4.6 mm; Waters Corp., Milford, MA, USA). Elution was performed by applying a three-step gradient: 100% A for 8 min, 0-100% B linear for 2 min, 100% B for another 5 min, and 0–100% A linear for 1 min, holding the system at 100% A for 8 min. Mobile phase A was an aqueous solution containing 15% methanol and 0.1% formic acid; and mobile phase B was 100% methanol with 0.1% formic acid. The flow rate was 0.6 mL/min. The mass spectrometer was set in the positive electrospray ionization mode. Nitrogen was used as the sheath gas (40 arbitrary units) and auxiliary gas (10 arbitrary units). The spray voltage was set at 4.5 kV and the ion transfer capillary temperature was 275°C.

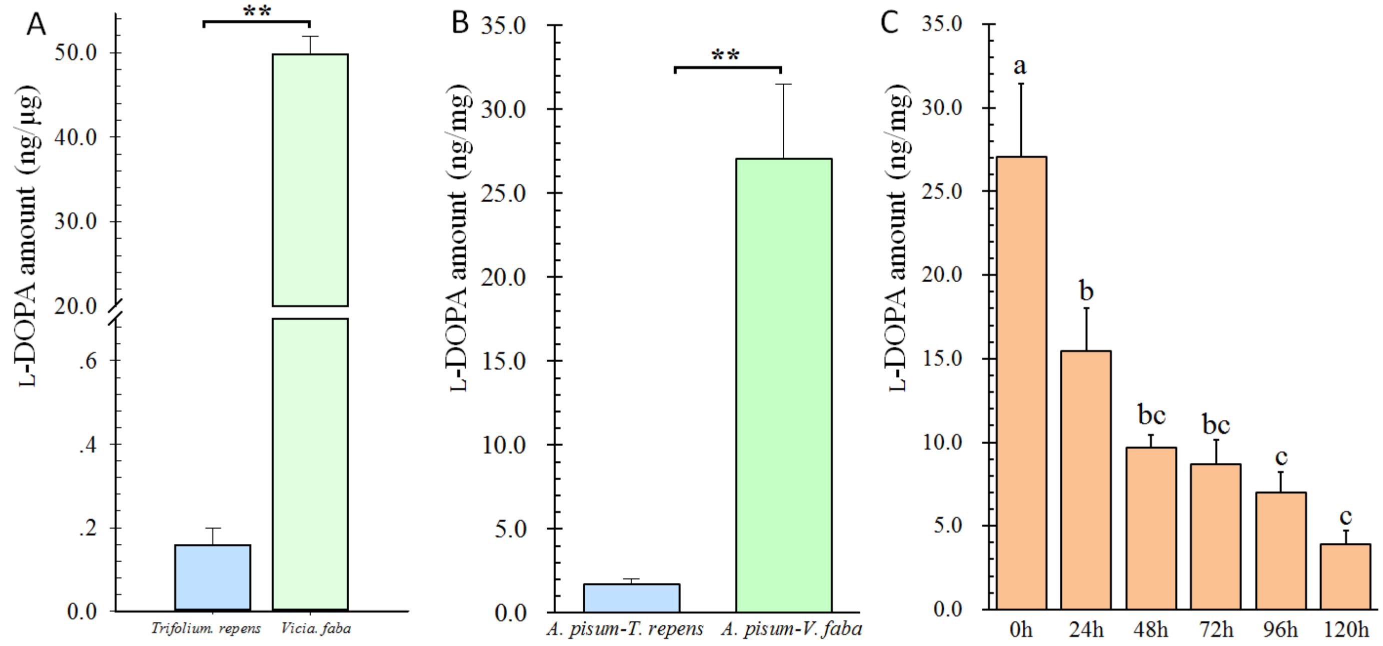

Fig S10. L-DOPA amounts assay in hosts plants and pea aphids. (A) L-DOPA differences between *Trifolium repens* and *Vicia faba*. (B) L-DOPA differences of *Acyrthosiphon pisum* on *T. repens* and *V. faba*. (C) L-DOPA dynamic changes at hosts alternation condition in 120 hours. Each value of A and B represents the mean ± SEM; Student's *t* test (***P* < 0.001); different letters within the same figure of C indicate significant differences in values (ANOVA, Duncan's test, *P* < 0.05).

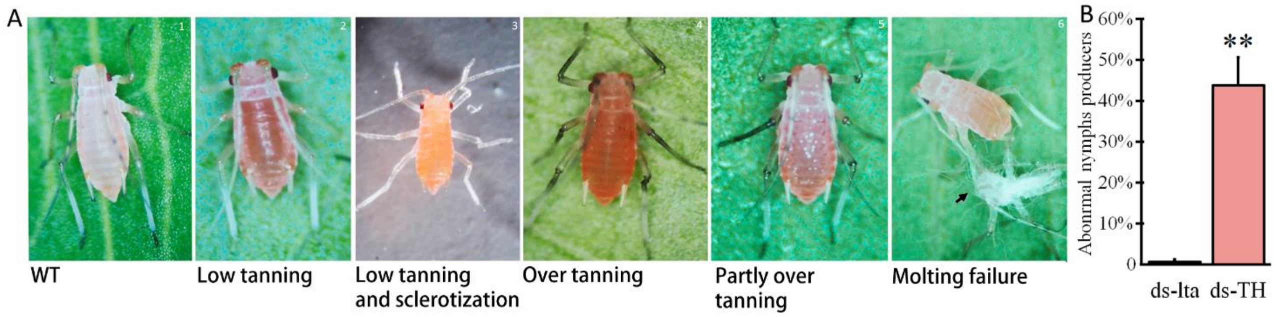

Fig. S11. Abnormal melanization in the pea aphid *Acyrthosiphon pisum*. Proportions of abnormal melanization in all nymphs after 7 d of monitoring producers of abnormal nymphs. (A) Appearances of abnormally melanized aphids; arrow indicates fungal mycelium in the exuvia. (B) Proportions of abnormal nymph producers among all ds-TH-treated mothers. Each mother bearing more than one abnormal nymph was regarded as an abnormal nymph producer. Each value represents the mean ± SEM; Student's t-test (***P* < 0.001).

More than 40% of the ds-TH-treated mothers were transformed into producers of abnormal nymphs. This conversion rate was significantly higher than that for mothers in the ds-lta control group (*t* = -6.238, df = 10, *P* < 0.001; Figure S11B).

S2.3. Transcriptome sequencing

Table S3. Data analysis of transcriptome

Analysis	Software	Version	Parameters
Mapping	Tophat	v2.0.12	mismatch = 2
Gene ExprQuatification	HTSeq	v0.6.1	-m union
AS (alternative splicing)	rMATS	3.0.8	default
Novel Gene	cufflinks	2.1.1	default
SNP detection	GATK2	v3.2	QUAL < 30.0 && QD < 5.0
DiffExpr Analysis	DEGSeq	1.12.0	|log2foldchang|>1 && qvalue<0.005
	DESeq	1.10.1	padj<0.05
	edgeR	3.0.8	padj<0.05
DEG_GO Enrichment	GOSeq，topGO,hmmscan	Release2.12	Corrected P-Value<0.05
DEG_KEGG Enrichment	KOBAS	v2.0	Corrected P-Value<0.05
DEG_PPI	BLAST	v2.2.28	e-value = 1e-10 && string score >700
DEG_Trans_Factor	Plants：iTAK；Animals：AnimalTFDB	1.2	default

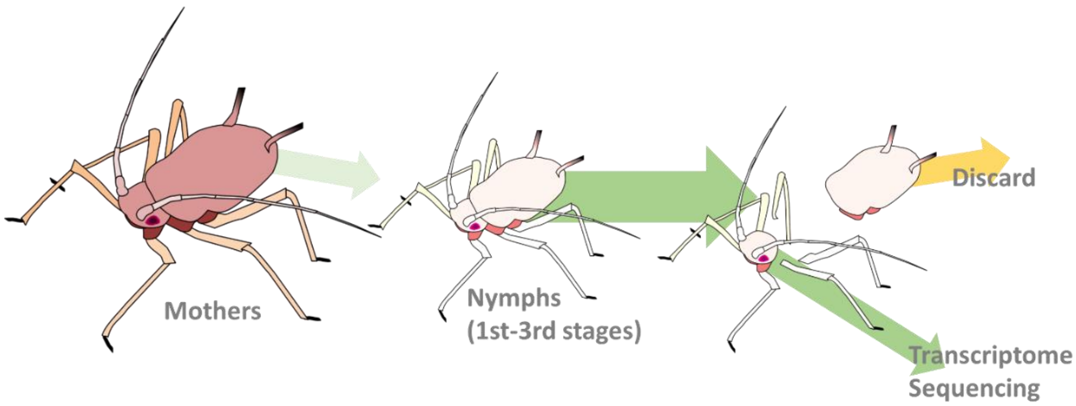

Fig S12. Profile of sample collection protcol for transcriptome sequencing.

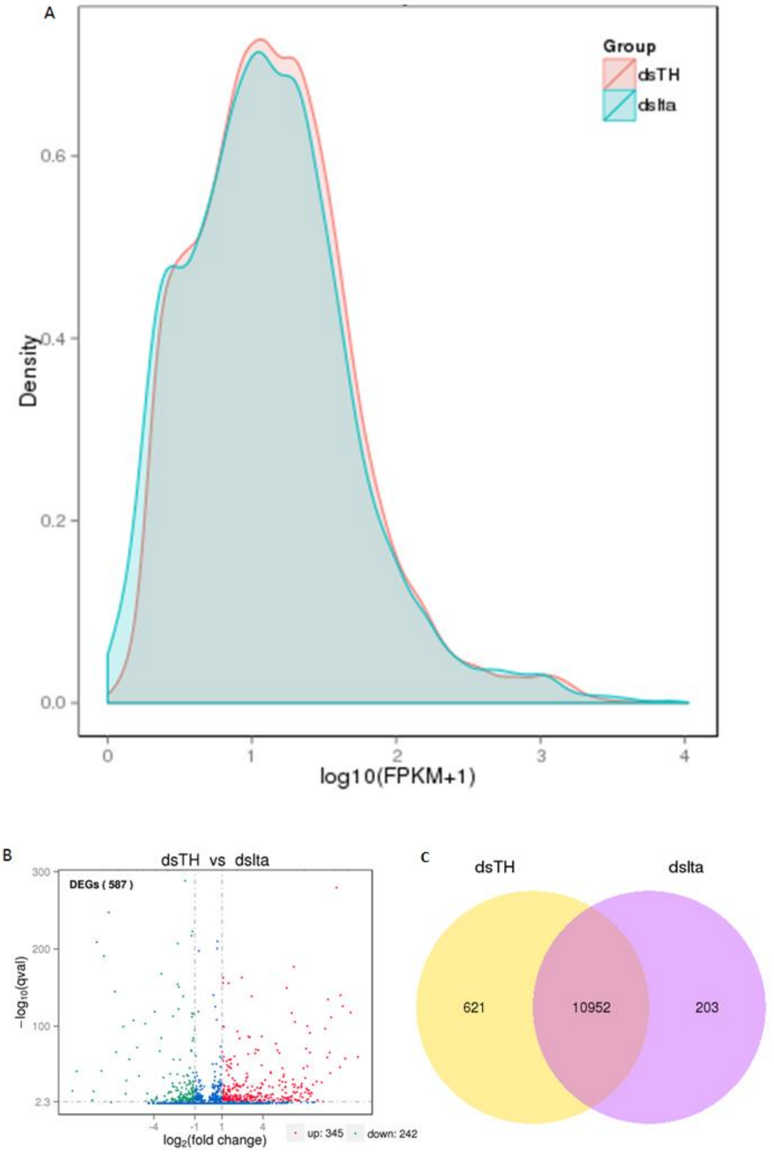

Fig S13. FPKM density distribution and different expression analysis (Volcano Plot: B, and Venn Diagram: C)

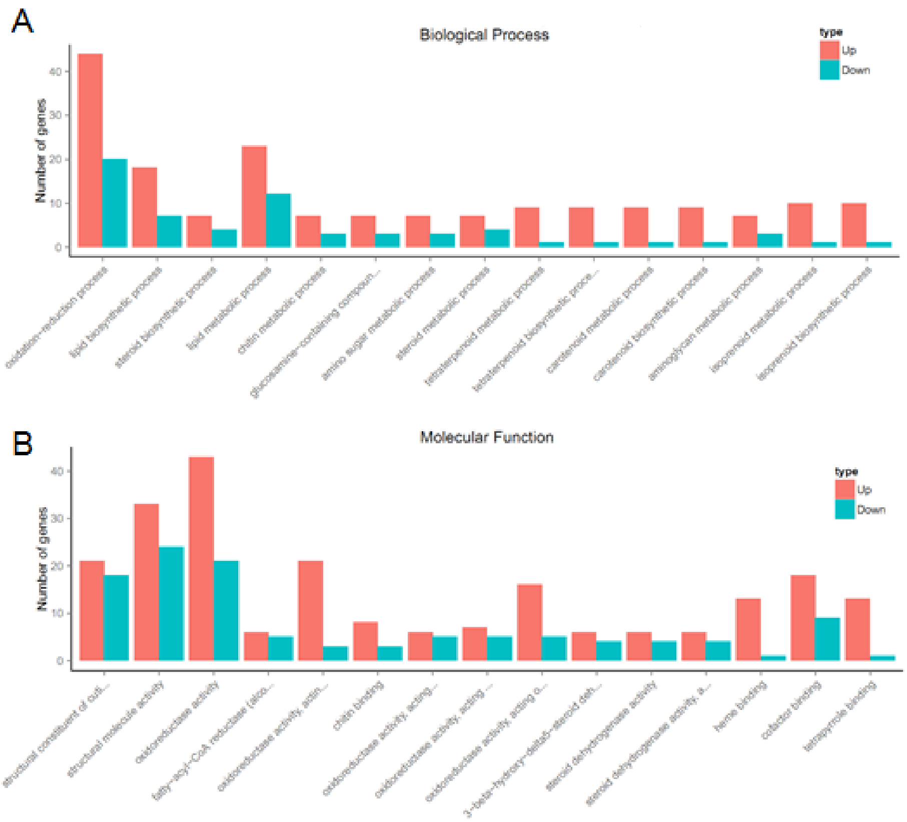

Fig S14. The most enriched GO terms (dsTH vs dslta) on Bological Process (A) and Molecular Function (B).

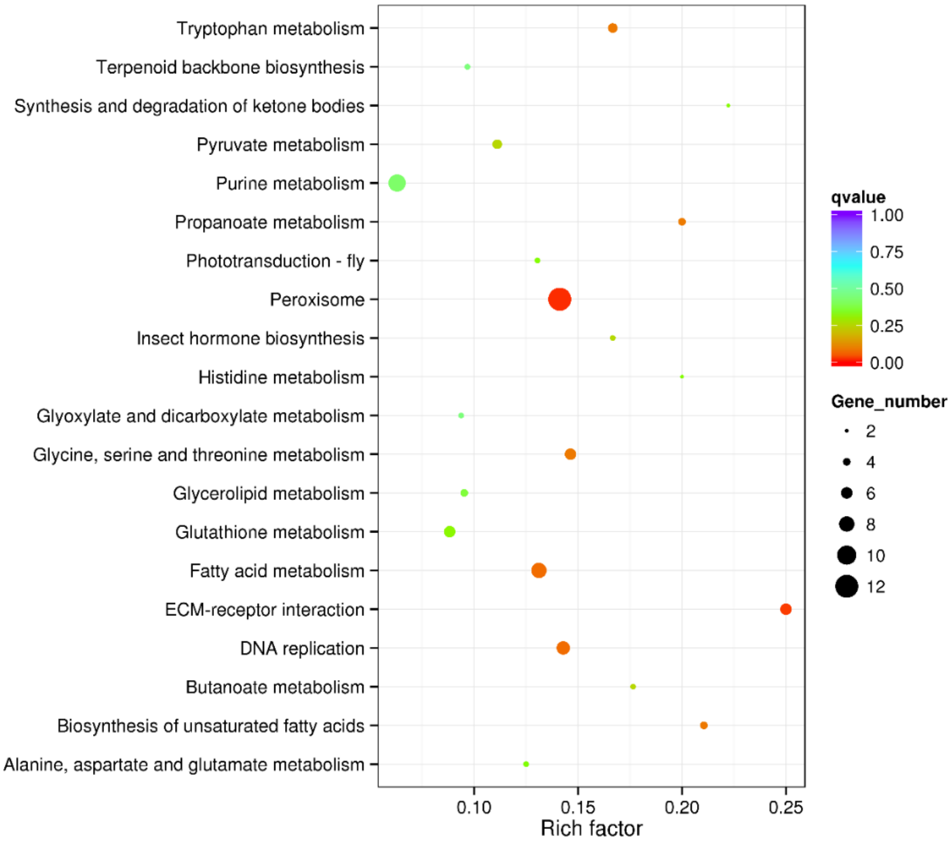

Fig S15. Statistics of pathway enrichment based on KEGG database. The point size indicates the number of genes expression different and colors indicate Q-value

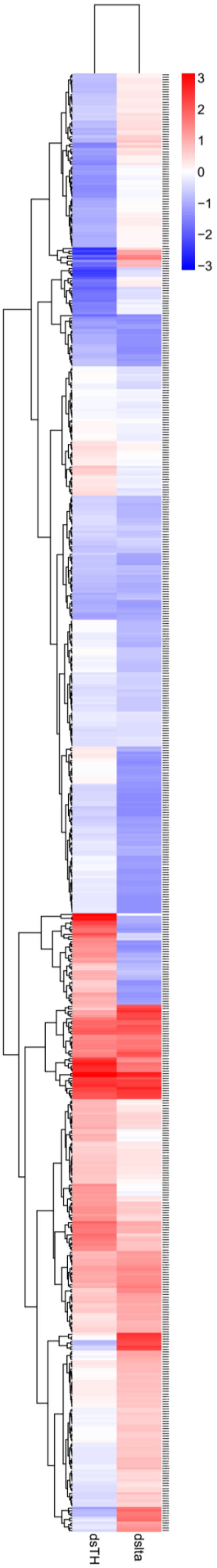
3
2
1
0
−1
−2
−3
dsTH
dsIta

Fig S16. Cluster analysis of differentially expressed genes

S2.4. Experiments of long-hosts-adapted aphids

4.1. TH-RNAi in long-adapted *V. faba* population (ds-TH1, 48 hours after injection)

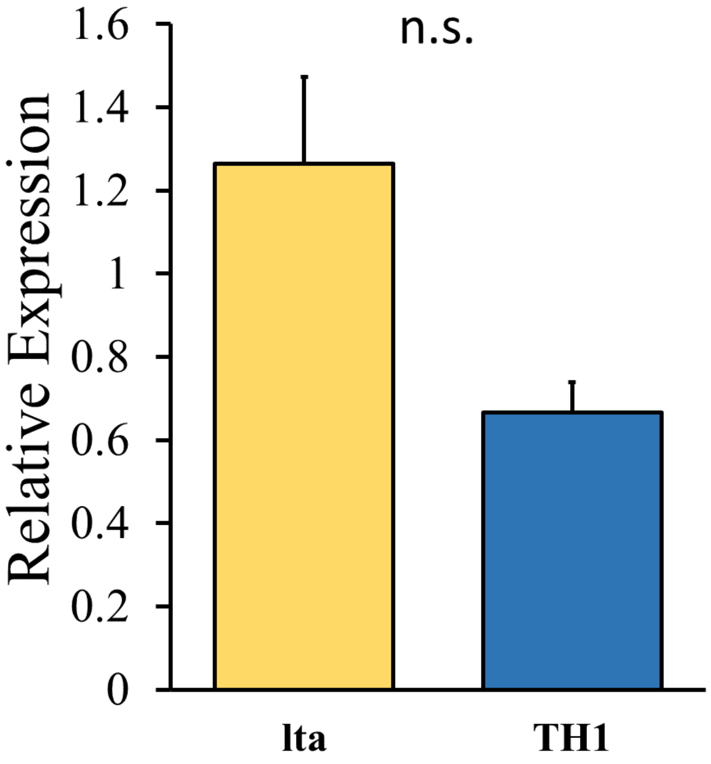

Fig S17. *TH* expression in head after *TH* dsRNA injection at 48 hours. Each value represents the mean±SEM and no significantly different observed (*P* = 0.053; Student's *t*-test)

4.2. TH-RNAi in long-adapted *T. repence* population (ds-TH1, 48 hours after injection)

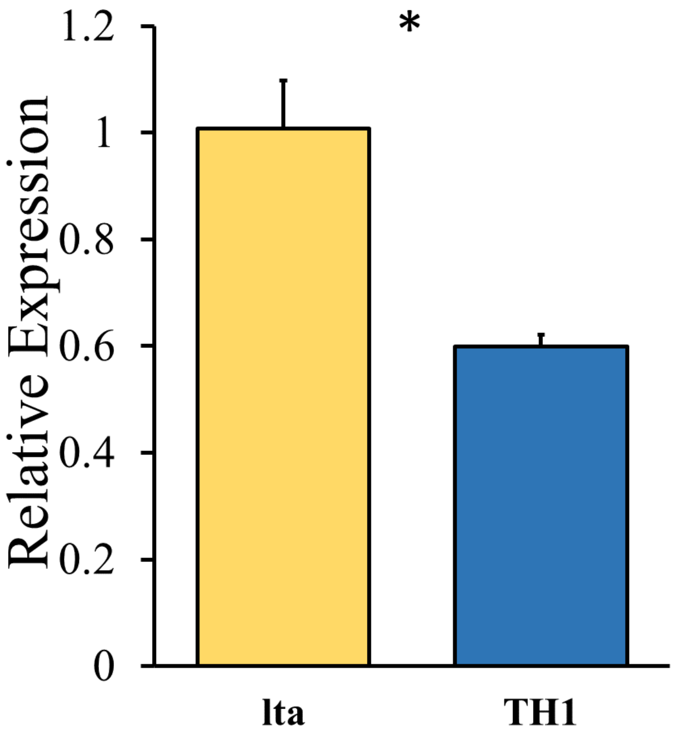

Fig S18. *TH* expression in head after *TH* dsRNA injection at 48 hours. Each value represents the mean±SEM. *$P < 0.05$ (Student's *t*-test).

S3. Other Files:

Video 1 (1 instar 50X): ZhangYi_Video1 .mp4

Video 2 (2 instar 20X): ZhangYi_Video2 .mp4

Images: original images.zip

RNA sequencing: Transcriptome sequencing.zip
